# Temporal trajectories of important diseases in the life course and premature mortality in the UK Biobank

**DOI:** 10.1186/s12916-022-02384-3

**Published:** 2022-05-27

**Authors:** Xianwen Shang, Xueli Zhang, Yu Huang, Zhuoting Zhu, Xiayin Zhang, Shunming Liu, Jiahao Liu, Shulin Tang, Wei Wang, Honghua Yu, Zongyuan Ge, Mingguang He

**Affiliations:** 1 Department of Ophthalmology, Guangdong Provincial People’s Hospital, Guangdong Academy of Medical Sciences, Guangdong Eye Institute, Guangzhou, 510080 China; 2 Guangdong Cardiovascular Institute, Guangdong Provincial People’s Hospital, Guangdong Academy of Medical Sciences, Guangzhou, 510080 China; 3 Centre for Eye Research Australia, Melbourne, VIC 3002 Australia; 4 Melbourne School of Population and Global Health, University of Melbourne, Melbourne, VIC 3010 Australia; 5 State Key Laboratory of Ophthalmology, Zhongshan Ophthalmic Center, Sun Yat-sen University, Guangzhou, 510060 China; 6 Monash e-Research Center, Faculty of Engineering, Airdoc Research, Nvidia AI Technology Research Center, Monash University, Melbourne, VIC 3800 Australia

**Keywords:** Disease trajectory, Cancer, Cardiovascular disease, Hypertension, Chronic kidney disease, Multiple chronic diseases, Multimorbidity, Premature mortality

## Abstract

**Background:**

Little is known regarding life-course trajectories of important diseases. We aimed to identify diseases that were strongly associated with mortality and test temporal trajectories of these diseases before mortality.

**Methods:**

Our analysis was based on UK Biobank. Diseases were identified using questionnaires, nurses’ interviews, or inpatient data. Mortality register data were used to identify mortality up to January 2021. The association between 60 individual diseases at baseline and in the life course and incident mortality was examined using Cox proportional regression models. Those diseases with great contribution to mortality were identified and disease trajectories in life course were then derived.

**Results:**

During a median follow-up of 11.8 years, 31,373 individuals (median age at death (interquartile range): 70.7 (65.3–74.8) years, 59.4% male) died of all-cause mortality (with complete data on diagnosis date of disease), with 16,237 dying with cancer and 6702 with cardiovascular disease (CVD). We identified 37 diseases including cancers and heart diseases that were associated with an increased risk of mortality independent of other diseases (hazard ratio ranged from 1.09 to 7.77). Among those who died during follow-up, 2.2% did not have a diagnosis of any disease of interest and 90.1% were diagnosed with two or more diseases in their life course. Individuals who were diagnosed with more diseases in their life course were more likely to have longer longevity. Cancer was more likely to be diagnosed following hypertension, hypercholesterolemia, CVD, or digestive disorders and more likely to be diagnosed ahead of CVD, chronic kidney disease (CKD), or digestive disorders. CVD was more likely to be diagnosed following hypertension, hypercholesterolemia, or digestive disorders and more likely to be diagnosed ahead of cancer or CKD. Hypertension was more likely to precede other diseases, and CKD was more likely to be diagnosed as the last disease before more mortality.

**Conclusions:**

There are significant interplays between cancer and CVD for mortality. Cancer and CVD were frequently clustered with hypertension, CKD, and digestive disorders with CKD highly being diagnosed as the last disease in the life course. Our findings underline the importance of health checks among middle-aged adults for the prevention of premature mortality.

**Supplementary Information:**

The online version contains supplementary material available at 10.1186/s12916-022-02384-3.

## Background

Chronic conditions were estimated to account for 63% of 54.6 million global deaths in 2008, and this number increased to 71% of 56.7 million in 2016 [1, 2]. Cardiovascular diseases (CVDs) and cancers are the first two leading causes of mortality accounting for 31.8% and 17.1% of global deaths in 2017, respectively [3]. In the UK, 89.7% of total mortality was attributed to non-communicable diseases in 2017 with cancers, CVDs, and dementia as the first three leading contributors [4]. Of all deaths, 77.0%, 17.3%, and 5.7% were people who were aged 70 years or older, 50–69 years, and younger than 50 years old, respectively [4]. The leading cause of mortality is cancer followed by CVD among people who died younger than 70 years; however, CVD is the leading cause followed by cancer among those who died 70 years or over [4]. It has been estimated that life expectancy at birth increased rapidly until 2010, but slowly since 2010 in the UK [5]. One explanation for this is that the population is reaching the biological limits of longevity, but addressing the causes of premature mortality may help promote longevity.

Research shows that most individuals over 50 years have not one but several comorbidities (defined as multimorbidity) [6, 7], and multimorbidity is associated with an increased risk of mortality [8]. Trajectory analyses as an emerging method have been used to identify temporal disease progression patterns for predicting and preventing future diseases [9, 10]. Most people may die because of multiple diseases. A previous study based on the UK Biobank cohort has investigated the disease trajectories during follow-up and mortality among individuals with depression [11]. However, this study is limited by failing to identify temporal trajectories of important diseases in the life course.

Prevention and reduction of premature mortality risk are of paramount importance for achieving further substantial life expectancy increases. Using the UK Biobank, we aimed to examine the association between important diseases and incident mortality. Based on the diseases with great contribution to mortality, disease trajectories in the life course were identified.

## Methods

### Study population

The UK Biobank is a population-based cohort study of 502,505 participants aged 40–73 years at baseline between 2006 and 2010. Participants were recruited from one of the 22 assessment centers throughout the UK [12]. The study design, recruitment flow, and population have been described in detail elsewhere [12]. A baseline assessment was conducted among 502,505 out of approximately 9.2 million people invited. Participants provided information on geographic factors, lifestyle, and other health-related aspects through comprehensive baseline questionnaires, interviews, and physical measurements.

The UK Biobank study’s ethical approval was granted by the National Information Governance Board for Health and Social Care and the NHS North West Multicentre Research Ethics Committee. All participants provided informed consent through electronic signature at baseline assessment. The present study was conducted under application number 62443 of the UK Biobank resource [13].

### Ascertainment of diseases

Diseases were defined if participants reported that they had ever been told by a doctor that they had a disease (field code for each disease is listed in Additional file 1: Table S1). A further question “What was your age when the disease was first diagnosed?” was requested to answer for those who reported a diagnosis of disease. The checks for disease diagnosis age were performed to confirm whether the diagnosis age was within the rationale range. Individuals who were uncertain about the diagnosis age provided an estimate or selected “Do not know”. Sixty major diseases including CVD, cancer, diabetes, dementia, and chronic kidney disease (CKD) were included in the analysis.

Additional disease cases at baseline and follow-up were defined using inpatient data. The Hospital Episode Statistics database, the Scottish Morbidity Record, and the Patient Episode Database were used to capture inpatient hospital records in England, Scotland, and Wales [12]. The inpatient hospital data for the UK Biobank participants were available since 1997 [12]. The codes for international classification diseases (ICD) for each of the 60 diseases are listed in Additional file 1: Table S2. The age at diagnosis of disease (years) was then computed by subtracting the birth date from the initial diagnosed date divided by 365.25. The incident cases of these 60 diseases during follow-up were identified using ICD codes.

### Ascertainment of mortality

Mortality data for participants in England and Wales were obtained from the National Health Service Digital, and the mortality data for the participants in Scotland were obtained from the National Health Service Central Register [12]. Specific causes of mortality with a primary diagnosis were identified using ICD codes [14]. Person-years were calculated from the date of baseline assessment (2006–2010) to the date of death, or the end of follow-up (31 December 2020 for England/Wales and 18 January 2021 for Scotland), whichever came first.

### Covariates

Data on age, gender, ethnicity, education, and income were collected using a touch-screen computer. A detailed questionnaire on lifestyle factors, including diet, physical activity, smoking status, and frequency of alcohol consumption was also completed. We divided sleep duration into three groups: <7, 7–9, and >9 h [15]. An excess metabolic equivalent (MET)-hours/week of physical activity during work and leisure time was estimated using questions that were similar to those used in the short form of the International Physical Activity Questionnaire [16]. A healthy diet score was computed based on seven commonly eaten food groups following recommendations on dietary priorities for cardiometabolic health [17] with a higher score representing a healthier diet. In the present analysis, the high diet quality was defined as the diet score≥4. A genetic risk score (GRS) for longevity was computed using 78 single-nucleotide polymorphisms [18].

BMI was calculated as measured weight in kilograms divided by measured height in meters squared. Glycated hemoglobin (HbA1c) was measured using high-performance liquid chromatography on a Bio-Rad Variant II Turbo. Total cholesterol, high-density lipoprotein cholesterol (HDL-C), low-density lipoprotein cholesterol (LDL-C), and triglycerides were measured by direct enzymatic methods (Konelab, Thermo Fisher Scientific, Waltham, Massachusetts).

### Statistical analysis

Cox proportional hazard regression models were used to examine the association between each of the 60 major diseases at baseline (including both self-reported and inpatient data) and incident mortality. The covariates were selected based on clinical knowledge and potential multicollinearity was tested in the analysis. Those covariates with a variance inflation factor greater than 5 were excluded from the analysis (LDL-C and total cholesterol). Model 2 was adjusted for age, gender, ethnicity, education, income, BMI, smoking, physical acidity, alcohol consumption, sleep duration, diet, blood pressure, longevity GRS, HDL-C, triglycerides, and HbA1c. For the analysis of each disease, the effect of the other diseases was additionally adjusted for in the full model (Model 3). All the individual diseases were found to have no potential multicollinearity (all individual variance inflation factors<2). Individuals who died within the first year of follow-up were excluded from the analysis. The log-minus-log plots were used to test the proportional hazards assumption. We then examined the association between each major disease was diagnosed in the life course (including those reported at baseline and follow-up) and mortality using Cox proportional hazard regression models. Individuals with a disease diagnosed in the last year before mortality were excluded from the analysis. Benjamin-Hochberg’s procedure was used to control the false discovery rate at a 5% level for multiple comparisons [19].

We then classified the diseases that were significantly associated with mortality into groups. Individuals who died during follow-up were divided into groups according to the number of types of diseases: 0, 1, 2, 3, 4, 5, and ≥6. Baseline data were expressed as means ± standard deviations, medians (interquartile ranges [IQRs]), or frequency (percentage) according to the number of types of diseases. ANOVA analysis for normally distributed continuous variables, Wilcoxon rank-sum test for skewed continuous variables, and chi-square test for categorical variables were used to examine the difference in baseline characteristics across the number of diseases in the life course.

Temporal disease trajectories were identified using the permutation of diseases (multiple diseases in order of age at diagnosis) among individuals who were diagnosed with two or more diseases before mortality. The age at diagnosis of diseases was based on self-reported or inpatient data. For example, among those who were diagnosed with two diseases in the life course, the primary disease was defined as the disease diagnosed at a younger age and the secondary disease as the disease diagnosed at an older age. This analysis was also conducted for those with 3, 4, 5, and ≥6 diseases separately. The disease trajectories were identified for all mortality, cancer mortality, and CVD mortality, separately.

For chronic diseases at baseline that were associated with a lower risk of incident mortality, a matched analysis was conducted to test these associations with controls matched by age and gender. For each individual with the disease, one control was randomly selected from those free of the corresponding disease.

The percentage of participants with missing values in physical activity, household income, education, BMI, alcohol consumption, smoking, sleep duration, LDL-C, triglycerides, blood pressure, and HbA1c was 19.9%, 15.3%, 2.0%, 2.0%, 0.3%, 0.6%, 0.8%, 14.4%, 6.6%, 6.0%, and 7.2%, respectively. Missing values for categorical variables were assigned as a single category. Missing values for continuous covariates were assigned as the mean. Sensitivity analysis for associations between individual diseases and mortality was conducted among participants with complete data.

All data analyses were conducted using SAS 9.4 (SAS Institute Inc.), and P values were two-sided with statistical significance set at <0.05.

## Results

### Incidence of mortality

During a median follow-up of 11.8 years (IQR 11.1–12.6), 33,393 individuals died of all-cause mortality (overall incidence: 6.6%), and their longevity ranged from 40.3 to 83.7 years (69.5 ± 7.4). Around 97.0% of them were defined as premature mortality with the age at death younger than the life expectancy at birth in the UK (83.1 years for women, 79.4 years for men).

### Disease and incident mortality

After excluding individuals who died within the first year of follow-up (*n*=394), 502,131 participants were included in the analysis. There was no violation of the proportional hazards assumption for most diseases examined (Additional file 2: Figures S1-S5).

**Fig. 1:**
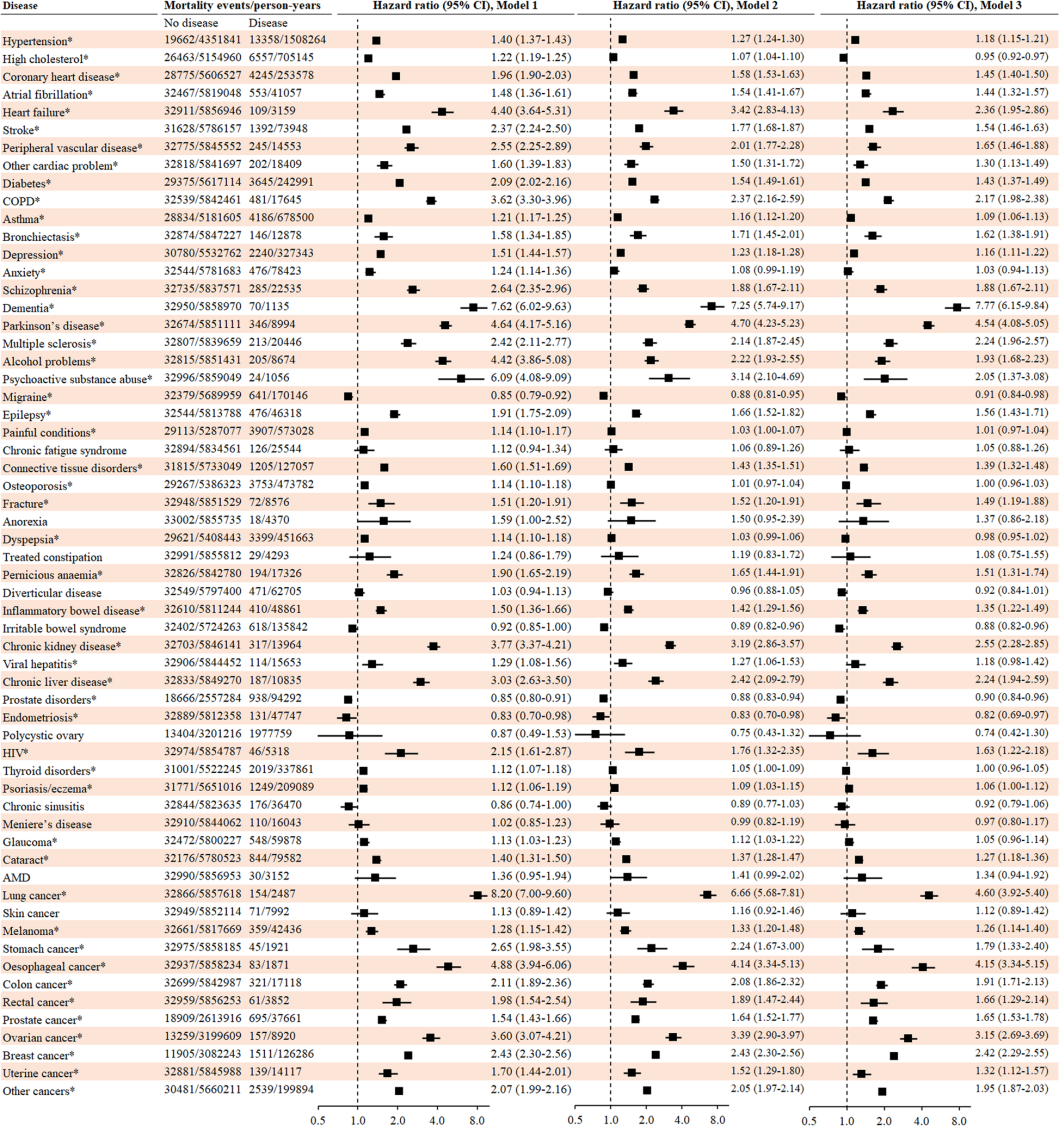
Risk for mortality associated with individual diseases of interest at baseline. Cox proportional hazard regression models were used to examine the association between each of the 60 major diseases at baseline and incident mortality. Model 1 was adjusted for age and gender; Model 2 was adjusted for Model 1 plus ethnicity, education, income, BMI, smoking, physical acidity, alcohol consumption, sleep duration, diet, blood pressure, HDL-C, triglycerides, and HbA1c. Model 3 was adjusted for Model 2 plus all other 59 chronic diseases. The analysis for breast cancer, ovarian cancer, endometriosis, and polycystic ovary was conducted among women only while the analysis for prostate cancer and prostate disorders was conducted among men only. Individuals with disease diagnosed in the last year before mortality were excluded from the analysis. Horizontal lines indicate the ranges of the 95% CIs and the vertical dash lines indicate the hazard ratio of 1.0. *Refers to significant associations after adjustment for false discovery rate at a 5% level using Benjamin-Hochberg’s procedure

As shown in Fig. 1, 50 out of 60 diseases were significantly associated with incident mortality after adjustment for false discovery rate. After adjustment for geographic factors, lifestyle factors, and biomarkers, the number of diseases that were significantly associated with mortality was reduced to 43. This number was reduced to 37 when all other diseases were adjusted for in the full model. The multivariable-adjusted hazard ratio (HR) ranged from 0.87 to 7.77. The highest HRs (95% confidence interval [CI]) for diseases that were positively associated with incident mortality were 7.77 (6.15–9.84) for dementia, 4.60 (3.92–5.40) for lung cancer, 4.54 (4.08–5.05) for Parkinson’s disease, 4.15 (3.34–5.15) for oesophageal cancer, and 3.15 (2. 69–3.69) for ovarian cancer.

Three diseases that were inversely associated with incident mortality included migraine (HR (95% CI): 0.91 (0.84–0.98)), endometriosis (0.82 (0.69–0.97)), and prostate disorder (excluded prostate cancer, 0.90 (0.84–0.96)). As Additional file 1: Table S3 shows, individuals with migraine, endometriosis, or prostate disorder did not differ in the incidence of mortality compared with age-/gender-matched controls in the full model.

When analyzing diseases in the life course, 55 out of 60 diseases were significantly associated with incident mortality after adjustment for false discovery rate. In the multivariable analysis, the highest HRs (95% CIs) for diseases that were significantly associated with incident mortality were 3.76 (3.48–4.07) for ovarian cancer, 3.22 (3.14–3.31) for other cancer, 2.98 (2.84–3.13) for lung cancer, 2.37 (2.17–2.59) for oesophageal cancer, and 2.20 (2.06–2.35) for Parkinson’s disease (Additional file 3: Figure S6).

Diseases that were significantly associated with premature mortality were classified into 16 groups for those with a contribution of 0.5% or more (Additional file 1: Table S4).

A larger number of the 16 groups of diseases was associated with an increased risk of mortality during follow-up (Additional file 1: Table S7).

### Baseline characteristics

Among 33,393 participants who died during follow-up, 2020 with missing values on age at diagnosis of any disease of interest were excluded from the analysis. A total of 31,373 participants (median age at death (IQR): 70.7 (65.3–74.8) years, 59.4% male) were included in the disease trajectory analysis. The leading cause of mortality was cancer (*n*=16237), followed by CVD (*n*=6702). There were 2.2% (*n*=702) of deaths who did not have a diagnosis of any disease of interest using questionnaires or inpatient data before mortality. The percentages of deaths who were diagnosed with 1, 2, 3, 4, 5, or ≥6 (6–14) diseases were 7.7%, 12.8%, 15.7%, 15.8%, 14.5, and 31.3%, respectively. Individuals without being diagnosed with any disease of interest in life course were more likely to be younger and male and have higher levels of education, income, and total cholesterol, but lower levels of HbA1c compared to those with one or more diseases (Table 1). Individuals who died during follow-up were more likely to be older, male, and lowly educated. The mortality was associated with lower household income, lower diet quality, smoking, and higher levels of BMI, systolic blood pressure, triglycerides, and HbA1c (Additional file 1: Table S6).

**Table 1:** Baseline characteristics of participants across the number of diseases in life course

	Number of diseases in life course among individuals who died during follow-up							*P* value^a^
	0 (*n*=702)	1 (*n*=2406)	2 (*n*=4010)	3 (*n*=4934)	4 (*n*=4963)	5 (*n*=4545)	≥6 (*n*=9813)	
Age (years)	60 (53–65)	61 (54–65)	62 (56–66)	62 (57–66)	63 (59–67)	64 (60–67)	64 (60–67)	<0.0001
Gender								<0.0001
Female	213 (30.3)	1080 (44.9)	1833 (45.7)	2104 (42.6)	1903 (38.3)	1724 (37.9)	3718 (37.9)	
Male	489 (69.7)	1326 (55.1)	2177 (54.3)	2830 (57.4)	3060 (61.7)	2821 (62.1)	6095 (62.1)	
Ethnicity								<0.0001
Whites	676 (96.3)	2314 (96.2)	3900 (97.3)	4772 (96.7)	4782 (96.4)	4353 (95.8)	9285 (94.6)	
Non-whites	24 (3.4)	72 (3.0)	94 (2.3)	135 (2.7)	149 (3.0)	153 (3.4)	445 (4.5)	
Unknown	2 (0.3)	20 (0.8)	16 (0.4)	27 (0.5)	32 (0.6)	39 (0.9)	83 (0.8)	
Education								<0.0001
0–5 years	119 (17.0)	485 (20.2)	845 (21.1)	1216 (24.6)	1342 (27.0)	1420 (31.2)	3722 (37.9)	
6–12 years	338 (48.1)	1078 (44.8)	1889 (47.1)	2236 (45.3)	2262 (45.6)	2039 (44.9)	4141 (42.2)	
≥13 years	233 (33.2)	770 (32.0)	1185 (29.6)	1359 (27.5)	1213 (24.4)	956 (21.0)	1630 (16.6)	
Missing	12 (1.7)	73 (3.0)	91 (2.3)	123 (2.5)	146 (2.9)	130 (2.9)	320 (3.3)	
Household income (pounds)								<0.0001
<18,000	147 (20.9)	553 (23.0)	992 (24.7)	1365 (27.7)	1545 (31.1)	1543 (33.9)	4045 (41.2)	
18,000–30,999	163 (23.2)	556 (23.1)	992 (24.7)	1164 (23.6)	1204 (24.3)	1115 (24.5)	2087 (21.3)	
31,000–51,999	158 (22.5)	476 (19.8)	761 (19.0)	861 (17.5)	826 (16.6)	663 (14.6)	1037 (10.6)	
52,000–100,000	107 (15.2)	311 (12.9)	498 (12.4)	541 (11.0)	427 (8.6)	311 (6.8)	438 (4.5)	
>100,000	33 (4.7)	109 (4.5)	110 (2.7)	126 (2.6)	95 (1.9)	60 (1.3)	82 (0.8)	
Unknown	23 (3.3)	103 (4.3)	191 (4.8)	250 (5.1)	263 (5.3)	261 (5.7)	752 (7.7)	
Not answered	71 (10.1)	298 (12.4)	466 (11.6)	627 (12.7)	603 (12.1)	592 (13.0)	1372 (14.0)	
Physical activity (MET-minutes/week)	2651.9 (1050.0–3266.0)	2649.7 (1032.0 3146.0)	2532.5 (1004.0–2970.0)	2586.0 (988.5–2946.0)	2651.9 (975.0–2844.0)	2478.0 (826.5–2758.0)	2186.0 (693.0–2651.9)	<0.0001
Diet quality^b^								<0.0001
Low	292 (41.6)	1005 (41.8)	1702 (42.4)	2180 (44.2)	2189 (44.1)	2129 (46.8)	4905 (50.0)	
High	410 (58.4)	1401 (58.2)	2308 (57.6)	2754 (55.8)	2774 (55.9)	2416 (53.2)	4908 (50.0)	
Alcohol consumption								<0.0001
Never	21 (3.0)	87 (3.6)	153 (3.8)	220 (4.5)	207 (4.2)	210 (4.6)	644 (6.6)	
Previous	20 (2.8)	78 (3.2)	174 (4.3)	230 (4.7)	276 (5.6)	305 (6.7)	951 (9.7)	
Current	659 (93.9)	2234 (92.9)	3677 (91.7)	4464 (90.5)	4465 (90.0)	4003 (88.1)	8161 (83.2)	
Missing	2 (0.3)	7 (0.3)	6 (0.1)	20 (0.4)	15 (0.3)	27 (0.6)	57 (0.6)	
Smoking								<0.0001
Never	339 (48.3)	1197 (49.8)	1837 (45.8)	2091 (42.4)	1936 (39.0)	1617 (35.6)	2958 (30.1)	
Former	224 (31.9)	783 (32.5)	1486 (37.1)	1910 (38.7)	2082 (42.0)	2017 (44.4)	4554 (46.4)	
Current	135 (19.2)	411 (17.1)	666 (16.6)	890 (18.0)	910 (18.3)	866 (19.1)	2193 (22.3)	
Missing	4 (0.6)	15 (0.6)	21 (0.5)	43 (0.9)	35 (0.7)	45 (1.0)	108 (1.1)	
Sleep duration (hours)								0.0385
<7	170 (24.2)	545 (22.7)	949 (23.7)	1218 (24.7)	1249 (25.2)	1203 (26.5)	2859 (29.1)	
7–9	521 (74.2)	1792 (74.5)	2944 (73.4)	3519 (71.3)	3506 (70.6)	3106 (68.3)	6148 (62.7)	
>9	8 (1.1)	48 (2.0)	97 (2.4)	144 (2.9)	162 (3.3)	181 (4.0)	641 (6.5)	
Missing	3 (0.4)	21 (0.9)	20 (0.5)	53 (1.1)	46 (0.9)	55 (1.2)	165 (1.7)	
BMI (kg/m^2^)	26.84±4.30	26.57±4.28	26.91±4.48	27.32±4.76	27.86±5.03	28.41±5.07	29.48±5.62	<0.0001
Total cholesterol (mmol/L)	6.01±1.02	5.85±1.06	5.81±1.09	5.70±1.14	5.51±1.19	5.37±1.21	5.13±1.23	<0.0001
HDL-C (mmol/L)	1.42±0.35	1.44±0.36	1.45±0.37	1.43±0.37	1.39±0.36	1.37±0.36	1.33±0.37	<0.0001
LDL-C (mmol/L)	3.83±0.78	3.69±0.79	3.65±0.82	3.57±0.86	3.44±0.89	3.33±0.90	3.14±0.90	<0.0001
Triglycerides	1.64 (1.13–2.18)	1.59 (1.09–2.09)	1.59 (1.11–2.11)	1.62 (1.12–2.12)	1.66 (1.16–2.20)	1.75 (1.21–2.29)	1.75 (1.25–2.37)	<0.0001
HbA1c (mmol/mol)	35.51±3.90	35.73±4.99	36.12±5.44	36.80±6.85	37.74±7.83	38.81±9.54	41.56±11.74	<0.0001
DBP (mmHg)	83.9±10.8	82.3±9.6	82.3±10.0	82.7±10.3	82.6±10.3	82.8±10.2	81.6±10.6	<0.0001
SBP (mmHg)	142.7±19.6	138.5±17.8	139.5±18.7	141.1±18.9	142.2±19.0	143.4±19.4	142.8±19.6	<0.0001
Genetic risk score^c^	0.49 (0.45–0.53)	0.49 (0.46–0.53)	0.50 (0.46–0.53)	0.49 (0.46–0.53)	0.49 (0.45–0.53)	0.49 (0.46–0.53)	0.49 (0.45–0.53)	0.0435
Cause of mortality								<0.0001
Cancer	103 (14.7)	1284 (53.4)	2367 (59.0)	2965 (60.1)	2875 (57.9)	2376 (52.3)	4267 (43.5)	
CVD	318 (45.3)	560 (23.3)	776 (19.4)	921 (18.7)	927 (18.7)	985 (21.7)	2215 (22.6)	
External reason	71 (10.1)	90 (3.7)	117 (2.9)	88 (1.8)	83 (1.7)	66 (1.5)	110 (1.1)	
Others	210 (29.9)	472 (19.6)	750 (18.7)	960 (19.5)	1078 (21.7)	1118 (24.6)	3221 (32.8)	

Data are means ± standard deviations, medians (interquartile range), or N (%). *BMI* Body mass index, *CVD* Cardiovascular disease, *DBP* Diastolic blood pressure, *HbA1c* Glycated haemoglobin, *HDL-C* High-density lipoprotein cholesterol, *LDL-C* Low-density lipoprotein cholesterol, *MET* Metabolic equivalent, *SBP* Systolic blood pressure

^a^Baseline data were expressed as means ± standard deviations, medians (interquartile ranges), or frequency (percentage) according to the number of types of diseases. ANOVA analysis for normally distributed continuous variables, Wilcoxon Rank Sum Test for skewed continuous variables, and Chi-square test for categorical variables was used to examine the difference in baseline characteristics across the number of diseases in life-course.

^b^Diet score was computed based on seven commonly eaten food groups following recommendations on dietary priorities for cardiometabolic health with higher score representing healthier diet. High diet quality was defined as diet score≥4.

^c^The genetic risk score (GRS) for longevity was compuated using 78 single-nucleotide polymorphisms.

### Number of diseases and longevity

**Fig. 2:**
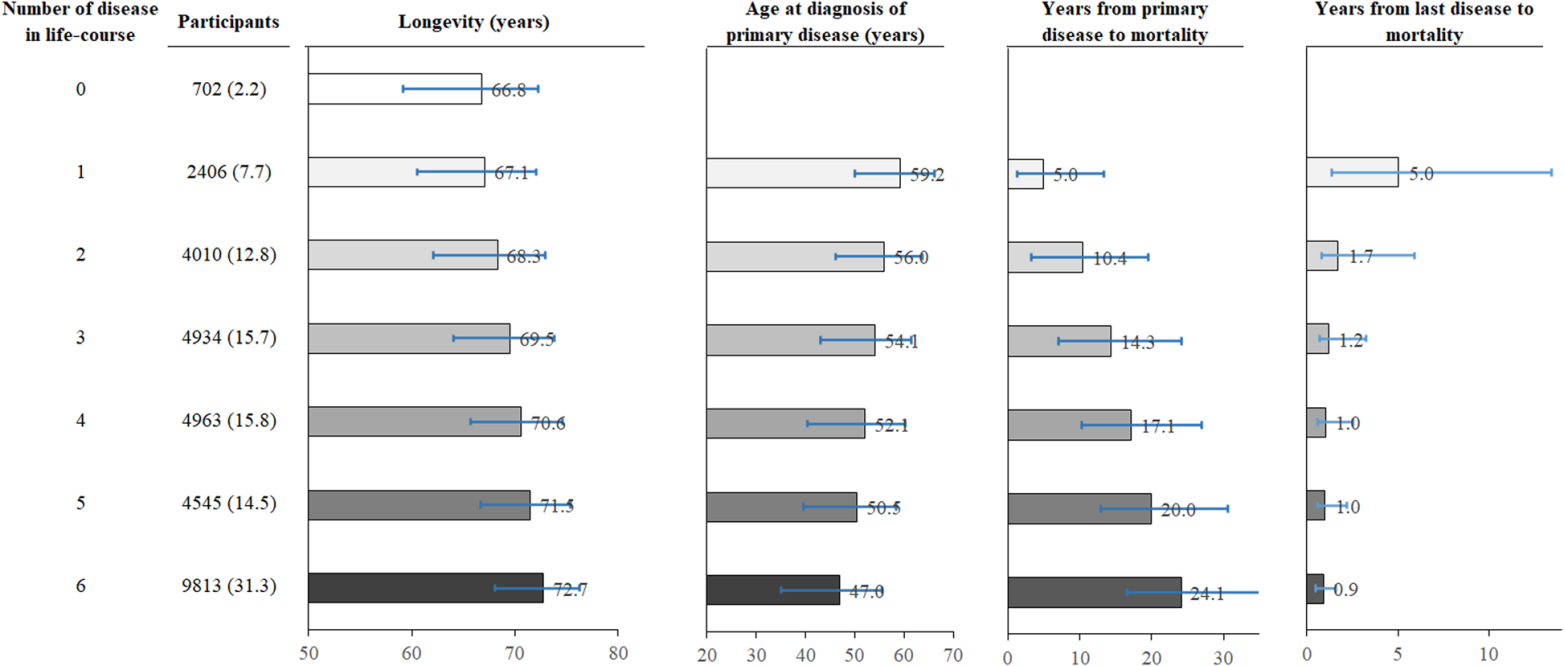
Number of diseases in life course and longevity among individuals who died from any reasons. The barchart displays the medians (interquartile ranges) for longevity, age of diagnosis of primary disease, years from primary disease to mortality, and years from last disease to mortality, respectively. Number of diseases was computed based on the16 groups of diseases accounting for 0.5% or more of all disease. Diseases diagnosed in life course were identified using self-reported questionnaire, nurses’ interview, and inpatient records. Primary disease is the first one of diseases of interest diagnosed in life course, and the last disease is the last one diagnosed before mortality. Horizontal lines indicate interquartile ranges for medians

Individuals who were diagnosed with more diseases in their life course were more likely to have longer longevity. The median time from the initial diagnosis to mortality was 5.0 years (IQR 1.3–13.4) among individuals who were diagnosed with one disease in their life course. Among individuals who were diagnosed with two diseases in the life course, the median time from initial diagnosis of the primary disease to mortality was 10.4 years (IQR 3.3–19.5) and the number for the secondary disease was 1.7 years (IQR 0.8–5.9). Among individuals who were diagnosed with three or more diseases in the life course, the median time from initial diagnosis of the primary disease to mortality ranged from 14.3 (IQR 7.1–24.1) to 24.1 years (IQR 16.6–35.8) and the number for the last disease ranged from 0.9 (IQR 0.5–1.6) to 5.0 years (IQR 1.3–13.4, Fig. 2).

### Disease trajectories in life course for all mortality

**Fig. 3:**
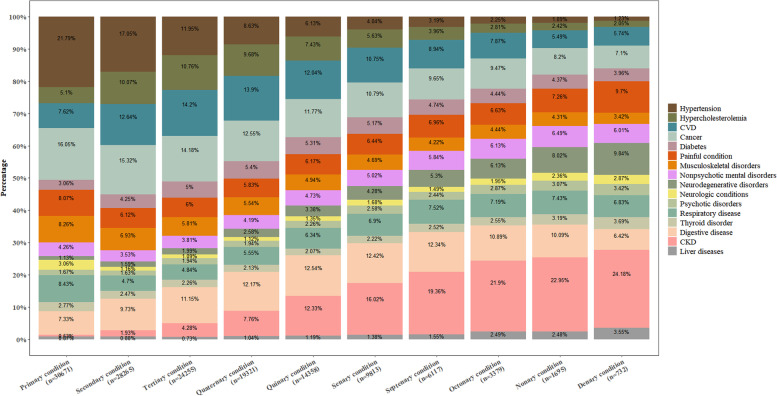
Proportion of major disease groups for the first ten diagnosed conditions in life course. The temporal trajectory was computed based the 16 groups of diseases according to the age at diagnosis of the diseases among 30,671 individuals who was diagnosed with at least one disease in life course. Primary disease is the first one of diseases of interest diagnosed in life-course. Given only 268 participants were diagnosed with 11 or more diseases in life course, the first ten diagnosed diseases are shown in this figure

As Fig. 3 shows, among individuals with at least one disease diagnosed during their life course, the most common primary condition was hypertension followed by cancer, respiratory disease, musculoskeletal disorders, and painful conditions. The proportion contributed by hypertension decreased from 21.8% of the primary condition to 1.2% of the denary condition. The number for CKD increased from 0.5 to 24.2%. An increasing trend in the proportion of neurodegenerative disorders and liver diseases and a decreasing trend in the proportion of cancer was seen with the accumulation of disease.

Among individuals who were diagnosed with one disease only in the life course, cancer, hypertension, and CVD were the three leading contributors with the proportion as 50.9%, 10.4%, and 7.6%, respectively (Additional file 1: Table S7).

Among individuals who were diagnosed with two diseases in the life course, the proportion of cancer increased from 28.5% of the primary disease to 32.2% of the secondary disease. Around 29.8% of individuals with cancer as the primary disease was diagnosed with digestive disorders as the secondary disease. Around 22.9% and 18.4% of cancer as the secondary disease was diagnosed following hypertension or digestive disorders, respectively. Around 29.8% and 29.2% of CVD as the secondary disease was diagnosed following hypertension or cancer, respectively (Additional file 3: Figure S7).

Among individuals who were diagnosed with three diseases in the life course, the proportion of hypertension decreased from 22.6% of the primary disease to 5.4% of the tertiary disease. Whilst the proportion of CKD increased from 0.5% of the primary disease to 10.4% of the tertiary disease. A large proportion of CVD as the secondary disease was diagnosed following hypertension (33.6%) or cancer (16.9%) as the primary disease. A large proportion of CVD as the tertiary disease was diagnosed following cancer (25.0%), hypertension (19.2%), or digestive disorders (10.1%) as the secondary disease. A large proportion of cancer as the secondary disease was diagnosed following hypertension (49.5%) or digestive disorders (26.8%) as the primary disease. The proportion of cancer as the tertiary disease was diagnosed following digestive disorders or hypertension as the secondary disease was 19.5% or 17.9%, respectively (Additional file 3: Figure S8).

Among individuals who were diagnosed with four diseases in the life course, the proportion of hypertension decreased from 25.9% of primary disease to 4.3% of the quaternary disease, whilst the number for CKD increased from 0.6 to 16.3%. CVD was more likely to be diagnosed following hypertension, hypercholesterolemia, cancer, or digestive disorders and more likely to be diagnosed ahead of cancer or CKD. Cancer was more likely to be diagnosed after hypertension, hypercholesterolemia, CVD, or digestive disorders and more likely to be diagnosed ahead of CVD, CKD, or digestive disorders (Additional file 3: Figure S9).

Similar results were observed for individuals who were diagnosed with five (Additional file 3: Figure S10) or six or more diseases in the life course (Additional file 3: Figure S11).

### Disease trajectories in life course for cancer mortality

Among individuals who died with cancer, 0.6% did not have a diagnosis of any disease of interest in the life course. Around 7.5% of them had one disease only diagnosed in the life course and cancer contributed to 89.9% of the disease (Additional file 1: Table S7). Among individuals who were diagnosed with two diseases before dying from cancer, 43.5% of the primary disease was contributed to cancer and 51.3% of the secondary disease was contributed to cancer. A large proportion of individuals with cancer as the primary disease was diagnosed with digestive disorders (31.7%), CKD (13.1%), or CVD (11.2%) as the secondary disease (Additional file 4: Figure S12). Cancer contributed to 25.3% of the primary disease, 36.2% of the secondary disease, and 35.4% of the tertiary disease. Cancer is more likely to be diagnosed ahead of hypertension, CVD, digestive disorders, or CKD and to be diagnosed following hypertension, digestive disorders, or CVD (Additional file 4: Figure S13). Similar results were seen for individuals who were diagnosed with four (Additional file 4: Figure S14), five (Additional file 4: Figure S15), or six or more diseases (Additional file 4: Figure S16) before dying from cancer. Hypertension was more likely to precede other diseases, and CKD was more likely to be diagnosed following other diseases.

### Disease trajectories in life course for CVD mortality

Among individuals who died with CVD, 4.4% were not diagnosed with any disease of interest in life-course. Among 573 individuals who were diagnosed with one disease only before dying from CVD, 23.7% were diagnosed with hypertension, and 21.5% were diagnosed with CVD (Additional file 1: Table S7). Among individuals who were diagnosed with two diseases in the life course, a large proportion of the primary disease was contributed to hypertension, hypercholesterolemia, and CVD (Additional file 4: Figure S17). A large proportion of CVD was diagnosed following hypertension or hypercholesterolemia, and CVD was more likely to be diagnosed ahead of CKD before death. Around 27.8% of the last disease before mortality was CKD, largely following CVD, digestive disorders, cancer, hypertension, or hypercholesterolemia (Additional file 4: Figures S18-S21).

### Sensitivity analysis

Among participants with complete data (covariates), 48 out of 60 diseases were significantly associated with incident mortality after adjustment for false discovery rate. Migraine, endometriosis, and glaucoma were significantly associated with mortality in the main analysis but not in the sensitivity analysis. Irritable bowel syndrome was associated with a decreased risk of mortality after adjustment for age and gender in the sensitivity analysis but not in the main analysis. In the multivariable analysis, an inverse association between irritable bowel syndrome and mortality was observed in both sensitivity and main analyses. All other diseases (*n*=47) were overlapped in both analyses (Additional file 1: Table S8).

## Discussion

We found that more than half of the participants died with cancer and more than one fifth died with CVD. More than 90% of the individuals were diagnosed with two or more diseases of interest in the life course. A larger number of diseases diagnosed in the life course was associated with longer longevity. Hypertension was more likely to be diagnosed ahead of CVD and cancer, whilst CKD was more likely to be diagnosed following CVD and cancer. This trend was more pronounced with the increasing number of diseases diagnosed in the life course. There were significant interplays between cancer and CVD. Similar results were found for individuals who died with cancer or CVD.

Our analysis is consistent with a previous study showing that cancer, CVD, diabetes, neurological disorders, mental disorders, chronic respiratory diseases, and digestive diseases play an important role in the development of mortality [3]. An increasing number of studies have linked cataract to increased mortality risk [20–22]. This is in line with our study demonstrating that cataract was associated with an increased risk of mortality independent of geographic factors, lifestyle, biomarkers, and other chronic diseases. A recent meta-analysis showed that findings for the association between glaucoma and mortality remained inconsistent between previous studies [22]. In our analysis, glaucoma was not significantly associated with mortality risk after adjustment of other diseases suggesting the potential risk of glaucoma was dependent on its association with other diseases. We also observed endometriosis, prostate, and migraine at baseline were associated with a lower risk of mortality, but these associations were attenuated to be non-significant in the matched analysis. A prospective cohort study of Finnish women (49,956 with endometriosis, 98,824 age- and municipality-matched controls) with a median follow-up of 17 years reported that endometriosis diagnosed by surgery was associated with a lower risk of all-cause mortality (HR (95% CI): 0.73 (0.69–0.77)) [23]. We found endometriosis (defined by both self-reported and inpatient data) was associated with a decreased risk of mortality, but this association was not significant in the age-matched analysis. The inconsistent results between the previous study and our analysis may be due to different methods used for the diagnosis of endometriosis. A recent large prospective study of 27,844 women with a median follow-up of 22.7 years showed that migraine was not significantly associated with all-cause mortality (HR (95% CI) 0.96 (0.89–1.04)) [24]. We found migraine was associated with a reduced risk of mortality, but this association was attenuated to be non-significant after adjustment for other chronic diseases. This suggests the potential beneficial effects of migraine on mortality prevention may be due to confounding. An analysis based on the Oxford Record Linkage Study and English national data demonstrated that benign prostatic hyperplasia was associated with a lower risk of mortality although the effect size was minimal [25]. Likely, we found prostate disorders (excluding prostate cancer) were associated with decreased risk of mortality, but this association was not significant after adjustment for other diseases.

There was a small proportion of individuals who were not diagnosed with any disease in the life course, a large proportion of whom died with CVD or neurogenerative/mental disorders. Although a relatively larger proportion of these individuals died from external reasons (10.1%), they had shorter longevity compared to those who were diagnosed with one or more diseases in life course even when those who died from external reasons were excluded from the analysis. As the reduction in longevity was possibly due to the unawareness of diseases in those individuals, it is imperative to screen diseases, especially CVD and neurogenerative/mental disorders among middle-aged adults. Higher total cholesterol but lower HbA1c was observed in this subgroup of individuals. As further analysis showed that individuals with fewer diseases were more likely to have fewer deadly diseases (Additional file 1: Table S9), the shorter longevity among individuals who were diagnosed with fewer diseases may be due to the fact that they were less likely to seek health check and care. Therefore, health screening is important among these participants in their mid-life.

Cancer is the leading cause of mortality and is also the most prevalent one of diseases of interest (60.2%) in the life course among individuals who died prematurely. This is consistent with a previous study demonstrating that cancer was the leading cause of life years lost and life years lost due to cancer increased by 16% from 1995 to 2015 [26]. Around one quarter of those who were diagnosed with cancer had no other disease diagnosed before the diagnosis of cancer in the life course and others had at least one disease (including hypertension, digestive disorders, or painful conditions) diagnosed before the diagnosis of cancer. Likely, previous prospective studies have shown that hypertension was associated with an increased risk of cancer [27, 28]. Digestive disorders including anorexia may increase the risk of cancer [29–31]. We found CVD was the second leading cause of premature mortality. Although CVD contributed to a much smaller proportion of premature mortality in our study, a larger proportion of mortality caused by CVD (80%) was related to modifiable risk factors compared with that caused by cancer (47%) [32]. It is well known that hypertension and hypercholesterolemia are primary causes of CVD [33], whilst blood pressure and cholesterol-lowering is shown to be beneficial for the prevention of CVD [34, 35]. There were significant interplays between cancer and CVD [36], which might explain why a large proportion of CVD was diagnosed following cancer. This is in line with previous studies showing that cancer clustered with hypertension, CVD, and/or digestive disorders is a common multimorbidity pattern in the European populations [37–40]. However, the temporal trajectories of these conditions in life course need to be investigated in more prospective cohort studies.

A systematic analysis for the Global Burden of Disease Study showed that hypertension is the leading contributor to global mortality [41]. We found, although hypertension is not the leading cause of mortality, hypertension is the most prevalent one of diseases of interest. The association between hypertension and mortality is probably attributed to the fact that a large proportion of cancer and CVD (leading causes of mortality) was diagnosed following hypertension. Several recent studies have shown that the cluster of hypertension and/or CVD and CKD is a frequently seen multimorbidity pattern [9, 37, 42]. Hypertension is more likely to precede other conditions before mortality whilst CKD is more likely to occur following other conditions [43, 44]. This suggests the importance of screening more severe conditions such as cancer and CVD among those with hypertension and the prevention of CKD also deserves scrutiny among those with one or more existing diseases. Given the interactions between various shared risk factors and the known significant hormonal shifts across this period, it is clear that longitudinal research spanning the prodrome of disease development is central to improving our understanding of the evolution of multimorbidity in the life course. It is also important to identify time windows for potential risk and preventative factors that may contribute to premature mortality.

To our knowledge, this is the first study to examine the disease trajectory in the life course based on a large population cohort. There are several potential limitations in our study. Firstly, most cases of many chronic conditions at baseline including hypertension, high cholesterol, stroke, asthma, depression, and anxiety were captured by self-reported data (Additional file 1: Table S10), whilst those conditions diagnosed during follow-up were largely captured by inpatient data given that self-reported data during follow-up were available in only a small subgroup of the UK Biobank cohort. Data on age at diagnosis of disease until recruitment among some individuals (without initial diagnosis records in the inpatient data) were based on self-reported questionnaires, which might have biased the associations. Secondly, UK Biobank participants are more likely to have better general health (lower prevalence of main chronic diseases and unhealthy behaviors). However, a previous study has demonstrated that findings regarding exposure-disease relationships may be generalized to other populations [45]. Thirdly, we present the disease trajectories for the first six and the last diseases among individuals who were diagnosed with seven or more diseases in their life course. The permutations of diseases diagnosed between the seventh and the last diseases were not displayed given the too large metrics. Finally, the severity of the diseases cannot be captured in the data and thus was not included in the analysis. This might have biased the associations as a disease of being a different severity may result in different risks for mortality or different combinations of diseases then leading to different risks of mortality.

## Conclusions

Cancer is frequently diagnosed following hypertension, or digestive disorders, and is more likely to be diagnosed ahead of CVD, CKD, or digestive disorders. Whilst CVDs are more likely to be diagnosed following hypertension, cancer, or digestive disorders and more likely to be diagnosed ahead of cancer or CKD in the life course. Hypertension tends to precede other diseases and CKD tends to follow other diseases before mortality. Our findings also underline the importance of health check for the prevention of premature mortality given no diagnosed disease was identified before mortality in some people.

## Supplementary Information

**Additional file 1: Table S1.** Field codes for diseases of interest. **Table S2.** ICD codes for diseases of interest. **Table S3.** Matching analysis for diseases that were inversely associated with incident mortality. **Table S4.** Classification of diseases. **Table S5.** Death and the number of diseases. **Table S6.** Baseline characteristics of participants by mortality. **Table S7.** Disease contribution among individuals who were diagnosed with one disease only in life-course. **Table S8.** Risk for mortality associated with individual diseases of interest at baseline among individuals with complete data. **Table S9.** Prevalence of individual diseases by a number of diseases in life-course. **Table S10.** Number of events at baseline captured by self-reported and inpatient data.

**Additional file 2: Figure S1.** Proportional hazards assumption test for cardiometabolic disorders and incident mortality. **Figure S2.** Proportional hazards assumption test for cardiometabolic disorders and incident mortality. **Figure S3.** Proportional hazards assumption test for cancers and incident mortality. **Figure S4.** Proportional hazards assumption test for musculoskeletal disorders, digestive disorders and incident mortality. **Figure S5.** Proportional hazards assumption test for other diseases and incident mortality.

**Additional file 3: Figure S6.** Risk for mortality associated with individual diseases of interest in life-course. **Figure S7.** Disease trajectory in life-course among individuals who were diagnosed with two diseases before dying from any reasons. **Figure S8.** Disease trajectory in life-course among individuals who were diagnosed with three diseases before dying from any reasons. **Figure S9.** Disease trajectory in life-course among individuals who were diagnosed with four diseases before dying from any reasons. **Figure S10.** Disease trajectory in life-course among individuals who were diagnosed with five diseases before dying from any reasons. **Figure S11.** Disease trajectory in life-course among individuals who were diagnosed with six or more diseases before dying from any reasons.

**Additional file 4: Figure S12.** Disease trajectory in the whole life-course among individuals who were diagnosed with two diseases before dying from cancer. **Figure S13.** Disease trajectory in the whole life-course among individuals who were diagnosed with three diseases before dying from cancer. **Figure S14.** Disease trajectory in the whole life-course among individuals who were diagnosed with four diseases before dying from cancer. **Figure S15.** Disease trajectory in the whole life-course among individuals who were diagnosed with five diseases before dying from cancer. **Figure S16.** Disease trajectory in the whole life-course among individuals who were diagnosed with six or more diseases before dying from cancer. **Figure S17.** Disease trajectory in the whole life-course among individuals who were diagnosed with two diseases before dying from cardiovascular disease. **Figure S18.** Disease trajectory in the whole life-course among individuals who were diagnosed with three diseases before dying from cardiovascular disease. **Figure S19.** Disease trajectory in the whole life-course among individuals who were diagnosed with four diseases before dying from cardiovascular disease. **Figure S20.** Disease trajectory in the whole life-course among individuals who were diagnosed with five diseases before dying from cardiovascular disease. **Figure S21.** Disease trajectory in the whole life-course among individuals who were diagnosed with six or more diseases before dying from cardiovascular disease.

## Data Availability

Data are available in a public, open access repository (https://www.ukbiobank.ac.uk/).

## Abbreviations

BMI: Body mass index
CKD: Chronic kidney disease
CVD: Cardiovascular disease
HbA1c: Glycated hemoglobin
HDL-C: High-density lipoprotein cholesterol
HR: Hazard ratio
ICD: International classification diseases
IQR: Interquartile range
LDL-C: Low-density lipoprotein cholesterol
MET: Metabolic equivalent
SD: Standard deviation
