# Supplementary material for: Temporal trajectories of important diseases in the life course and premature mortality in the UK Biobank
Source: BMC Med. 2022 May 27;20:185. doi: 10.1186/s12916-022-02384-3 (PMC9137080; doi:10.1186/s12916-022-02384-3)
Supplement: Supplementary file 1 — Additional file 1: Table S1. Field codes for diseases of interest. Table S2. ICD codes for diseases of interest. Table S3. Matching analysis for diseases that were inversely associated with incident mortality. Table S4. Classification of diseases. Table S5. Death and the number of diseases. Table S6. Baseline characteristics of participants by mortality. Table S7. Disease contribution among individuals who were diagnosed with one disease only in life-course. Table S8. Risk for mortality associated with individual diseases of interest at baseline among individuals with complete data. Table S9. Prevalence of individual diseases by a number of diseases in life-course. Table S10. Number of events at baseline captured by self-reported and inpatient data. [file 12916_2022_2384_MOESM1_ESM.docx]

**Additional file 1**

**Table S1. Field codes for diseases of interest**

**Table S2. ICD codes for diseases of interest**

**Table S3. Matching analysis for diseases that were inversely associated with incident mortality**

**Table S4. Classification of diseases**

**Table S5. Death and the number of diseases**

**Table S6. Baseline characteristics of participants by mortality**

**Table S7. Disease contribution among individuals who were diagnosed with one disease only in life-course**

**Table S8. Risk for mortality associated with individual diseases of interest at baseline among individuals with complete data**

**Table S9. Prevalence of individual diseases by number of diseases in life-course**

**Table S10. Number of events at baseline captured by self-reported and inpatient data**

**Table S1. Field codes for diseases of interest**

| **Long term condition grouping** | **Conditions included as reported by participants** | **Field Code** |
| --- | --- | --- |
| 1. Painful conditions | Back pain | 1534 |
|  | Joint pain | 1537 |
|  | Headaches (not migraine) | 1436 |
|  | Sciatica | 1476 |
|  | Plantar fasciitis | 1540 |
|  | Carpal tunnel syndrome | 1541 |
|  | Fibromyalgia | 1542 |
|  | Arthritis | 1538 |
|  | Shingles | 1573 |
|  | Disc problem | 1532 |
|  | Prolapsed disc/slipped disc | 1312 |
|  | Spine arthritis/spondylitis | 1311 |
|  | Ankylosing spondylitis | 1313 |
|  | Back problem | 1294 |
|  | Gout | 1466 |
|  | Cervical spondylosis | 1478 |
|  | Trigeminal neuralgia | 1523 |
|  | Disc degeneration | 1533 |
|  | Trapped nerve/compressed nerve | 1257 |
| 2. Hypertension | Hypertension | 1065 |
|  | Essential Hypertension | 1072 |
| 3. Depression | Depression | 1286 |
|  | Postnatal Depression | 1531 |
| 4. Asthma | Asthma | 1111 |
| 5. Atrial fibrillation | Atrial Fibrillation | 1471 |
| 6. Coronary heart disease | Heart attack/Myocardial Infarction | 1075 |
|  | Angina | 1074 |
| 7. Dyspepsia | Gastro-oesophageal reflux (GORD)/gastric reflux | 1138 |
|  | Oesophagitis /Barrett's oesophagus | 1139 |
|  | Gastric stomach ulcers | 1142 |
|  | Gastric erosions/gastritis | 1143 |
|  | Duodenal ulcer | 1457 |
|  | Dyspepsia/indigestion | 1510 |
|  | Hiatus hernia | 1474 |
|  | Helicobacter pylori | 1442 |
| 8. Diabetes | Diabetic nephropathy | 1607 |
|  | Diabetic neuropathy/ulcers | 1468 |
|  | Diabetes | 1220 |
|  | Type 1 diabetes | 1222 |
|  | Type 2 diabetes | 1223 |
|  | Diabetic eye disease | 1276 |
|  | Thyroid problem (not cancer) | 1224 |
| 9. Thyroid disorders | Hyperthyroidism/thyrotoxicosis | 1225 |
|  | Hypothyroidism/myxoedema | 1226 |
|  | Grave’s disease | 1522 |
|  | Thyroid goitre | 1610 |
|  | Thyroiditis | 1428 |
| 10. Connective tissue disorders | Myositis/myopathy | 1322 |
|  | Systemic Lupus Erythematosus | 1381 |
|  | Connective tissue disorder | 1373 |
|  | Sjogrens syndrome/sicca syndrome | 1382 |
|  | Dermatopolymyositis | 1383 |
|  | Scleroderma/systemic sclerosis | 1384 |
|  | Rheumatoid arthritis | 1464 |
|  | Psoriatic arthropathy | 1477 |
|  | Dermatomyositis | 1480 |
|  | Polymyositis | 1481 |
|  | Polymyalgia Rheumatica | 1377 |
|  | Malabsorption/coeliac disease | 1456 |
| 11. Chronic Obstructive Pulmonary Disease (COPD) | COPD/chronic obstructive airways disease | 1112 |
|  | Emphysema/chronic bronchitis | 1113 |
|  | Emphysema | 1472 |
| 12. Anxiety | Anxiety/panic attacks | 1287 |
|  | Nervous breakdown | 1288 |
|  | Post-traumatic stress disorder | 1469 |
|  | Obsessive compulsive disorder | 1615 |
|  | Stress | 1614 |
|  | Insomnia | 1616 |
|  | Psychological/psychiatric problem | 1243 |
| 13. Irritable bowel syndrome | Irritable bowel syndrome | 1154 |
| 14. Alcohol problems | Alcohol dependency | 1408 |
|  | Alcoholic liver disease/alcoholic cirrhosis | 1604 |
| 15. Other psychoactive substance abuse | Opioid dependency | 1409 |
|  | Other substance abuse/dependency | 1410 |
| 16. Treated constipation | Constipation | 1599 |
| 17. Stroke/Transient Ischaemic Attack (TIA) | Stroke | 1081 |
|  | TIA | 1082 |
|  | Subarachnoid haemorrhage | 1083 |
|  | Brain haemorrhage | 1086 |
|  | Ischaemic stroke | 1583 |
| 18. Chronic kidney disease | Polycystic kidney | 1427 |
|  | Diabetic nephropathy | 1607 |
|  | Renal/kidney failure | 1192 |
|  | Renal failure requiring dialysis | 1193 |
|  | Renal failure not requiring dialysis | 1194 |
|  | Kidney nephropathy | 1519 |
|  | Immunoglobulin A (IgA) nephropathy | 1520 |
| 19. Diverticular disease | Diverticular disease | 1458 |
|  | Diverticulitis | 1458 |
| 20. Peripheral vascular disease | Peripheral vascular disease | 1067 |
|  | Leg claudication/intermittent claudication | 1087 |
| 21. Heart failure | Cardiomyopathy | 1079 |
|  | Hypertrophic cardiomyopathy | 1588 |
|  | Heart failure/pulmonary oedema | 1076 |
| 22. Prostate disorders | Prostate problem (not cancer) | 1207 |
|  | Enlarged prostate | 1396 |
|  | Benign prostatic hypertrophy | 1516 |
| 23. Epilepsy | Epilepsy | 1264 |
| 24. Dementia | Dementia, Alzheimer’s disease, Cognitive impairment | 1263 |
| 25. Schizophrenia/bipolar disorder | Schizophrenia | 1289 |
|  | mania/bipolar disorder/manic depression | 1291 |
| 26. Psoriasis/eczema | Eczema/dermatitis | 1452 |
|  | Psoriasis | 1453 |
| 27. Inflammatory Bowel Disease | Inflammatory Bowel Disease | 1461 |
|  | Crohn’s disease | 1462 |
|  | Ulcerative colitis | 1463 |
| 28. Migraine | Migraine | 1265 |
| 29. Chronic sinusitis | Chronic sinusitis | 1416 |
| 30. Anorexia or bulimia | Anorexia/bulimia/other eating disorder | 1470 |
| 31. Bronchiectasis | Bronchiectasis | 1114 |
| 32. Parkinson’s disease | Parkinson’s disease | 1262 |
| 33. Multiple Sclerosis | Multiple Sclerosis | 1261 |
| 34. Viral Hepatitis | Infective/viral hepatitis | 1156 |
|  | Hepatitis B | 1579 |
|  | Hepatitis C | 1580 |
|  | Hepatitis D | 1581 |
|  | Hepatitis E | 1582 |
| 35. Chronic liver disease | Oesophageal varices | 1141 |
|  | Non infective hepatitis | 1157 |
|  | Liver failure/cirrhosis | 1158 |
|  | Primary biliary cirrhosis | 1506 |
| 36. Osteoporosis | Osteoporosis | 1465 |
| 37. Chronic fatigue syndrome | Chronic fatigue syndrome | 1482 |
| 38. Endometriosis | Endometriosis | 1402 |
| 39. Meniere’s disease | Meniere’s disease | 1421 |
| 40. Pernicious Anaemia | Pernicious Anaemia | 1331 |
| 41. Polycystic ovary | Polycystic ovary | 1350 |
| 42. High cholesterol | High cholesterol | 1473 |
| 43. Heart/cardiac problem | Heart/cardiac problem | 1066 |
| 44. Fracture | fracture pelvis | 1647 |
|  | fracture neck of femur / hip | 1648 |
|  | fracture patella / knee | 1650 |
| 45. HIV | HIV/AIDS | 1439 |
| 46. Glaucoma | Glaucoma | 1277 |
| 47. Cataract | Cataract | 1278 |
| 48. AMD | AMD | 1528 |
| 49. Lung Cancer | Lung Cancer | 1001 |
| 50. Skin Cancer | Non-melanoma Cancer | 1060 |
| 51. Melanoma | Melanoma | 1059 |
| 52. Stomach Cancer | Stomach cancer | 1018 |
| 53. Oesophageal cancer | Lung Cancer | 1017 |
| 54. Colon cancer | Colon cancer | 1022 |
| 55. Rectal cancer | Rectal_cancer | 1023 |
| 56. Prostate cancer | Prostate cancer | 1044 |
| 57. Ovarian cancer | Ovarian cancer | 1039 |
| 58. Breast cancer | Breast cancer | 1002 |
| 59. Uterine/endometrial cancer | Uterine/endometrial cancer | 1040 |
| 60. other cancers | cancer of lip/mouth/pharynx/oral cavity | 1004 |
|  | salivary gland cancer | 1005 |
|  | larynx/throat cancer | 1006 |
|  | nasal cavity cancer | 1007 |
|  | ear cancer | 1008 |
|  | sinus cancer | 1009 |
|  | lip cancer | 1010 |
|  | tongue cancer | 1011 |
|  | gum cancer | 1012 |
|  | parotid gland cancer | 1015 |
|  | other salivary gland cancer | 1016 |
|  | small intestine/small bowel cancer | 1019 |
|  | large bowel cancer/colorectal cancer | 1020 |
|  | anal cancer | 1021 |
|  | liver/hepatocellular cancer | 1024 |
|  | gallbladder/bile duct cancer | 1025 |
|  | pancreas cancer | 1026 |
|  | small cell lung cancer | 1027 |
|  | non-small cell lung cancer | 1028 |
|  | peripheral nerve/autonomic nerve cancer | 1029 |
|  | eye and/or adnexal cancer | 1030 |
|  | meningeal cancer / malignant meningioma | 1031 |
|  | brain cancer / primary malignant brain tumour | 1032 |
|  | spinal cord or cranial nerve cancer | 1033 |
|  | kidney/renal cell cancer | 1034 |
|  | bladder cancer | 1035 |
|  | other cancer of urinary tract | 1036 |
|  | female genital tract cancer | 1037 |
|  | male genital tract cancer | 1038 |
|  | cervical cancer | 1041 |
|  | vaginal cancer | 1042 |
|  | vulval cancer | 1043 |
|  | testicular cancer | 1045 |
|  | penis cancer | 1046 |
|  | lymphoma | 1047 |
|  | leukaemia | 1048 |
|  | multiple myeloma | 1050 |
|  | myelofibrosis or myelodysplasia | 1051 |
|  | hodgkins lymphoma / hodgkins disease | 1052 |
|  | non-hodgkins lymphoma | 1053 |
|  | chronic lymphocytic | 1055 |
|  | chronic myeloid | 1056 |
|  | other haematological malignancy | 1058 |
|  | basal cell carcinoma | 1061 |
|  | squamous cell carcinoma | 1062 |
|  | primary bone cancer | 1063 |
|  | mesothelioma | 1064 |
|  | thyroid cancer | 1065 |
|  | parathyroid cancer | 1066 |
|  | adrenal cancer | 1067 |
|  | sarcoma/fibrosarcoma | 1068 |
|  | malignant lymph node, unspecified | 1070 |
|  | metastatic cancer (unknown primary) | 1071 |
|  | cin/pre-cancer cells cervix | 1072 |
|  | rodent ulcer | 1073 |
|  | acute myeloid leukaemia | 1074 |
|  | retinoblastoma | 1075 |
|  | kaposis sarcoma | 1076 |
|  | mouth cancer | 1077 |
|  | tonsil cancer | 1078 |
|  | oropharynx / oropharyngeal cancer | 1079 |
|  | trachea cancer | 1080 |
|  | thymus cancer / malignant thymoma | 1081 |
|  | heart / mediastinum cancer | 1082 |
|  | respiratory / intrathoracic cancer | 1084 |
|  | bone metastases / bony secondaries | 1085 |
|  | appendix cancer | 1086 |
|  | fallopian tube cancer | 1087 |
|  | malignant insulinoma | 1088 |

**Table S2. ICD codes for diseases of interest**

| **Long term condition grouping** | **Conditions included as reported by participants** | **ICD10 code** | **ICD9 code** |
| --- | --- | --- | --- |
| 1. Painful conditions | Back pain | M545 | 7245 |
|  | Joint pain | M255 | 7194 |
|  | Other headache syndromes | G44 | 339 |
|  | Headaches (not migraine) | O294 |  |
|  | Spinal and epidural anaesthesia-induced headache during labour and delivery | O745 |  |
|  | Spinal and epidural anaesthesia-induced headache during the puerperium | O894 |  |
|  | Headache | R51 |  |
|  | Sciatica | M543 | 7243 |
|  | Plantar fasciitis | M722 | 7287 |
|  | Carpal tunnel syndrome | G560 | 3540 |
|  | Fibromyalgia | M797 | 7291 |
|  | Shingles | B02 | 539 |
|  | Disc problem | M995 | 7229 |
|  | Spine arthritis/spondylitis | M4910 |  |
|  | Spine arthritis/spondylitis | M4920 |  |
|  | Ankylosing spondylitis | M45X9 | 7200 |
|  | Idiopathic gout | M100 | 274 |
|  | Lead-induced gout | M101 |  |
|  | Drug-induced gout | M102 |  |
|  | Gout due to impairment of renal function | M103 |  |
|  | Other spondylosis with myelopathy (Cervical region) | M4712 | 7211 |
|  | Other spondylosis (Cervical region) | M4782 |  |
|  | Spondylosis, unspecified (Cervical region) | M4792 |  |
|  | Trigeminal neuralgia | G500 | 3501 |
|  | Cervical disk disorders | M50 | 722 |
|  | Other intervertebral disk disorders | M51 |  |
|  | Lumbar and other intervertebral disk disorders with myelopathy | M510 |  |
|  | Lumbar and other intervertebral disk disorders with radiculopathy | M511 |  |
|  | Intervertebral disc disorders w radiculopathy, lumbar region | M5116 |  |
|  | Lumbar and other intervertebral disc disorders with radiculopathy; site unspecified | M5119 |  |
|  | Other specified intervertebral disk displacement | M512 |  |
|  | Other specified intervertebral disk degeneration | M513 |  |
|  | Other intervertebral disc degeneration, lumbosacral region | M5137 |  |
| 2. Hypertension | Essential (primary) hypertension | I10 | 401 |
|  | Hypertensive heart disease | I11 | 402 |
|  | Hypertensive heart disease with (congestive) heart failure | I110 | 403 |
|  | Hypertensive heart disease without (congestive) heart failure | I119 | 404 |
|  | Hypertensive renal disease | I12 | 405 |
|  | Hypertensive renal disease with renal failure | I120 |  |
|  | Hypertensive renal disease without renal failure | I129 |  |
|  | Hypertensive heart and renal disease | I13 |  |
|  | Hypertensive heart and renal disease with (congestive) heart failure | I130 |  |
|  | Hypertensive heart and renal disease with renal failure | I131 |  |
|  | Hypertensive heart and renal disease with both (congestive) heart failure and renal failure | I132 |  |
|  | Hypertensive heart and renal disease, unspecified | I139 |  |
|  | Secondary hypertension | I15 |  |
|  | Renovascular hypertension | I150 |  |
|  | Hypertension secondary to other renal disorders | I151 |  |
|  | Hypertension secondary to endocrine disorders | I152 |  |
|  | Other secondary hypertension | I158 |  |
|  | Secondary hypertension, unspecified | I159 |  |
| 3. Depression | Depressive episode | F32 | 2962 |
|  | Recurrent depressive disorder | F33 | 2963 |
|  | Dysthymia | F341 | 3004 |
|  | Other recurrent mood [affective] disorders | F381 | 311 |
|  | Postschizophrenic depression | F204 |  |
| 4. Asthma | Asthma | J45 | 493 |
|  | Predominantly allergic asthma | J450 |  |
|  | Nonallergic asthma | J451 |  |
|  | Mixed asthma | J458 |  |
|  | Asthma, unspecified | J459 |  |
| 5. Atrial Fibrillation | Atrial fibrillation and flutter | I48 | 427 |
|  | Paroxysmal atrial fibrillation | I480 |  |
|  | Persistent atrial fibrillation | I481 |  |
|  | Chronic atrial fibrillation | I482 |  |
|  | Atrial fibrillation and atrial flutter, unspecified | I489 |  |
| 6. Coronary Heart Disease | Angina pectoris | I20 | 413 |
|  | Acute myocardial infarction | I21 | 410 |
|  | Subsequent myocardial infarction | I22 | 411 |
|  | Certain current complications following acute myocardial infarction | I23 | 412 |
|  | Other acute ischaemic heart diseases | I24 | 414 |
|  | Chronic ischaemic heart disease | I25 |  |
| 7. Dyspepsia | Gastro-oesophageal reflux disease | K21 | 53081 |
|  | Gastro-oesophageal reflux disease with oesophagitis | K210 | 5368 |
|  | Gastro-oesophageal reflux disease without oesophagitis | K219 |  |
|  | Oesophagitis | K20 |  |
|  | Barrett's oesophagus | K227 |  |
|  | Other specified diseases of oesophagus | K228 |  |
|  | Disease of oesophagus, unspecified | K229 |  |
|  | Disorders of oesophagus in diseases classified elsewhere | K23 |  |
|  | Gastric ulcer | K25 |  |
|  | Gastritis and duodenitis | K29 |  |
|  | Duodenal ulcer | K26 |  |
|  | Dyspepsia | K30 |  |
|  | Congenital hiatus hernia | Q401 |  |
|  | Helicobacter pylori [H.pylori] as the cause of diseases classified to other chapters | B980 |  |
| 8. Diabetes | Diabetic polyneuropathy | G632 | 250 |
|  | Diabetic mononeuropathy | G590 |  |
|  | Diabetic retinopathy | H360 |  |
|  | Diabetic cataract | H280 |  |
|  | Insulin-dependent diabetes mellitus | E10 |  |
|  | Non-insulin-dependent diabetes mellitus | E11 |  |
|  | Malnutrition-related diabetes mellitus | E12 |  |
|  | Other specified diabetes mellitus | E13 |  |
|  | Unspecified diabetes mellitus | E14 |  |
|  | Congenital iodine-deficiency syndrome | E00 | 240 |
| 9. Thyroid disorders | Thyrotoxicosis [hyperthyroidism] | E05 | 241 |
|  | Other hypothyroidism | E03 | 242 |
|  | Other non-toxic goitre | E04 | 243 |
|  | Iodine-deficiency-related thyroid disorders and allied conditions | E01 | 244 |
|  | Thyroiditis | E06 | 245 |
|  | Other disorders of thyroid | E07 | 246 |
|  | Subclinical iodine-deficiency hypothyroidism | E02 |  |
| 10. Connective tissue disorders | Myositis | M60 | 710 |
|  | Myopathy, unspecified | G729 |  |
|  | Systemic lupus erythematosus | M32 |  |
|  | Other benign neoplasms of connective and other soft tissue | D21 |  |
|  | Other systemic involvement of connective tissue | M35 |  |
|  | Connective tissue stenosis of neural canal | M994 |  |
|  | Connective tissue and disk stenosis of intervertebral foramina | M997 |  |
|  | Sicca syndrome [Sjogren] | M350 |  |
|  | Dermatopolymyositis | M33 |  |
|  | Localised scleroderma [morphea] | L940 |  |
|  | Linear scleroderma | L941 |  |
|  | Rheumatoid arthritis with involvement of other organs and systems | M053 |  |
|  | Other seropositive rheumatoid arthritis | M058 |  |
|  | Seropositive rheumatoid arthritis, unspecified | M059 |  |
|  | Other rheumatoid arthritis | M06 |  |
|  | Psoriatic and enteropathic arthropathies | M07 |  |
|  | Polymyalgia rheumatica | M353 |  |
|  | Postsurgical malabsorption osteoporosis | M813 |  |
|  | Coeliac disease | K900 |  |
| 11. Chronic Obstructive Pulmonary Disease (COPD) | Simple and mucopurulent chronic bronchitis | J41 | 491 |
|  | Unspecified chronic bronchitis | J42 |  |
|  | Emphysema | J43 | 492 |
|  | Other chronic obstructive pulmonary disease | J44 | 494 |
| 12. Anxiety | Phobic anxiety disorders | F40 | 3000 |
|  | Other anxiety disorders | F41 | 3002 |
|  | Reaction to severe stress, and adjustment disorders | F43 | 3009 |
|  | Posttraumatic stress disorder | F431 |  |
|  | Obsessive-compulsive disorder | F42 | 3003 |
|  | Stress, not elsewhere classified | Z733 | 308 |
|  | Disorders of initiating and maintaining sleep [insomnias] | G470 | 7805 |
|  | Mental disorder, not otherwise specified | F99 |  |
| 13. Irritable bowel syndrome | Irritable bowel syndrome | K58 | 5641 |
| 14. Alcohol problems | Dependence syndrome | **F102** | **3039** |
|  | Alcoholic liver disease | K70 | 291 |
|  | Harmful use | F101 | 303 |
| 15. Other psychoactive substance abuse | Harmful use | F111 | 304 |
|  | Acute intoxication | F100 | 305 |
|  | Dependence syndrome | F112 |  |
|  | Unspecified mental and behavioural disorder | F119 |  |
| 16. Treated constipation | Constipation | K590 | 5640 |
| 17. Stroke/Transient Ischaemic Attack (TIA) | Stroke, not specified as haemorrhage or infarction | I64 | 438 |
|  | Occlusion and stenosis of precerebral arteries, not resulting in cerebral infarction | I65 | 435 |
|  | Subarachnoid haemorrhage | I60 | 430 |
|  | Intracerebral haemorrhage | I61 | 431 |
|  | Other nontraumatic intracranial haemorrhage | I62 | 432 |
|  | Occlusion and stenosis of cerebral arteries, not resulting in cerebral infarction | I66 | 433 |
|  | Cerebral infarction | I63 | 434 |
|  | Acute but ill-defined cerebrovascular disease |  | 436 |
|  | Other and ill-defined cerebrovascular disease |  | 437 |
| 18. Chronic kidney disease | Polycystic kidney, infantile type | Q611 | 75315 |
|  | Polycystic kidney, adult type | Q612 |  |
|  | Polycystic kidney, unspecified | Q613 |  |
|  | Acute renal failure | N17 | 584 |
|  | Chronic renal failure | N18 | 585 |
|  | Unspecified renal failure | N19 | 586 |
|  | Renal complications | E112 | 587 |
|  | Other | N028 | 588 |
| 19. Diverticular disease | Diverticular disease of intestine | K57 | 562 |
| 20. Peripheral vascular disease | Other aneurysm | I72 | 440 |
|  | Other peripheral vascular diseases | I73 | 443 |
|  | Arterial embolism and thrombosis |  | 444 |
| 21. Heart failure | Cardiomyopathy | I42 | 425 |
|  | Heart failure | I50 | 428 |
| 22. Prostate disorders | Hyperplasia of prostate | N40 | 600 |
|  | Inflammatory diseases of prostate | N41 | 601 |
|  | Other disorders of prostate | N42 | 602 |
|  | Disorders of prostate in diseases classified elsewhere | N510 |  |
| 23. Epilepsy | Epilepsy | G40 | 345 |
| 24. Dementia | Creutzfeldt-Jakob disease | A810 | 290 |
|  | Dementia in Alzheimer's disease | F00 |  |
|  | Vascular dementia | F01 |  |
|  | Unspecified dementia | F03 |  |
|  | Delirium superimposed on dementia | F051 |  |
|  | Amnesic syndrome | F106 |  |
|  | Alzheimer's disease | G30 |  |
|  | Other degenerative diseases of nervous system, not elsewhere classified | G31 |  |
|  | Progressive vascular leukoencephalopathy | I673 |  |
| 25. Schizophrenia/bipolar disorder | Schizophrenia | F20 | 295 |
|  | Schizotypal disorder | F21 |  |
|  | Manic episode | F30 | 296 |
|  | Bipolar affective disorder | F31 |  |
| 26. Psoriasis/eczema | Atopic dermatitis | L20 | 696 |
|  | Seborrhoeic dermatitis | L21 | 692 |
|  | Diaper [napkin] dermatitis | L22 |  |
|  | Allergic contact dermatitis | L23 |  |
|  | Irritant contact dermatitis | L24 |  |
|  | Unspecified contact dermatitis | L25 |  |
|  | Exfoliative dermatitis | L26 |  |
|  | Dermatitis due to substances taken internally | L27 |  |
|  | Other dermatitis | L30 |  |
|  | Psoriasis | L40 |  |
|  | Parapsoriasis | L41 |  |
| 27. Inflammatory Bowel Disease | Crohn's disease [regional enteritis] | K50 |  |
|  | Ulcerative colitis | K51 |  |
| 28. Migraine | Migraine | G43 | 346 |
| 29. Chronic sinusitis | Chronic sinusitis | J32 | 473 |
| 30. Anorexia or bulimia | Eating disorders | F50 | 307 |
|  | Anorexia nervosa | F500 |  |
|  | Atypical anorexia nervosa | F501 |  |
|  | Bulimia nervosa | F502 |  |
|  | Atypical bulimia nervosa | F503 |  |
|  | Overeating associated with other psychological disturbances | F504 |  |
|  | Vomiting associated with other psychological disturbances | F505 |  |
|  | Other eating disorders | F508 |  |
|  | Eating disorder, unspecified | F509 |  |
| 31. Bronchiectasis | Bronchiectasis | J47 | 494 |
| 32. Parkinson’s disease | Parkinson's disease | G20 | 332 |
|  | Secondary Parkinsonism | G21 | 3321 |
|  | Parkinsonism in diseases classified elsewhere | G22 | 333 |
|  | Other degenerative diseases of basal ganglia | G23 |  |
|  | Extrapyramidal and movement disorder, unspecified | G259 |  |
|  | Extrapyramidal and movement disorders in diseases classified elsewhere | G26 |  |
|  | Multisystem degeneration | G903 |  |
| 33. Multiple Sclerosis | Multiple sclerosis | G35 | 340 |
| 34. Viral Hepatitis | Acute hepatitis B | B16 | 70 |
|  | Chronic viral hepatitis B with delta-agent | B180 |  |
|  | Chronic viral hepatitis B without delta-agent | B181 |  |
|  | Chronic viral hepatitis C | B182 |  |
|  | Acute hepatitis E | B172 |  |
|  | Other chronic viral hepatitis | B188 |  |
|  | Chronic viral hepatitis, unspecified | B189 |  |
|  | Unspecified viral hepatitis | B19 |  |
| 35. Chronic Liver disease | Oesophageal varices | I85 | 571 |
|  | Toxoplasma hepatitis | B581 |  |
|  | Alcoholic hepatitis | K701 |  |
|  | Toxic liver disease with acute hepatitis | K712 |  |
|  | Toxic liver disease with chronic persistent hepatitis | K713 |  |
|  | Toxic liver disease with chronic lobular hepatitis | K714 |  |
|  | Toxic liver disease with chronic active hepatitis | K715 |  |
|  | Toxic liver disease with hepatitis, not elsewhere classified | K716 |  |
|  | Fibrosis and cirrhosis of liver | K74 |  |
|  | Primary biliary cirrhosis | K743 |  |
| 36. Osteoporosis | Polyarthrosis | M15 | 7330 |
|  | Primary generalised (osteo)arthrosis | M150 |  |
|  | Primary generalized (osteo)arthrosis, Multiple sites | M1500 |  |
|  | Heberden's nodes (with arthropathy) | M151 |  |
|  | Coxarthrosis [arthrosis of hip] | M16 |  |
|  | Gonarthrosis [arthrosis of knee] | M17 |  |
| 37. Chronic fatigue syndrome | Postviral fatigue syndrome | G933 | 7807 |
| 38. Endometriosis | Endometriosis | N80 | 617 |
| 39. Meniere’s disease | Meniere's disease | H810 | 3860 |
| 40. Pernicious Anaemia | Vitamin B12 deficiency anaemia | D51 | 2810 |
| 41. Polycystic ovary | Polycystic ovarian syndrome | E282 | 2564 |
| 42. Hgh cholesterol | Disorders of lipoprotein metabolism and other lipidaemias | E78 | 2720 |
| 43. Heart/cardiac problem | Cardiac arrest | I46 | 393 |
|  | Other cardiac arrhythmias | I49 | 394 |
|  | Complications and ill-defined descriptions of heart disease | I51 | 395 |
|  | Other heart disorders in diseases classified elsewhere | I52 | 396 |
|  | Other cerebrovascular diseases | I67 | 397 |
|  | Cerebrovascular disorders in diseases classified elsewhere | I68 | 398 |
|  | Sequelae of cerebrovascular disease | I69 | 399 |
|  | Atherosclerosis | I70 | 400 |
|  | Acute pericarditis |  | 420 |
|  | Acute and subacute endocarditis |  | 421 |
|  | Acute myocarditis |  | 422 |
|  | Other diseases of pericardium |  | 423 |
|  | Other diseases of endocardium |  | 424 |
|  | Conduction disorders |  | 426 |
|  | Cardiac dysrhythmias |  | 427 |
|  | Ill-defined descriptions and complications of heart disease | | 429 |
| 44. Fracture | Multiple fractures of lumbar spine and pelvis | S327 | 808 |
|  | Multiple fractures of lumbar spine and pelvis (closed) | S3270 |  |
|  | Fracture of femur | S72 | 820 |
|  | Fracture of patella | S820 | 8210 |
|  | Fracture of patella (closed) | S8200 | 824 |
| 45. HIV | Human immunodeficiency virus [HIV] disease resulting in infectious and parasitic diseases | B20 |  |
|  | Human immunodeficiency virus [HIV] disease resulting in malignant neoplasms | B21 |  |
|  | Human immunodeficiency virus [HIV] disease resulting in other specified diseases | B22 |  |
|  | Human immunodeficiency virus [HIV] disease resulting in other conditions | B23 |  |
|  | Unspecified human immunodeficiency virus [HIV] disease | B24 |  |
| 46. Glaucoma | Glaucoma | H40 | 365 |
| 47. Cataract | Senile cataract | H25 | 366 |
|  | Other cataract | H26 |  |
|  | Cataract and other disorders of lens in diseases classified elsewhere | H28 |  |
| 48. AMD | Degeneration of macula and posterior pole | H353 | 3625 |
| 49. Lung Cancer | Malignant neoplasm of bronchus and lung | C34 | 162 |
| 50. non-melanoma skin cancer | Other malignant neoplasms of skin | C44 | 173 |
|  | Mesothelioma | C45 |  |
| 51. Melanoma | Malignant melanoma of skin | C43 | 172 |
| 52. Stomach Cancer | Malignant neoplasm of stomach | C16 | 151 |
| 53. Oesophageal cancer | Malignant neoplasm of oesophagus | C15 | 150 |
| 54. Colon cancer | Malignant neoplasm of colon | C18 | 153 |
| 55. Rectal cancer | Malignant neoplasm of rectum | C20 | 154 |
| 56. prostate cancer | Malignant neoplasm of prostate | C61 | 185 |
| 57. ovarian cancer | Malignant neoplasm of ovary | C56 | 183 |
| 58. Breast cancer | Malignant neoplasm of breast | C50 | 174 |
| 59. Uterine/endometrial cancer | Malignant neoplasm of uterus, part unspecified | C55 | 182 |
| 60. other cancers | Malignant neoplasm of lip | C00 | 140 |
|  | Malignant neoplasm of base of tongue | C01 | 141 |
|  | Malignant neoplasm of other and unspecified parts of tongue | C02 | 142 |
|  | Malignant neoplasm of gum | C03 | 143 |
|  | Malignant neoplasm of floor of mouth | C04 | 144 |
|  | Malignant neoplasm of palate | C05 | 145 |
|  | Malignant neoplasm of other and unspecified parts of mouth | C06 | 146 |
|  | Malignant neoplasm of parotid gland | C07 | 147 |
|  | Malignant neoplasm of other and unspecified major salivary glands | C08 | 148 |
|  | Malignant neoplasm of tonsil | C09 | 149 |
|  | Malignant neoplasm of oropharynx | C10 | 152 |
|  | Malignant neoplasm of nasopharynx | C11 | 155 |
|  | Malignant neoplasm of pyriform sinus | C12 | 156 |
|  | Malignant neoplasm of hypopharynx | C13 | 157 |
|  | Malignant neoplasm of other and ill-defined sites in the lip, oral cavity and pharynx | C14 | 158 |
|  | Malignant neoplasm of small intestine | C17 | 159 |
|  | Malignant neoplasm of rectosigmoid junction | C19 | 160 |
|  | Malignant neoplasm of anus and anal canal | C21 | 161 |
|  | Malignant neoplasm of liver and intrahepatic bile ducts | C22 | 163 |
|  | Malignant neoplasm of gallbladder | C23 | 164 |
|  | Malignant neoplasm of other and unspecified parts of biliary tract | C24 | 165 |
|  | Malignant neoplasm of pancreas | C25 | 166 |
|  | Malignant neoplasm of other and ill-defined digestive organs | C26 | 167 |
|  | Malignant neoplasm of nasal cavity and middle ear | C30 | 171 |
|  | Malignant neoplasm of accessory sinuses | C31 | 175 |
|  | Malignant neoplasm of larynx | C32 | 176 |
|  | Malignant neoplasm of trachea | C33 | 177 |
|  | Malignant neoplasm of thymus | C37 | 180 |
|  | Malignant neoplasm of heart, mediastinum and pleura | C38 | 181 |
|  | Malignant neoplasm of other and ill-defined sites in the respiratory system and intrathoracic organs | C39 | 184 |
|  | Malignant neoplasm of bone and articular cartilage of limbs | C40 | 186 |
|  | Malignant neoplasm of bone and articular cartilage of other and unspecified sites | C41 | 187 |
|  | hematopoietic and reticuloendothelial systems (ICD-O-3 specific) | C42 | 188 |
|  | Kaposi's sarcoma | C46 | 189 |
|  | Malignant neoplasm of peripheral nerves and autonomic nervous system | C47 | 190 |
|  | Malignant neoplasm of retroperitoneum and peritoneum | C48 | 191 |
|  | Malignant neoplasm of other connective and soft tissue | C49 | 192 |
|  | Malignant neoplasm of vulva | C51 | 193 |
|  | Malignant neoplasm of vagina | C52 | 194 |
|  | Malignant neoplasm of cervix uteri | C53 | 195 |
|  | Malignant neoplasm of corpus uteri | C54 | 196 |
|  | Malignant neoplasm of other and unspecified female genital organs | C57 | 197 |
|  | Malignant neoplasm of placenta | C58 | 198 |
|  | Malignant neoplasm of penis | C60 | 200 |
|  | Malignant neoplasm of testis | C62 | 201 |
|  | Malignant neoplasm of other and unspecified male genital organs | C63 | 202 |
|  | Malignant neoplasm of kidney, except renal pelvis | C64 | 203 |
|  | Malignant neoplasm of renal pelvis | C65 | 204 |
|  | Malignant neoplasm of ureter | C66 | 205 |
|  | Malignant neoplasm of bladder | C67 | 206 |
|  | Malignant neoplasm of other and unspecified urinary organs | C68 | 207 |
|  | Malignant neoplasm of eye and adnexa | C69 | 208 |
|  | Malignant neoplasm of meninges | C70 |  |
|  | Malignant neoplasm of brain | C71 |  |
|  | Malignant neoplasm of spinal cord, cranial nerves and other parts of central nervous system | C72 |  |
|  | Malignant neoplasm of thyroid gland | C73 |  |
|  | Malignant neoplasm of adrenal gland | C74 |  |
|  | Malignant neoplasm of other endocrine glands and related structures | C75 |  |
|  | Malignant neoplasm of other and ill-defined sites | C76 |  |
|  | Secondary and unspecified malignant neoplasm of lymph nodes | C77 |  |
|  | Secondary malignant neoplasm of respiratory and digestive organs | C78 |  |
|  | Secondary malignant neoplasm of other sites | C79 |  |
|  | Malignant neoplasm without specification of site | C80 |  |
|  | Hodgkin's disease | C81 |  |
|  | Follicular [nodular] non-Hodgkin's lymphoma | C82 |  |
|  | Diffuse non-Hodgkin's lymphoma | C83 |  |
|  | Peripheral and cutaneous T-cell lymphomas | C84 |  |
|  | Other and unspecified types of non-Hodgkin's lymphoma | C85 |  |
|  | Other specified types of T/NK-cell lymphoma | C86 |  |
|  | Malignant immunoproliferative diseases | C88 |  |
|  | Multiple myeloma and malignant plasma cell neoplasms | C90 |  |
|  | Lymphoid leukaemia | C91 |  |
|  | Myeloid leukaemia | C92 |  |
|  | Monocytic leukaemia | C93 |  |
|  | Other leukaemias of specified cell type | C94 |  |
|  | Leukaemia of unspecified cell type | C95 |  |
|  | Other and unspecified malignant neoplasms of lymphoid, haematopoietic and related tissue | C96 |  |
|  | Malignant neoplasms of independent (primary) multiple sites | C97 |  |

Table S3. Matching analysis for diseases that were inversely associated with incident mortality

|  | Participants | Events | Person-years | Incidence rate | HR (95% CI), Model 1* | HR (95% CI), Model 2 | HR (95% CI), Model 3 |
| --- | --- | --- | --- | --- | --- | --- | --- |
| Prostate disorders^†^ |  |  |  |  |  |  |  |
| No | 8248 | 1122 | 93647 | 11.98 | Reference | Reference | Reference |
| Yes | 8248 | 938 | 94292 | 9.95 | 0.83(0.76-0.91) | 0.86(0.79-0.94) | 1.09(0.92-1.29) |
| Endometriosis^‡^ |  |  |  |  |  |  |  |
| No | 4053 | 157 | 47687 | 3.29 | Reference | Reference | Reference |
| Yes | 4053 | 131 | 47747 | 2.74 | 0.85(0.67-1.07) | 0.83(0.66-1.05) | 1.44(0.38-5.51) |
| Migraine |  |  |  |  |  |  |  |
| No | 14378 | 733 | 168482 | 4.35 | Reference | Reference | Reference |
| Yes | 14378 | 641 | 170146 | 3.77 | 0.86(0.77-0.95) | 0.89(0.80-0.99) | 1.89(0.93-3.84) |

*Cox proportional hazard regression models were used to examine the association between individual diseases at baseline and incident mortality. Model 1 was adjusted for age (gender was additionally adjusted for in the analysis for migraine); Model 2 was adjusted for Model 1 plus ethnicity, education, income, BMI, smoking, physical acidity, alcohol consumption, sleep duration, diet, blood pressure, HDL-C, triglycerides, and HbA1c; Model 3 was adjusted for Model 2 plus all other diseases examined. Individuals who died in the first year of follow-up were excluded from the analysis.

^†^Analysis was conducted among men only.

^‡^Analysis was conducted among women only.

**Table S4. Classification of diseases**

| **Group** | **Disease** |
| --- | --- |
| Hypertension | Hypertension |
| Hypercholesterolemia | High cholesterol |
| CVD | Coronary Heart Disease |
|  | Heart failure |
|  | Stroke |
|  | Atrial Fibrillation |
|  | Peripheral vascular disease |
|  | Heart/cardiac problem |
| Cancer | Lung Cancer |
|  | Melanoma |
|  | Stomach Cancer |
|  | Oesophageal cancer |
|  | Colon cancer |
|  | Rectal cancer |
|  | Prostate cancer |
|  | Ovarian cancer |
|  | Breast cancer |
|  | Uterine cancer |
|  | Other cancer |
| Diabetes | Diabetes |
| Painful conditions | Painful conditions |
| Musculoskeletal disorders | Osteoporosis |
|  | Fracture |
|  | Connective tissue disorders |
| Nonpsychotic mental disorders | Depression |
|  | Anxiety |
| Neurodegenerative disorders | Dementia |
|  | Parkinson |
| Neurologic conditions | Multiple Sclerosis |
|  | Migraine |
|  | Epilepsy |
| Psychotic disorders | Schizophrenia |
|  | Alcohol problems |
|  | Other psychoactive substance abuse |
| Respiratory disease | Asthma |
|  | COPD |
|  | Bronchiectasis |
| Thyroid disorders | Thyroid disorders |
| Digestive disorders | Anorexia |
|  | Dyspepsia |
|  | Constipation |
|  | Inflammatory Bowel Disease |
| CKD | CKD |
| Liver diseases | Viral Hepatitis |
|  | Chronic Liver disease |

**Table S5. Death and the number of diseases**

| **Number of** | **Death** | |  |
| --- | --- | --- | --- |
| **diseases*** | No | Yes | Incidence (%) |
| 0 | 104986 | 702 | 0.7 |
| 1 | 108218 | 2406 | 2.2 |
| 2 | 83022 | 4010 | 4.6 |
| 3 | 58972 | 4934 | 7.7 |
| 4 | 40048 | 4963 | 11 |
| 5 | 25724 | 4545 | 15 |
| ≥6 | 32082 | 9813 | 23.4 |

*Number of diseases was computed based on the 16 groups of diseases in Table S4.

**Table S6. Baseline characteristics of participants by mortality**

|  | Mortality | | P-value* |
| --- | --- | --- | --- |
|  | No | Yes |  |
| Age (years) | 57 (50-63) | 63 (58-67) | <0.0001 |
| Gender |  |  | <0.0001 |
| Female | 249160 (55.0) | 12575 (40.1) |  |
| Male | 203892 (45.0) | 18798 (59.9) |  |
| Ethnicity |  |  | <0.0001 |
| Whites | 425758 (94.0) | 30082 (95.9) |  |
| Non-whites | 24842 (5.5) | 1072 (3.4) |  |
| Unknown | 2452 (0.5) | 219 (0.7) |  |
| Education |  |  | <0.0001 |
| 0-5 years | 70667 (15.6) | 9149 (29.2) |  |
| 6-12 years | 223551 (49.3) | 13983 (44.6) |  |
| ≥13 years | 150118 (33.1) | 7346 (23.4) |  |
| Missing | 8716 (1.9) | 895 (2.9) |  |
| Household income (pounds) |  |  | <0.0001 |
| <18,000 | 81929 (18.1) | 10190 (32.5) |  |
| 18,000-30,999 | 97386 (21.5) | 7281 (23.2) |  |
| 31,000-51,999 | 103270 (22.8) | 4782 (15.2) |  |
| 52,000-100,000 | 82026 (18.1) | 2633 (8.4) |  |
| >100,000 | 21968 (4.8) | 615 (2.0) |  |
| Unknown | 17647 (3.9) | 1843 (5.9) |  |
| Not answered | 48826 (10.8) | 4029 (12.8) |  |
| Physical activity (MET-minutes/week) | 2439 (1017-3003) | 2466 (862-2730) | <0.0001 |
| Diet quality^†^ |  |  | <0.0001 |
| Low | 168577 (37.2) | 14402 (45.9) |  |
| High | 284475 (62.8) | 16971 (54.1) |  |
| Alcohol consumption |  |  | <0.0001 |
| Never | 19633 (4.3) | 1542 (4.9) |  |
| Previous | 15051 (3.3) | 2034 (6.5) |  |
| Current | 416945 (92.0) | 27663 (88.2) |  |
| Missing | 1423 (0.3) | 134 (0.4) |  |
| Smoking |  |  |  |
| Never | 252126 (55.7) | 11975 (38.2) |  |
| Former | 153477 (33.9) | 13056 (41.6) |  |
| Current | 44932 (9.9) | 6071 (19.4) |  |
| Missing | 2517 (0.6) | 271 (0.9) |  |
| Sleep duration (hours) |  |  | 0.22 |
| <7 | 109745 (24.2) | 8193 (26.1) |  |
| 7-9 | 332568 (73.4) | 21536 (68.6) |  |
| >9 | 7412 (1.6) | 1281 (4.1) |  |
| Missing | 3327 (0.7) | 363 (1.2) |  |
| BMI (kg/m^2^) | 27.31 ± 4.65 | 28.12 ± 5.16 | <0.0001 |
| Total cholesterol (mmol/L) | 5.71 ± 1.09 | 5.48 ± 1.21 | <0.0001 |
| HDL-C (mmol/L) | 1.45 ± 0.35 | 1.39 ± 0.37 | <0.0001 |
| LDL-C (mmol/L) | 3.57 ± 0.83 | 3.41 ± 0.90 | <0.0001 |
| Triglycerides | 1.54 (1.06-2.07) | 1.71 (1.17-2.23) | <0.0001 |
| HbA1c (mmol/mol) | 35.98 ± 6.17 | 38.53 ± 9.19 | <0.0001 |
| DBP (mmHg) | 82.10 ± 9.80 | 82.28 ± 10.31 | 0.0013 |
| SBP (mmHg) | 137.18 ± 17.96 | 141.77 ± 19.20 | <0.0001 |
| Genetic risk score^‡^ | 0.50 (0.46-0.53) | 0.49 (0.45-0.53) | <0.0001 |

Data are means ± standard deviations, medians (interquartile range), or N (%). BMI, body mass index; CVD, cardiovascular disease; DBP, diastolic blood pressure; HbA1c, glycated haemoglobin; HDL-C, high-density lipoprotein cholesterol; LDL-C, low-density lipoprotein cholesterol; MET, metabolic equivalent; SBP, systolic blood pressure.

*Baseline data were expressed as means ± standard deviations, medians (interquartile ranges), or frequency (percentage) according to the number of types of diseases. ANOVA analysis for normally distributed continuous variables, Wilcoxon Rank Sum Test for skewed continuous variables, and Chi-square test for categorical variables were used to examine the difference in baseline characteristics by mortality.

^†^Diet score was computed based on seven commonly eaten food groups following recommendations on dietary priorities for cardiometabolic health with higher score representing healthier diet. High diet quality was defined as diet score≥4.

^‡^The genetic risk score (GRS) for longevity was compuated using 78 single-nucleotide polymorphisms.

**Table S7. Disease contribution among individuals who were diagnosed with one disease only in life-course**

| Disease | All mortality  (n=2433) | Cancer mortality  (n=1286) | CVD mortality  (n=573) |
| --- | --- | --- | --- |
| Hypertension | 253 (10.4) | 31 (2.4) | 136 (23.7) |
| Hypercholesterolemia | 45 (1.9) | 10 (0.8) | 19 (3.3) |
| CVD | 184 (7.6) | 15 (1.2) | 123 (21.5) |
| Cancer | 1239 (50.9) | 1156 (89.9) | 36 (6.3) |
| Diabetes | 19 (0.8) | 4 (0.3) | 12 (2.1) |
| Painful conditions | 93 (3.8) | 16 (1.2) | 39 (6.8) |
| Musculoskeletal disorders | 85 (3.5) | 9 (0.7) | 37 (6.5) |
| Nonpsychotic mental disorders | 69 (2.8) | 3 (0.2) | 24 (4.2) |
| Neurodegenerative disorders | 94 (3.9) | 3 (0.2) | 5 (0.9) |
| Neurologic conditions | 36 (1.5) | 3 (0.2) | 8 (1.4) |
| Psychotic disorders | 36 (1.5) | 1 (0.1) | 16 (2.8) |
| Respiratory disease | 111 (4.6) | 9 (0.7) | 46 (8) |
| Thyroid disorders | 27 (1.1) | 3 (0.2) | 12 (2.1) |
| Digestive disorders | 124 (5.1) | 21 (1.6) | 55 (9.6) |
| CKD | 12 (0.5) | 1 (0.1) | 2 (0.4) |
| Liver diseases | 6 (0.3) | 1 (0.1) | 3 (0.5) |

CKD, chronic kidney disease; CVD, cardiovascular disease.

**Table S8. Risk for mortality associated with individual diseases of interest at baseline among individuals with complete data**

|  | Events/Person-years | |  | HR (95% CI)* | | |
| --- | --- | --- | --- | --- | --- | --- |
|  | No disease | Disease |  | Model 1 | Model 2 | Model 3 |
| Hypertension^†^ | 9423/2356348 | 6146/767884 |  | 1.40(1.36-1.45) | 1.27(1.22-1.31) | 1.18(1.13-1.22) |
| High cholesterol^†^ | 12519/2762708 | 3050/361525 |  | 1.21(1.16-1.26) | 1.06(1.02-1.11) | 0.96(0.92-1.00) |
| Coronary heart disease^†^ | 13777/3002552 | 1792/121681 |  | 1.85(1.76-1.95) | 1.50(1.42-1.58) | 1.39(1.32-1.47) |
| Atrial fibrillation^†^ | 15328/3102882 | 241/21351 |  | 1.36(1.19-1.54) | 1.40(1.23-1.59) | 1.34(1.18-1.53) |
| Heart failure^†^ | 15518/3122850 | 51/1382 |  | 5.39(4.10-7.10) | 4.10(3.11-5.40) | 3.05(2.31-4.03) |
| Stroke^†^ | 15011/3091086 | 558/33146 |  | 2.30(2.11-2.50) | 1.70(1.56-1.86) | 1.53(1.40-1.67) |
| Peripheral vascular disease^†^ | 15454/3117178 | 115/7055 |  | 2.76(2.30-3.31) | 2.21(1.84-2.66) | 1.99(1.65-2.40) |
| Other cardiac problem^†^ | 15487/3115610 | 82/8623 |  | 1.55(1.25-1.93) | 1.46(1.17-1.81) | 1.24(0.99-1.54) |
| Diabetes^†^ | 13964/3007159 | 1605/117073 |  | 2.11(2.01-2.23) | 1.56(1.48-1.66) | 1.46(1.38-1.55) |
| COPD^†^ | 15346/3116272 | 223/7960 |  | 4.06(3.56-4.63) | 2.64(2.31-3.02) | 2.51(2.20-2.87) |
| Asthma^†^ | 13697/2764571 | 1872/359662 |  | 1.15(1.10-1.21) | 1.12(1.07-1.18) | 1.07(1.02-1.12) |
| Bronchiectasis^†^ | 15501/3117939 | 68/6293 |  | 1.63(1.29-2.07) | 1.75(1.38-2.22) | 1.58(1.24-2.01) |
| Depression^†^ | 14553/2954128 | 1016/170105 |  | 1.49(1.40-1.59) | 1.22(1.15-1.31) | 1.17(1.10-1.25) |
| Anxiety^†^ | 15353/3083937 | 216/40295 |  | 1.23(1.07-1.40) | 1.10(0.96-1.26) | 1.03(0.90-1.18) |
| Schizophrenia^†^ | 15443/3113125 | 126/11108 |  | 2.61(2.19-3.11) | 1.85(1.55-2.21) | 1.90(1.59-2.27) |
| Dementia^†^ | 15535/3123719 | 34/513 |  | 6.17(4.36-8.73) | 6.42(4.55-9.05) | 7.30(5.18-10.29) |
| Parkinson’s disease^†^ | 15416/3119880 | 153/4352 |  | 4.59(3.92-5.39) | 4.76(4.06-5.59) | 4.66(3.97-5.47) |
| Multiple sclerosis^†^ | 15519/3114824 | 50/9409 |  | 1.43(1.09-1.89) | 1.28(0.97-1.70) | 1.36(1.03-1.80) |
| Alcohol problems^†^ | 15474/3119922 | 95/4310 |  | 4.57(3.73-5.59) | 2.34(1.90-2.88) | 1.98(1.60-2.44) |
| Psychoactive substance abuse^†^ | 15559/3123809 | 10/424 |  | 5.90(3.17-10.96) | 3.20(1.72-5.95) | 2.15(1.14-4.04) |
| Migraine | 15240/3033709 | 329/90524 |  | 0.94(0.84-1.04) | 0.96(0.86-1.07) | 1.00(0.89-1.11) |
| Epilepsy^†^ | 15384/3102378 | 185/21854 |  | 1.76(1.52-2.04) | 1.58(1.37-1.83) | 1.55(1.34-1.79) |
| Painful conditions^†^ | 13742/2823862 | 1827/300370 |  | 1.12(1.07-1.18) | 1.02(0.97-1.07) | 1.00(0.95-1.05) |
| Chronic fatigue syndrome | 15505/3111070 | 64/13163 |  | 1.25(0.98-1.60) | 1.18(0.92-1.50) | 1.16(0.91-1.49) |
| Connective tissue disorders^†^ | 15057/3063036 | 512/61196 |  | 1.59(1.45-1.74) | 1.42(1.30-1.56) | 1.41(1.29-1.54) |
| Osteoporosis^†^ | 13960/2897190 | 1609/227043 |  | 1.10(1.05-1.16) | 0.98(0.93-1.04) | 0.98(0.93-1.03) |
| Fracture^†^ | 15529/3119659 | 40/4574 |  | 1.73(1.27-2.36) | 1.75(1.28-2.38) | 1.69(1.24-2.31) |
| Anorexia | 15564/3121871 | 5/2362 |  | 0.93(0.39-2.24) | 0.83(0.34-1.98) | 0.72(0.30-1.74) |
| Dyspepsia^†^ | 14063/2900166 | 1506/224066 |  | 1.13(1.07-1.19) | 1.01(0.96-1.07) | 0.98(0.93-1.04) |
| Treated constipation | 15560/3122329 | 9/1904 |  | 1.01(0.53-1.95) | 0.98(0.51-1.88) | 0.87(0.45-1.67) |
| Pernicious anaemia^†^ | 15492/3115702 | 77/8531 |  | 1.75(1.40-2.19) | 1.54(1.23-1.92) | 1.42(1.13-1.78) |
| Diverticular disease | 15375/3093249 | 194/30984 |  | 0.96(0.83-1.11) | 0.89(0.77-1.02) | 0.84(0.73-0.97) |
| Inflammatory bowel disease^†^ | 15381/3098400 | 188/25833 |  | 1.46(1.26-1.68) | 1.39(1.21-1.61) | 1.33(1.15-1.54) |
| Irritable bowel syndrome^†^ | 15315/3056461 | 254/67772 |  | 0.86(0.76-0.98) | 0.84(0.74-0.95) | 0.84(0.74-0.95) |
| Chronic kidney disease^†^ | 15431/3117283 | 138/6950 |  | 3.85(3.25-4.55) | 3.26(2.76-3.86) | 2.54(2.14-3.00) |
| Viral hepatitis^†^ | 15506/3115591 | 63/8642 |  | 1.42(1.11-1.82) | 1.47(1.15-1.89) | 1.38(1.08-1.77) |
| Chronic liver disease^†^ | 15484/3118553 | 85/5680 |  | 2.85(2.30-3.52) | 2.27(1.84-2.81) | 2.07(1.67-2.57) |
| Prostate disorders^†^ | 9566/1490457 | 482/53939 |  | 0.84(0.77-0.92) | 0.88(0.80-0.96) | 0.89(0.82-0.98) |
| Endometriosis | 5459/1555473 | 62/24364 |  | 0.89(0.69-1.15) | 0.88(0.69-1.14) | 0.90(0.70-1.16) |
| Polycystic ovary | 5518/1575556 | 3/4281 |  | 0.43(0.14-1.34) | 0.36(0.12-1.13) | 0.37(0.12-1.14) |
| HIV^†^ | 15547/3121435 | 22/2798 |  | 2.03(1.33-3.08) | 1.79(1.18-2.72) | 1.59(1.04-2.42) |
| Thyroid disorders^†^ | 14715/2960668 | 854/163565 |  | 1.12(1.04-1.20) | 1.04(0.97-1.12) | 1.01(0.94-1.08) |
| Psoriasis/eczema^†^ | 14936/3007104 | 633/117128 |  | 1.14(1.05-1.23) | 1.09(1.01-1.18) | 1.08(0.99-1.17) |
| Chronic sinusitis | 15479/3104720 | 90/19513 |  | 0.91(0.74-1.11) | 0.93(0.75-1.14) | 0.96(0.78-1.18) |
| Meniere’s disease | 15518/3115965 | 51/8267 |  | 1.01(0.77-1.33) | 0.97(0.74-1.28) | 0.95(0.72-1.25) |
| Glaucoma | 15338/3093900 | 231/30333 |  | 1.02(0.89-1.16) | 1.02(0.89-1.16) | 0.97(0.85-1.11) |
| Cataract^†^ | 15182/3083741 | 387/40491 |  | 1.38(1.25-1.53) | 1.34(1.21-1.49) | 1.24(1.12-1.38) |
| AMD | 15554/3122666 | 15/1567 |  | 1.51(0.91-2.50) | 1.53(0.92-2.54) | 1.48(0.89-2.46) |
| Lung cancer^†^ | 15500/3123083 | 69/1149 |  | 8.34(6.58-10.57) | 7.31(5.76-9.26) | 4.85(3.81-6.16) |
| Skin cancer | 15533/3119691 | 36/4542 |  | 1.13(0.82-1.57) | 1.14(0.82-1.58) | 1.14(0.82-1.58) |
| Melanoma^†^ | 15363/3100929 | 206/23304 |  | 1.48(1.29-1.69) | 1.52(1.32-1.74) | 1.49(1.29-1.71) |
| Stomach cancer^†^ | 15548/3123422 | 21/811 |  | 3.56(2.32-5.45) | 3.17(2.07-4.87) | 2.52(1.63-3.87) |
| Oesophageal cancer^†^ | 15531/3123259 | 38/974 |  | 4.80(3.49-6.59) | 3.84(2.79-5.29) | 3.89(2.83-5.36) |
| Colon cancer^†^ | 15409/3115468 | 160/8765 |  | 2.25(1.93-2.63) | 2.23(1.91-2.61) | 2.02(1.72-2.36) |
| Rectal cancer^†^ | 15536/3122519 | 33/1713 |  | 2.76(1.96-3.89) | 2.75(1.96-3.87) | 2.25(1.59-3.17) |
| Prostate cancer^†^ | 9687/1523387 | 361/21009 |  | 1.61(1.45-1.79) | 1.70(1.53-1.89) | 1.71(1.54-1.90) |
| Ovarian cancer^†^ | 5462/1575667 | 59/4169 |  | 3.39(2.63-4.39) | 3.14(2.43-4.06) | 3.14(2.42-4.07) |
| Breast cancer^†^ | 4913/1521867 | 608/57969 |  | 2.49(2.29-2.71) | 2.50(2.30-2.72) | 2.49(2.28-2.71) |
| Uterine cancer^†^ | 15523/3117961 | 46/6272 |  | 1.44(1.07-1.92) | 1.27(0.95-1.69) | 1.05(0.79-1.41) |
| Other cancers^†^ | 14397/3017726 | 1172/106507 |  | 2.00(1.88-2.12) | 1.99(1.87-2.11) | 1.89(1.78-2.01) |

*Cox proportional hazard regression models were used to examine the association between each of the 60 major diseases at baseline and incident mortality among those with complete data (covariates). Model 1 was adjusted for age and gender; Model 2 was adjusted for Model 1 plus ethnicity, education, income, BMI, smoking, physical acidity, alcohol consumption, sleep duration, diet, blood pressure, HDL-C, triglycerides, and HbA1c. Model 3 was adjusted for Model 2 plus all other 59 chronic diseases. The analysis for breast cancer, ovarian cancer, endometriosis, and polycystic ovary was conducted among women only while the analysis for prostate cancer and prostate disorders was conducted among men only. Individuals with disease diagnosed in the last year before mortality were excluded from the analysis.

^†^Refers to significant associations after adjustment for false discovery rate at a 5% level using Benjamin-Hochberg's procedure.

**Table S9. Prevalence of individual diseases by number of diseases in life-course**

|  | Number of diseases in life-course among individuals died during follow-up | | | | | | | P-value |
| --- | --- | --- | --- | --- | --- | --- | --- | --- |
|  | 0 (n=702) | 1 (n=2406) | 2 (n=4010) | 3 (n=4934) | 4 (n=4963) | 5 (n=4545) | ≥6 (n=9813) |  |
| Hypertension | 0 | 215(8.9) | 731(18.2) | 1391(28.2) | 1849(37.3) | 2159(47.5) | 5644(57.5) | <0.0001 |
| High cholesterol | 0 | 41(1.7) | 225(5.6) | 513(10.4) | 847(17.1) | 1105(24.3) | 3243(33.0) | <0.0001 |
| Coronary heart disease | 0 | 28(1.2) | 111(2.8) | 266(5.4) | 468(9.4) | 652(14.3) | 2334(23.8) | <0.0001 |
| Atrial fibrillation | 0 | 10(0.4) | 38(0.9) | 64(1.3) | 82(1.7) | 87(1.9) | 245(2.5) | <0.0001 |
| Heart failure | 0 | 1(0.0) | 4(0.1) | 9(0.2) | 12(0.2) | 20(0.4) | 51(0.5) | <0.0001 |
| Stroke | 0 | 7(0.3) | 36(0.9) | 89(1.8) | 150(3.0) | 216(4.8) | 751(7.7) | <0.0001 |
| Peripheral vascular disease | 0 | 2(0.1) | 10(0.2) | 13(0.3) | 27(0.5) | 35(0.8) | 132(1.3) | <0.0001 |
| Other cardiac problem | 0 | 2(0.1) | 5(0.1) | 16(0.3) | 43(0.9) | 33(0.7) | 88(0.9) | <0.0001 |
| Diabetes | 0 | 10(0.4) | 72(1.8) | 176(3.6) | 359(7.2) | 520(11.4) | 2092(21.3) | <0.0001 |
| COPD | 0 | 10(0.4) | 27(0.7) | 28(0.6) | 55(1.1) | 76(1.7) | 256(2.6) | <0.0001 |
| Asthma | 0 | 70(2.9) | 260(6.5) | 400(8.1) | 514(10.4) | 600(13.2) | 1890(19.3) | <0.0001 |
| Bronchiectasis | 0 | 2(0.1) | 9(0.2) | 19(0.4) | 22(0.4) | 18(0.4) | 65(0.7) | <0.0001 |
| Depression | 0 | 37(1.5) | 129(3.2) | 212(4.3) | 262(5.3) | 315(6.9) | 1079(11.0) | <0.0001 |
| Anxiety | 0 | 8(0.3) | 39(1.0) | 52(1.1) | 63(1.3) | 70(1.5) | 210(2.1) | <0.0001 |
| Schizophrenia | 0 | 11(0.5) | 21(0.5) | 31(0.6) | 36(0.7) | 23(0.5) | 137(1.4) | <0.0001 |
| Dementia | 0 | 7(0.3) | 9(0.2) | 12(0.2) | 9(0.2) | 8(0.2) | 19(0.2) | 0.60 |
| Parkinson’s disease | 0 | 12(0.5) | 37(0.9) | 51(1.0) | 53(1.1) | 60(1.3) | 125(1.3) | <0.0001 |
| Multiple sclerosis | 0 | 7(0.3) | 20(0.5) | 32(0.6) | 40(0.8) | 41(0.9) | 63(0.6) | 0.0084 |
| Alcohol problems | 0 | 2(0.1) | 6(0.1) | 21(0.4) | 27(0.5) | 29(0.6) | 87(0.9) | <0.0001 |
| Psychoactive substance abuse | 0 | 0 | 0 | 1(0.0) | 5(0.1) | 3(0.1) | 12(0.1) | 0.0039 |
| Migraine | 0 | 14(0.6) | 43(1.1) | 86(1.7) | 106(2.1) | 91(2.0) | 252(2.6) | <0.0001 |
| Epilepsy | 0 | 11(0.5) | 36(0.9) | 55(1.1) | 64(1.3) | 70(1.5) | 205(2.1) | <0.0001 |
| Painful conditions | 0 | 61(2.5) | 196(4.9) | 374(7.6) | 487(9.8) | 598(13.2) | 1890(19.3) | <0.0001 |
| Chronic fatigue syndrome | 3(0.4) | 11(0.5) | 11(0.3) | 17(0.3) | 17(0.3) | 13(0.3) | 46(0.5) | 0.56 |
| Connective tissue disorders | 0 | 18(0.7) | 61(1.5) | 109(2.2) | 147(3.0) | 216(4.8) | 574(5.8) | <0.0001 |
| Osteoporosis | 0 | 37(1.5) | 192(4.8) | 339(6.9) | 465(9.4) | 545(12.0) | 1818(18.5) | <0.0001 |
| Fracture | 0 | 2(0.1) | 8(0.2) | 8(0.2) | 9(0.2) | 7(0.2) | 31(0.3) | 0.0111 |
| Anorexia | 0 | 1(0.0) | 1(0.0) | 6(0.1) | 2(0.0) | 2(0.0) | 6(0.1) | 0.76 |
| Dyspepsia | 0 | 46(1.9) | 197(4.9) | 342(6.9) | 453(9.1) | 531(11.7) | 1538(15.7) | <0.0001 |
| Treated constipation | 0 | 1(0.0) | 0 | 6(0.1) | 2(0.0) | 3(0.1) | 15(0.2) | 0.0152 |
| Pernicious anaemia | 0 | 1(0.0) | 5(0.1) | 14(0.3) | 21(0.4) | 33(0.7) | 110(1.1) | <0.0001 |
| Diverticular disease | 6(0.9) | 15(0.6) | 34(0.8) | 38(0.8) | 66(1.3) | 57(1.3) | 216(2.2) | <0.0001 |
| Chronic kidney disease | 0 | 0 | 7(0.2) | 7(0.1) | 23(0.5) | 36(0.8) | 223(2.3) | <0.0001 |
| Inflammatory bowel disease | 11(1.6) | 26(1.1) | 48(1.2) | 52(1.1) | 62(1.2) | 61(1.3) | 133(1.4) | 0.67 |
| Irritable bowel syndrome | 3(0.4) | 21(0.9) | 50(1.2) | 96(1.9) | 88(1.8) | 72(1.6) | 239(2.4) | <0.0001 |
| Viral hepatitis | 0 | 1(0.0) | 5(0.1) | 15(0.3) | 16(0.3) | 14(0.3) | 51(0.5) | <0.0001 |
| Chronic liver disease | 0 | 0 | 4(0.1) | 10(0.2) | 22(0.4) | 22(0.5) | 116(1.2) | <0.0001 |
| Prostate disorders | 16(2.3) | 44(1.8) | 89(2.2) | 113(2.3) | 138(2.8) | 126(2.8) | 362(3.7) | <0.0001 |
| Endometriosis | 0 | 11(0.5) | 18(0.4) | 18(0.4) | 24(0.5) | 23(0.5) | 37(0.4) | 0.80 |
| Polycystic ovary | 0 | 1(0.0) | 1(0.0) | 0 | 4(0.1) | 2(0.0) | 4(0.0) | 0.46 |
| HIV | 4(0.6) | 7(0.3) | 9(0.2) | 2(0.0) | 7(0.1) | 10(0.2) | 6(0.1) | <0.0001 |
| Thyroid disorders | 0 | 26(1.1) | 104(2.6) | 215(4.4) | 246(5.0) | 322(7.1) | 939(9.6) | <0.0001 |
| Psoriasis/eczema | 22(3.1) | 73(3.0) | 147(3.7) | 166(3.4) | 176(3.5) | 195(4.3) | 402(4.1) | <0.0001 |
| Chronic sinusitis | 6(0.9) | 5(0.2) | 16(0.4) | 27(0.5) | 21(0.4) | 25(0.6) | 65(0.7) | 0.64 |
| Meniere’s disease | 0 | 7(0.3) | 9(0.2) | 17(0.3) | 12(0.2) | 12(0.3) | 45(0.5) | 0.0186 |
| Glaucoma | 4(0.6) | 27(1.1) | 67(1.7) | 76(1.5) | 67(1.3) | 70(1.5) | 205(2.1) | <0.0001 |
| Cataract | 8(1.1) | 32(1.3) | 73(1.8) | 82(1.7) | 110(2.2) | 133(2.9) | 354(3.6) | <0.0001 |
| AMD | 0 | 1(0.0) | 3(0.1) | 3(0.1) | 3(0.1) | 4(0.1) | 13(0.1) | 0.0683 |
| Lung cancer | 0 | 15(0.6) | 27(0.7) | 21(0.4) | 19(0.4) | 29(0.6) | 51(0.5) | 0.83 |
| Skin cancer | 0 | 5(0.2) | 13(0.3) | 15(0.3) | 8(0.2) | 5(0.1) | 23(0.2) | 0.60 |
| Melanoma | 0 | 19(0.8) | 46(1.1) | 47(1.0) | 63(1.3) | 42(0.9) | 116(1.2) | 0.0415 |
| Stomach cancer | 0 | 1(0.0) | 6(0.1) | 5(0.1) | 15(0.3) | 5(0.1) | 12(0.1) | 0.53 |
| Oesophageal cancer | 0 | 6(0.2) | 10(0.2) | 17(0.3) | 17(0.3) | 12(0.3) | 22(0.2) | 0.89 |
| Colon cancer | 0 | 31(1.3) | 43(1.1) | 59(1.2) | 44(0.9) | 38(0.8) | 89(0.9) | 0.27 |
| Rectal cancer | 0 | 3(0.1) | 10(0.2) | 8(0.2) | 12(0.2) | 13(0.3) | 15(0.2) | 0.79 |
| Prostate cancer | 0 | 37(1.5) | 70(1.7) | 102(2.1) | 120(2.4) | 109(2.4) | 232(2.4) | <0.0001 |
| **Ovarian cancer** | **0** | **22(0.9)** | **22(0.5)** | **38(0.8)** | **22(0.4)** | **24(0.5)** | **33(0.3)** | **0.0043** |
| **Breast cancer** | **0** | **172(7.1)** | **280(7.0)** | **272(5.5)** | **214(4.3)** | **191(4.2)** | **340(3.5)** | **<0.0001** |
| Uterine cancer | 0 | 8(0.3) | 10(0.2) | 19(0.4) | 20(0.4) | 16(0.4) | 55(0.6) | 0.0038 |
| Other cancers | 0 | 160(6.7) | 282(7.0) | 371(7.5) | 399(8.0) | 379(8.3) | 862(8.8) | <0.0001 |

***Chi-square test was used to examine the difference in prevalence between individuals with difference number of diseases.**

**^†^These diseases were not included in the analysis of the number of diseases because the prevalence was smaller than 0.5%.**

**Table S10. Number of events at baseline captured by self-reported and inpatient data**

| Disease | All | Self-reported data | |  | Inpatient data | |  | Kappa coefficient  (95% CI)* |
| --- | --- | --- | --- | --- | --- | --- | --- | --- |
|  |  | Events (%) | % accounting for all events | | Events (%) | % accounting  for all events | | |
| Hypertension | 136520 (27.17) | 130975 (26.06) | 95.94 |  | 92414 (18.39) | 67.69 |  | 0.72 (0.71-0.72) |
| High cholesterol | 69789 (13.89) | 61631 (12.26) | 88.31 |  | 34648 (6.9) | 49.65 |  | 0.51 (0.5-0.51) |
| Coronary heart disease | 26897 (5.35) | 22727 (4.52) | 84.5 |  | 23597 (4.7) | 87.73 |  | 0.83 (0.83-0.83) |
| Atrial fibrillation | 7638 (1.52) | 3651 (0.73) | 47.8 |  | 7187 (1.43) | 94.10 |  | 0.59 (0.58-0.6) |
| Heart failure | 16693 (3.32) | 315 (0.06) | 1.89 |  | 16612 (3.31) | 99.51 |  | 0.03 (0.02-0.03) |
| Other cardiac problem | 6178 (1.23) | 1624 (0.32) | 26.29 |  | 4973 (0.99) | 80.50 |  | 0.12 (0.11-0.13) |
| Stroke | 7941 (1.58) | 6682 (1.33) | 84.15 |  | 3783 (0.75) | 47.64 |  | 0.48 (0.47-0.49) |
| Peripheral vascular disease | 3120 (0.62) | 1279 (0.25) | 40.99 |  | 2413 (0.48) | 77.34 |  | 0.31 (0.29-0.33) |
| Diabetes | 23783 (4.73) | 21723 (4.32) | 91.34 |  | 19528 (3.89) | 82.11 |  | 0.84 (0.84-0.84) |
| COPD | 3601 (0.72) | 1665 (0.33) | 46.24 |  | 3231 (0.64) | 89.73 |  | 0.53 (0.51-0.54) |
| Asthma | 60162 (11.97) | 58274 (11.6) | 96.86 |  | 34587 (6.88) | 57.49 |  | 0.68 (0.67-0.68) |
| Bronchiectasis | 1475 (0.29) | 1135 (0.23) | 76.95 |  | 1096 (0.22) | 74.31 |  | 0.68 (0.65-0.7) |
| Depression | 30549 (6.08) | 28206 (5.61) | 92.33 |  | 11964 (2.38) | 39.16 |  | 0.46 (0.45-0.47) |
| Anxiety | 8677 (1.73) | 6723 (1.34) | 77.48 |  | 3754 (0.75) | 43.26 |  | 0.34 (0.33-0.35) |
| Schizophrenia | 2489 (0.5) | 1995 (0.4) | 80.15 |  | 1820 (0.36) | 73.12 |  | 0.69 (0.68-0.71) |
| Alcohol problems | 2818 (0.56) | 811 (0.16) | 28.78 |  | 2436 (0.48) | 86.44 |  | 0.26 (0.24-0.28) |
| Psychoactive substance abuse | 1311 (0.26) | 98 (0.02) | 7.48 |  | 1241 (0.25) | 94.66 |  | 0.04 (0.03-0.06) |
| Dementia | 433 (0.09) | 124 (0.02) | 28.64 |  | 385 (0.08) | 88.91 |  | 0.3 (0.25-0.35) |
| Parkinson’s disease | 963 (0.19) | 846 (0.17) | 87.85 |  | 857 (0.17) | 88.99 |  | 0.87 (0.85-0.89) |
| Epilepsy | 4821 (0.96) | 3736 (0.74) | 77.49 |  | 4053 (0.81) | 84.07 |  | 0.76 (0.75-0.77) |
| Migraine | 15427 (3.07) | 14383 (2.86) | 93.23 |  | 3043 (0.61) | 19.73 |  | 0.22 (0.21-0.23) |
| Painful conditions | 68108 (13.55) | 49900 (9.93) | 73.27 |  | 32844 (6.54) | 48.22 |  | 0.3 (0.29-0.3) |
| Chronic fatigue syndrome | 2260 (0.45) | 2168 (0.43) | 95.93 |  | 704 (0.14) | 31.15 |  | 0.43 (0.4-0.45) |
| Connective tissue disorders | 12782 (2.54) | 11026 (2.19) | 86.26 |  | 8193 (1.63) | 64.10 |  | 0.66 (0.66-0.67) |
| Osteoporosis | 48886 (9.73) | 40686 (8.1) | 83.23 |  | 24555 (4.89) | 50.23 |  | 0.47 (0.46-0.47) |
| Fracture | 2219 (0.44) | 808 (0.16) | 36.41 |  | 1586 (0.32) | 71.47 |  | 0.14 (0.13-0.16) |
| Anorexia | 396 (0.08) | 370 (0.07) | 93.43 |  | 83 (0.02) | 20.96 |  | 0.25 (0.2-0.3) |
| Dyspepsia | 58226 (11.59) | 39062 (7.77) | 67.09 |  | 42228 (8.4) | 72.52 |  | 0.53 (0.53-0.53) |
| Treated constipation | 5368 (1.07) | 403 (0.08) | 7.51 |  | 5065 (1.01) | 94.36 |  | 0.04 (0.03-0.04) |
| Pernicious anaemia | 1681 (0.33) | 1517 (0.3) | 90.24 |  | 750 (0.15) | 44.62 |  | 0.52 (0.49-0.54) |
| Diverticular disease | 13247 (2.64) | 5402 (1.08) | 40.78 |  | 11706 (2.33) | 88.37 |  | 0.44 (0.43-0.45) |
| Chronic kidney disease | 2932 (0.58) | 1310 (0.26) | 44.68 |  | 2569 (0.51) | 87.62 |  | 0.49 (0.47-0.51) |
| Inflammatory bowel disease | 5190 (1.03) | 4231 (0.84) | 81.52 |  | 4297 (0.86) | 82.79 |  | 0.78 (0.77-0.79) |
| Irritable bowel syndrome | 13699 (2.73) | 11490 (2.29) | 83.87 |  | 5276 (1.05) | 38.51 |  | 0.36 (0.35-0.37) |
| Viral hepatitis | 1589 (0.32) | 1336 (0.27) | 84.08 |  | 544 (0.11) | 34.24 |  | 0.31 (0.28-0.34) |
| Chronic liver disease | 3790 (0.75) | 971 (0.19) | 25.62 |  | 3177 (0.63) | 83.83 |  | 0.17 (0.15-0.19) |
| Thyroid disorders | 31138 (6.2) | 29129 (5.8) | 93.55 |  | 21005 (4.18) | 67.46 |  | 0.75 (0.74-0.75) |
| Psoriasis/eczema | 19340 (3.85) | 17833 (3.55) | 92.21 |  | 4510 (0.9) | 23.32 |  | 0.26 (0.25-0.27) |
| Chronic sinusitis | 4775 (0.95) | 3102 (0.62) | 64.96 |  | 2015 (0.4) | 42.20 |  | 0.13 (0.12-0.14) |
| Meniere’s disease | 1769 (0.35) | 1376 (0.27) | 77.78 |  | 857 (0.17) | 48.45 |  | 0.41 (0.39-0.44) |
| HIV | 486 (0.1) | 471 (0.09) | 96.91 |  | 207 (0.04) | 42.59 |  | 0.57 (0.52-0.61) |
| Endometriosis | 6946 (1.38) | 4057 (0.81) | 58.41 |  | 3900 (0.78) | 56.15 |  | 0.25 (0.24-0.26) |
| Polycystic ovary | 755 (0.15) | 623 (0.12) | 82.52 |  | 271 (0.05) | 35.89 |  | 0.31 (0.27-0.35) |
| Prostate disorders | 12186 (2.43) | 8256 (1.64) | 67.75 |  | 8718 (1.73) | 71.54 |  | 0.56 (0.55-0.57) |
| Glaucoma | 6073 (1.21) | 5313 (1.06) | 87.49 |  | 3976 (0.79) | 65.47 |  | 0.69 (0.68-0.7) |
| Cataract | 12986 (2.58) | 7312 (1.46) | 56.31 |  | 9996 (1.99) | 76.98 |  | 0.49 (0.48-0.5) |
| AMD | 1019 (0.2) | 296 (0.06) | 29.05 |  | 838 (0.17) | 82.24 |  | 0.2 (0.17-0.23) |
| Lung cancer | 465 (0.09) | 303 (0.06) | 65.16 |  | 337 (0.07) | 72.47 |  | 0.55 (0.5-0.59) |
| Skin cancer | 5114 (1.02) | 693 (0.14) | 13.55 |  | 4666 (0.93) | 91.24 |  | 0.09 (0.08-0.1) |
| Melanoma | 4159 (0.83) | 3709 (0.74) | 89.18 |  | 1475 (0.29) | 35.47 |  | 0.39 (0.38-0.41) |
| Stomach cancer | 339 (0.07) | 185 (0.04) | 54.57 |  | 239 (0.05) | 70.50 |  | 0.4 (0.34-0.46) |
| Oesophageal cancer | 1730 (0.34) | 212 (0.04) | 12.25 |  | 1684 (0.34) | 97.34 |  | 0.17 (0.15-0.2) |
| Colon cancer | 2168 (0.43) | 1575 (0.31) | 72.65 |  | 1413 (0.28) | 65.18 |  | 0.55 (0.53-0.57) |
| Rectal cancer | 1035 (0.21) | 350 (0.07) | 33.82 |  | 890 (0.18) | 85.99 |  | 0.33 (0.3-0.36) |
| Uterine cancer | 1479 (0.29) | 1226 (0.24) | 82.89 |  | 306 (0.06) | 20.69 |  | 0.07 (0.05-0.09) |
| Ovarian cancer | 968 (0.19) | 837 (0.17) | 86.47 |  | 507 (0.1) | 52.38 |  | 0.56 (0.53-0.59) |
| Breast cancer | 11490 (2.29) | 11313 (2.25) | 98.46 |  | 7696 (1.53) | 66.98 |  | 0.79 (0.78-0.79) |
| Prostate cancer | 3616 (0.72) | 3464 (0.69) | 95.8 |  | 2861 (0.57) | 79.12 |  | 0.86 (0.85-0.87) |
| Other cancers | 25865 (5.15) | 18028 (3.59) | 69.7 |  | 14821 (2.95) | 57.30 |  | 0.41 (0.4-0.41) |

*Kappa statistic for the rates of the self-reported and inpatient data was estimated using PROC FREQ.
