# Supplementary material for: Temporal trajectories of important diseases in the life course and premature mortality in the UK Biobank
Source: BMC Med. 2022 May 27;20:185. doi: 10.1186/s12916-022-02384-3 (PMC9137080; doi:10.1186/s12916-022-02384-3)
Supplement: Supplementary file 2 — Additional file 2: Figure S1. Proportional hazards assumption test for cardiometabolic disorders and incident mortality. Figure S2. Proportional hazards assumption test for cardiometabolic disorders and incident mortality. Figure S3. Proportional hazards assumption test for cancers and incident mortality. Figure S4. Proportional hazards assumption test for musculoskeletal disorders, digestive disorders and incident mortality. Figure S5. Proportional hazards assumption test for other diseases and incident mortality. [file 12916_2022_2384_MOESM2_ESM.doc]

**Additional file 2**

**Figure S1. Proportional hazards assumption test for cardiometabolic disorders and incident mortality**

**Figure S2. Proportional hazards assumption test for mental and neurogenerative disorders and incident mortality**

**Figure S3. Proportional hazards assumption test for cancers and incident mortality**

**Figure S4. Proportional hazards assumption test for musculoskeletal and digestive disorders and incident mortality**

**Figure S5. Proportional hazards assumption test for other diseases and incident mortality**


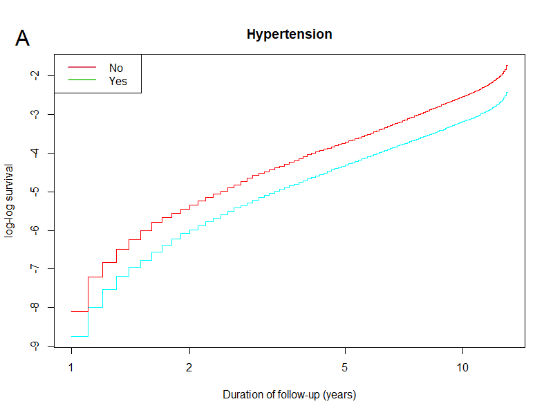

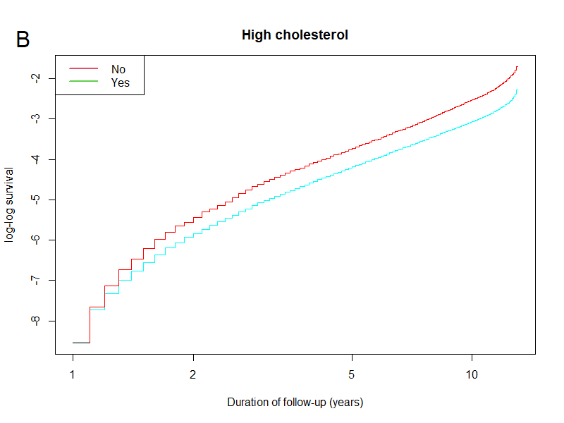

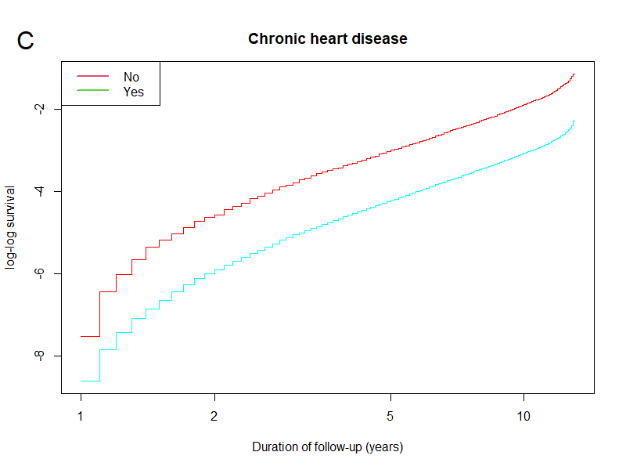

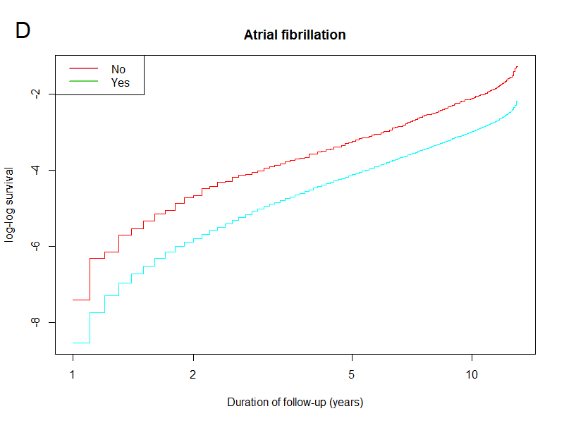

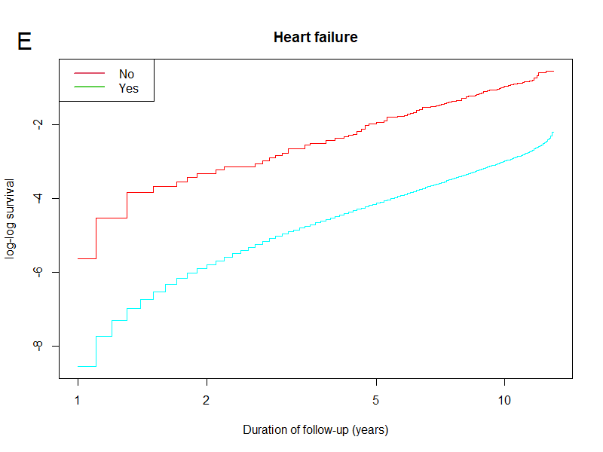

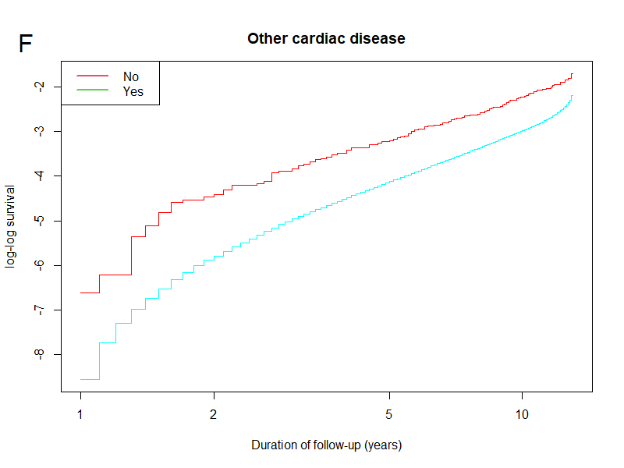

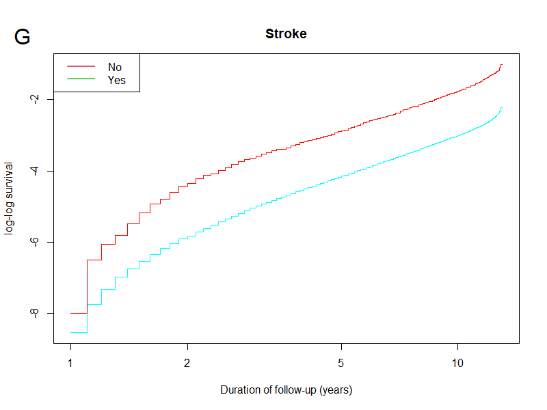

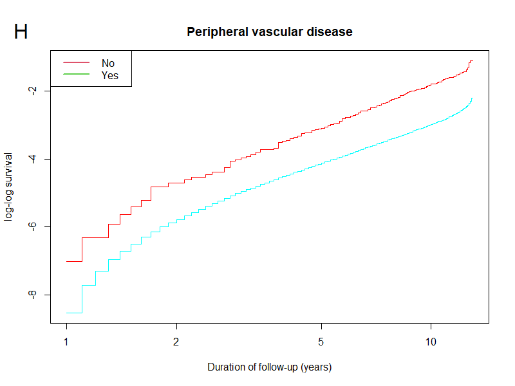

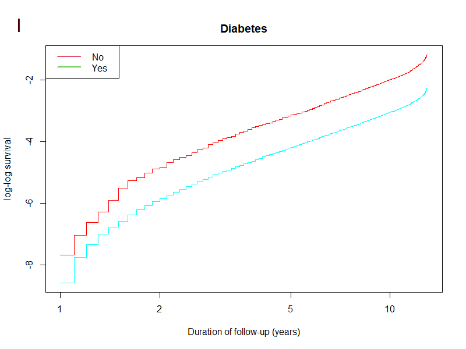


**Figure S1. Proportional hazards assumption test for cardiometabolic disorders and incident mortality**

Log-minus-log plot was used to test proportional hazards assumption.


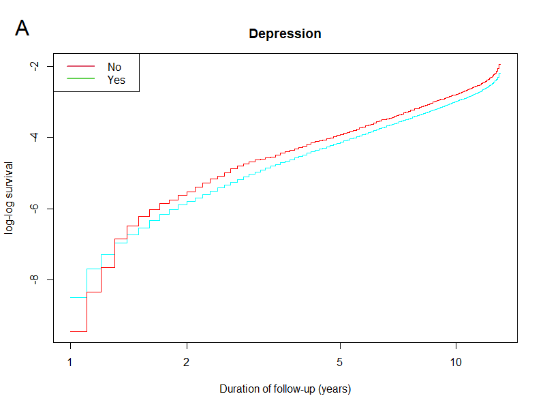

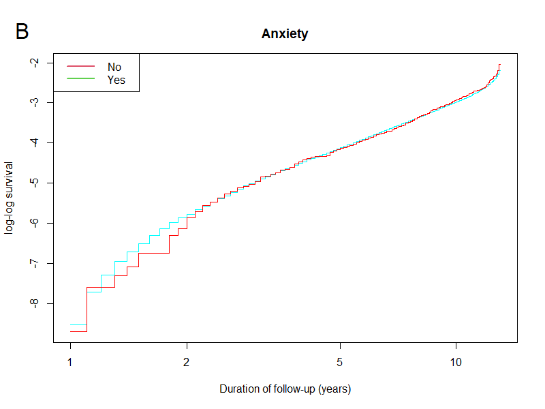

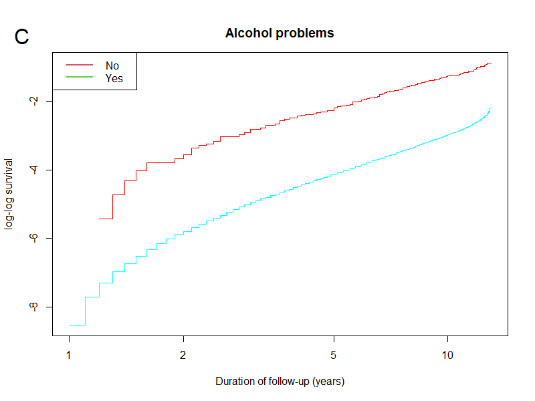


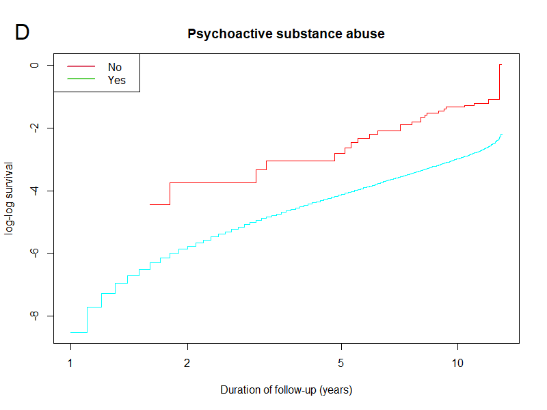

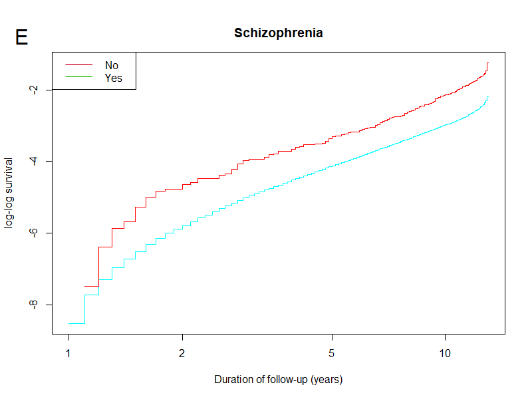

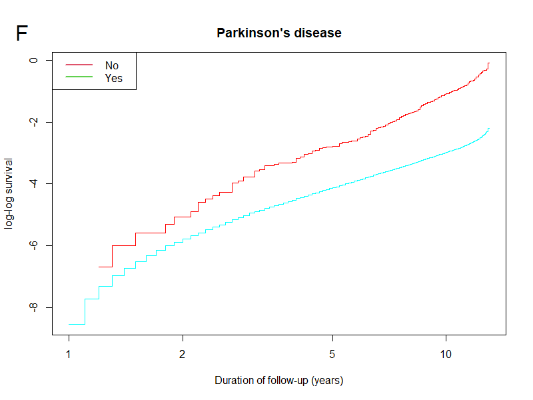


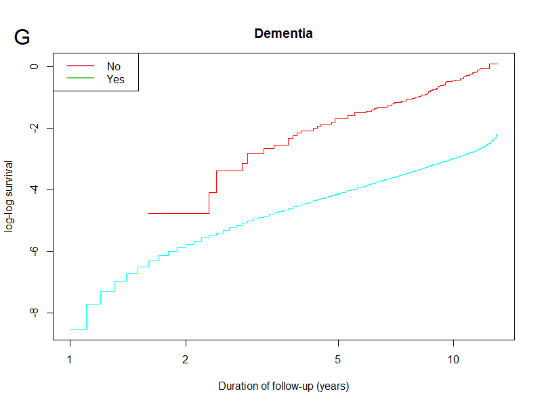

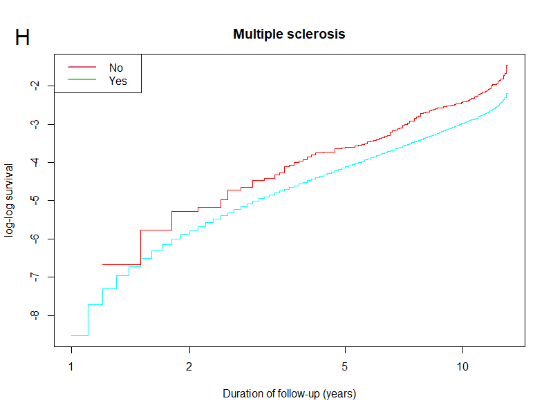

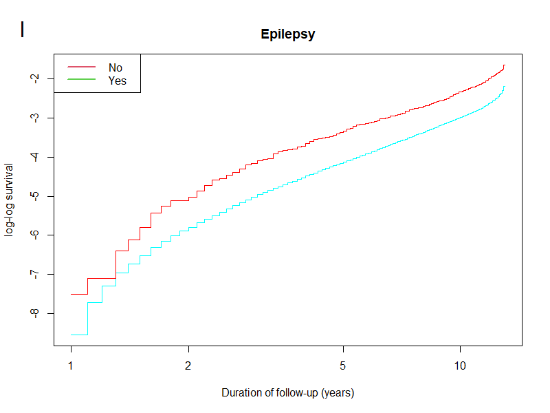


**Figure S2. Proportional hazards assumption test for mental and neurogenerative disorders and incident mortality**

Log-minus-log plot was used to test proportional hazards assumption.


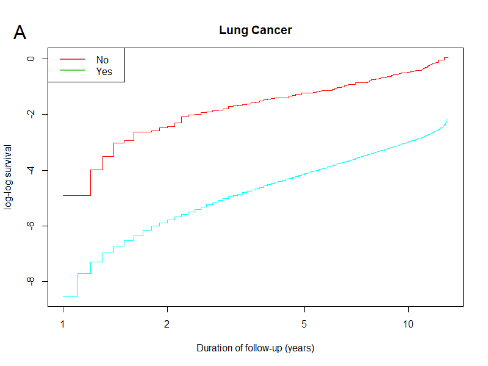

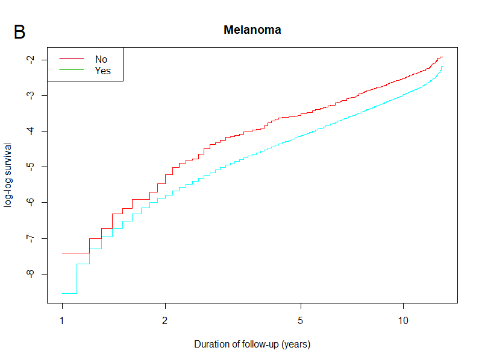

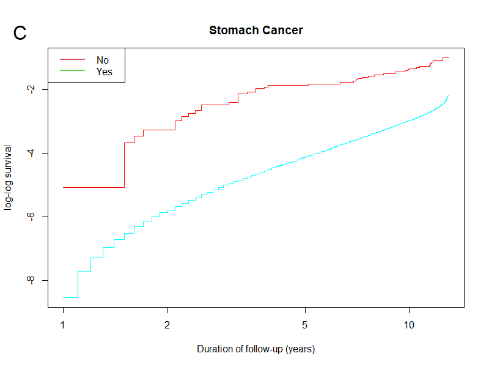


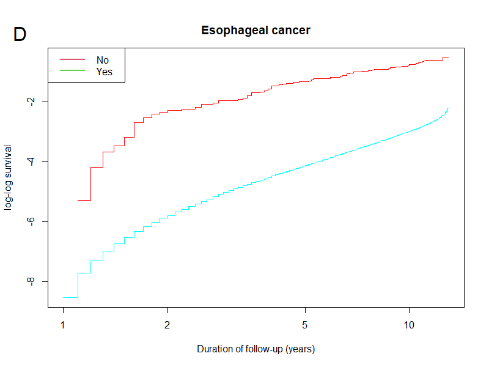

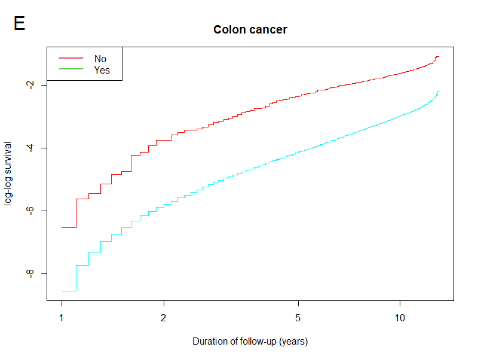

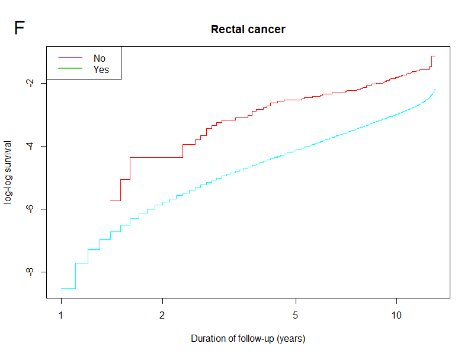

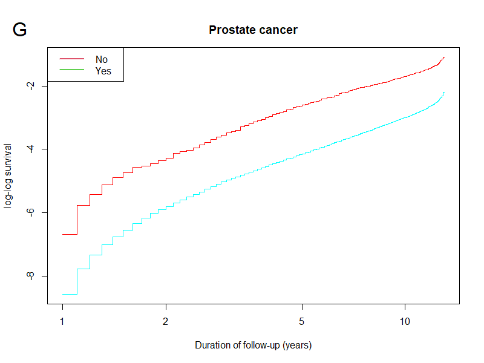

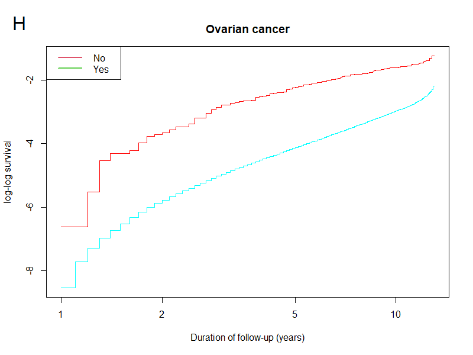

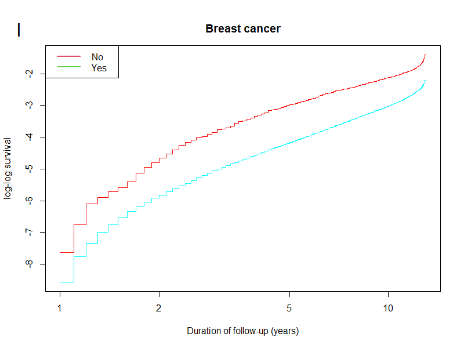

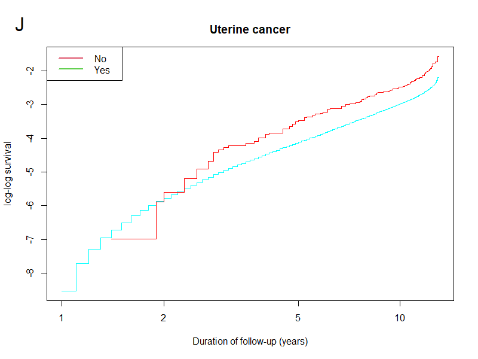

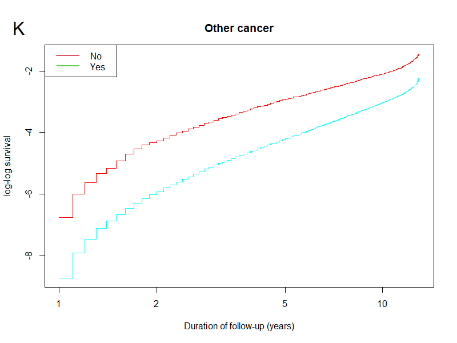


**Figure S3. Proportional hazards assumption test for cancers and incident mortality**

Log-minus-log plot was used to test proportional hazards assumption.


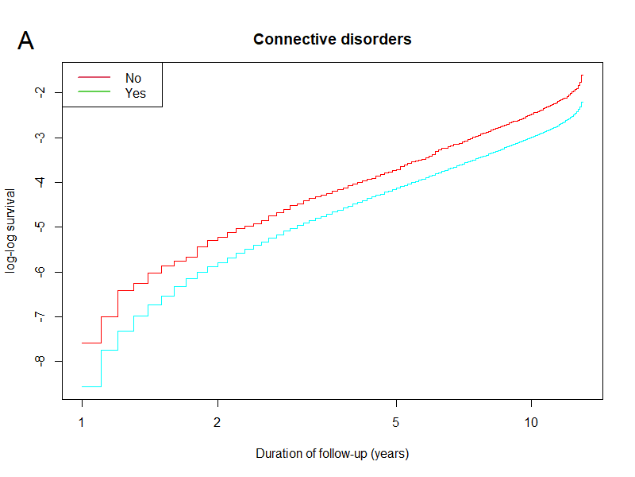

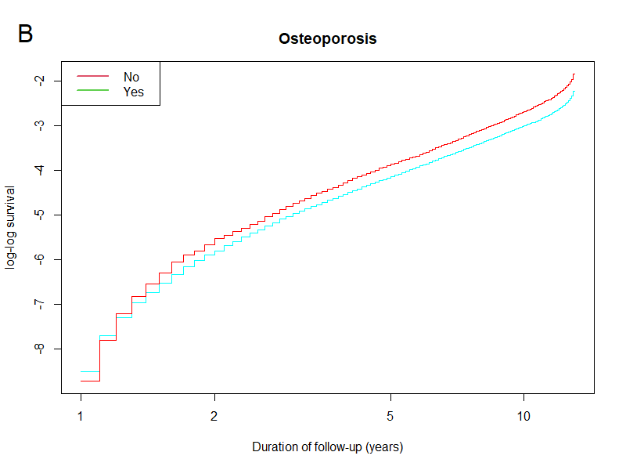

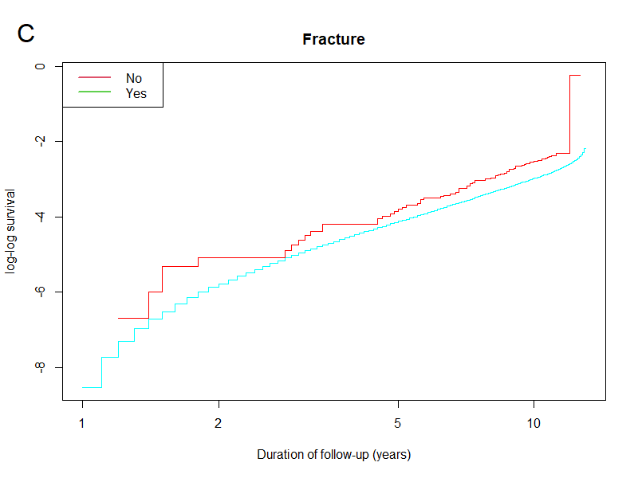

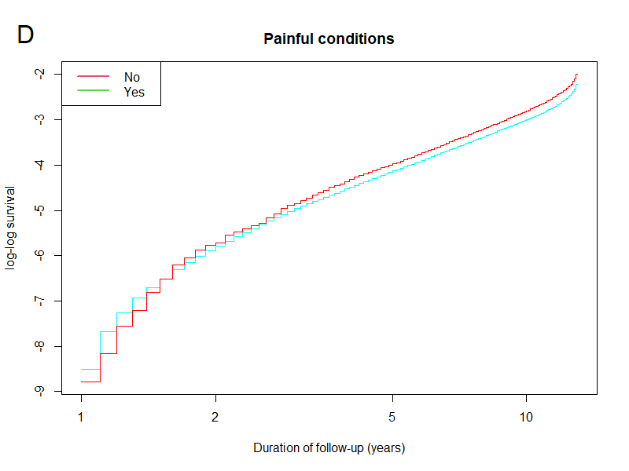

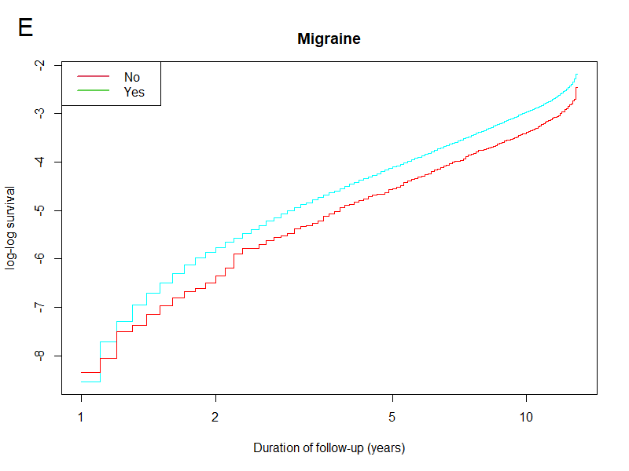

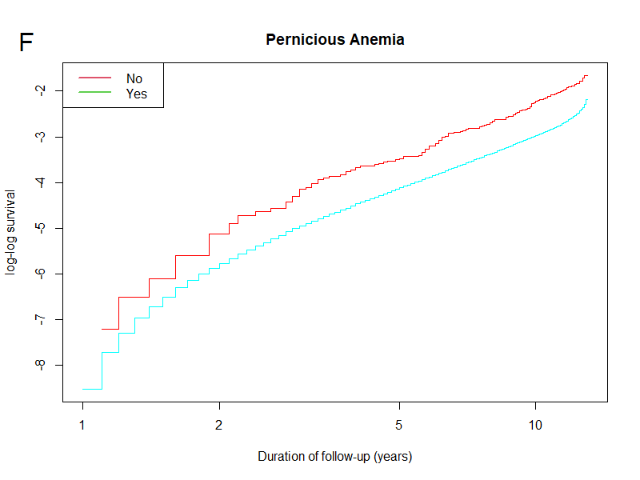

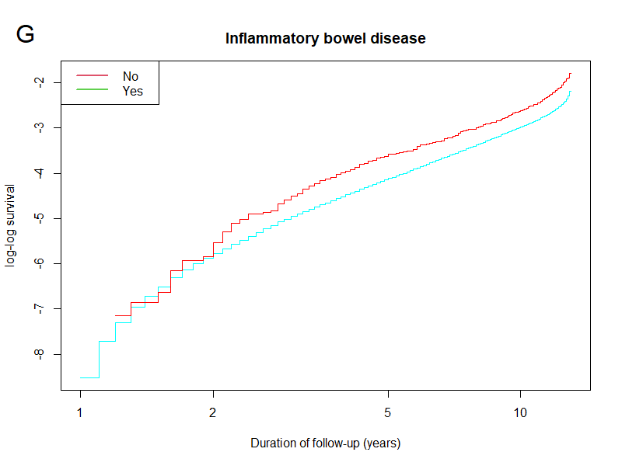

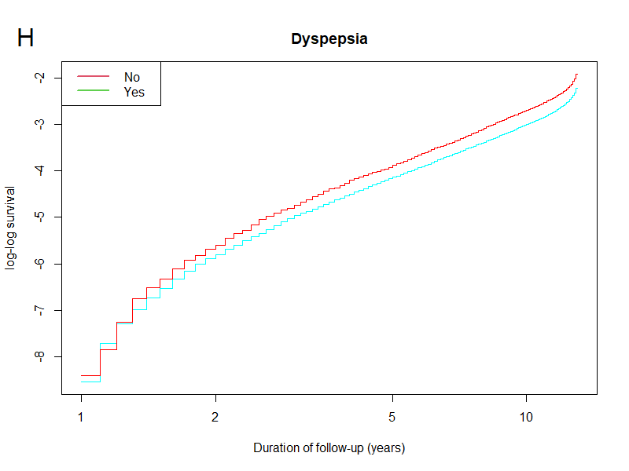

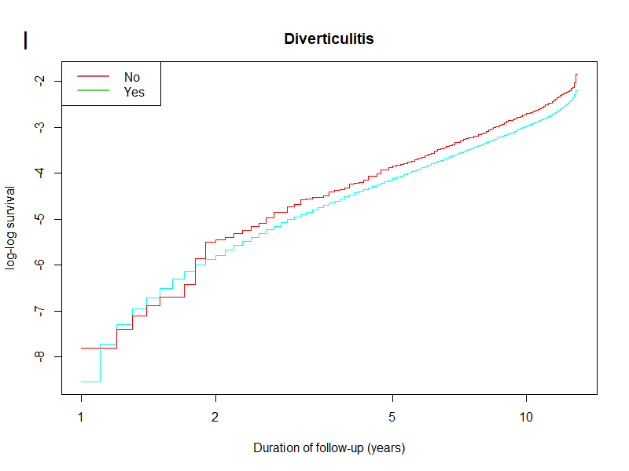


**Figure S4. Proportional hazards assumption test for musculoskeletal and digestive disorders and incident mortality**

Log-minus-log plot was used to test proportional hazards assumption.


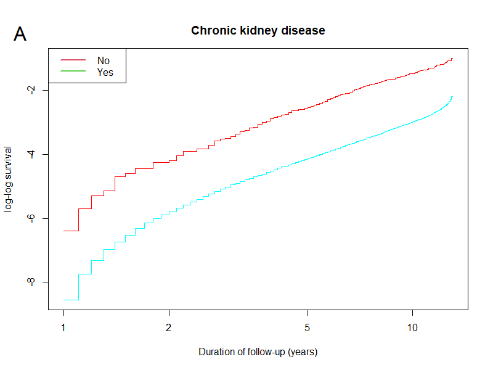

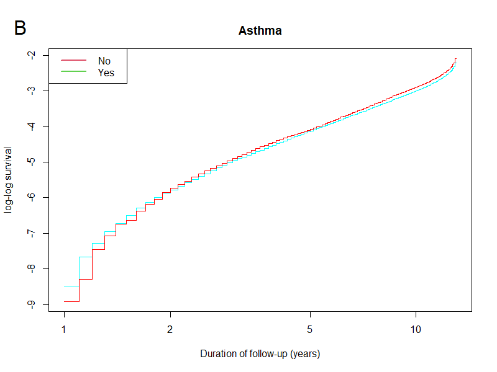

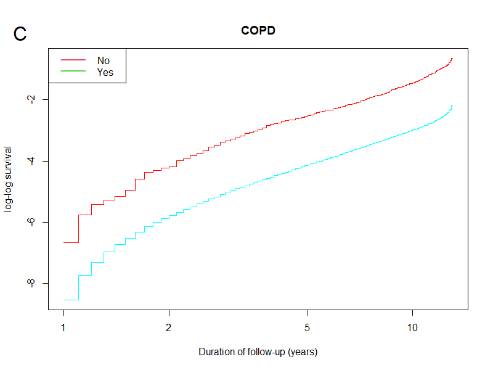


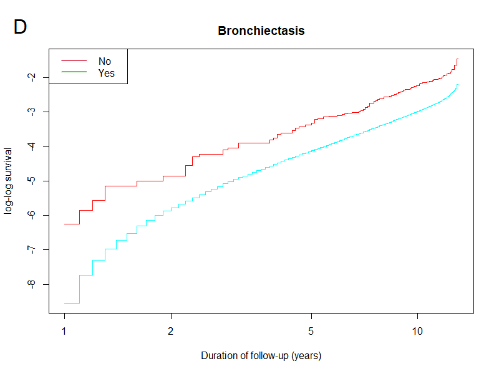

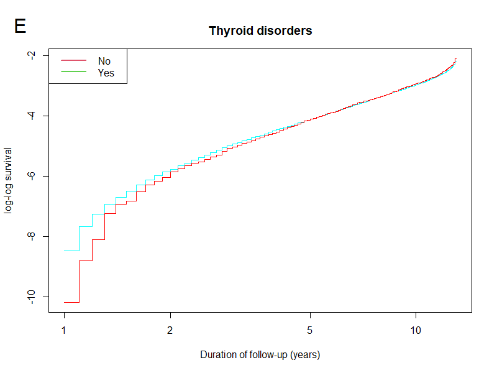

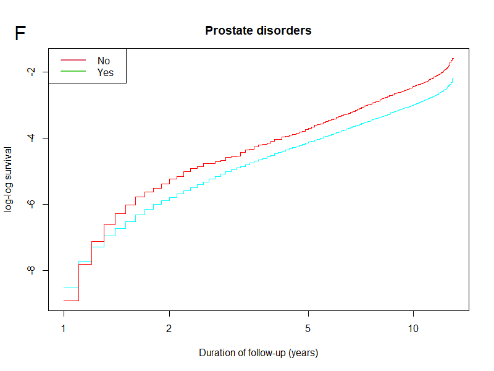

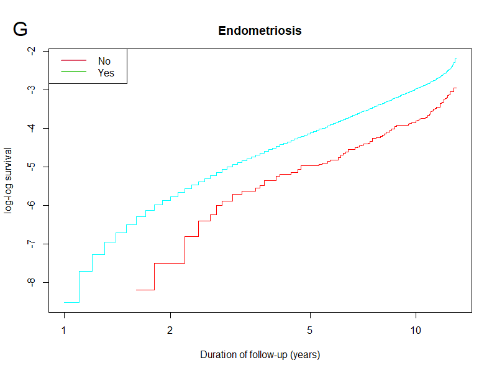

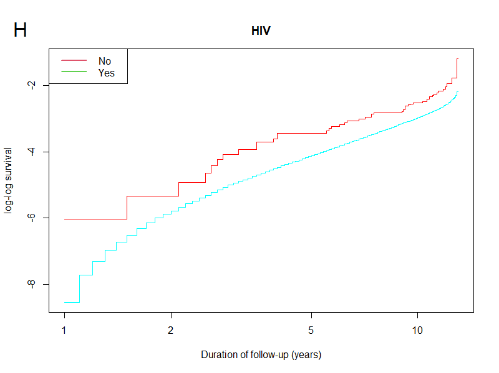

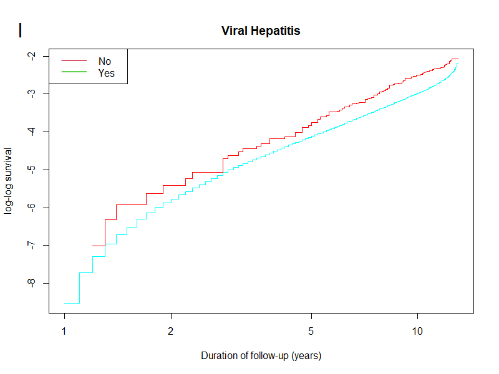

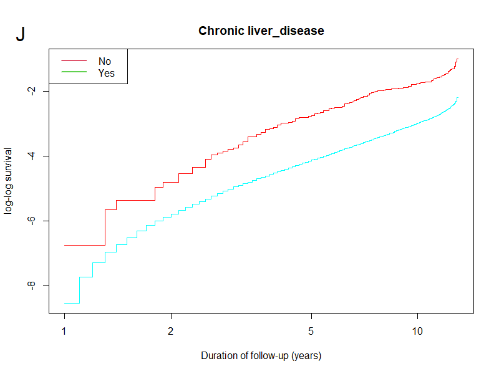

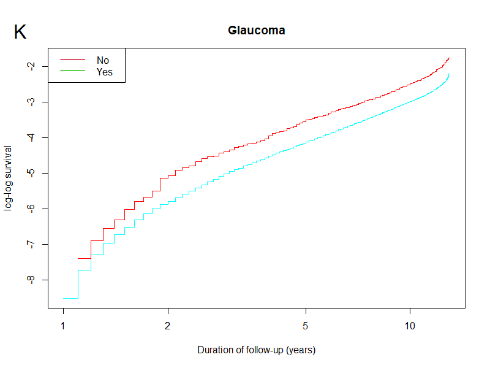

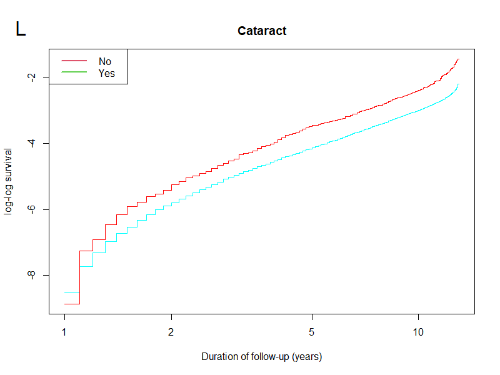


**Figure S5. Proportional hazards assumption test for other diseases and incident mortality**

COPD, chronic obstructive pulmonary disease; HIV, human immunodeficiency virus

Log-minus-log plot was used to test proportional hazards assumption.
