# Supplementary material for: Temporal trajectories of important diseases in the life course and premature mortality in the UK Biobank
Source: BMC Med. 2022 May 27;20:185. doi: 10.1186/s12916-022-02384-3 (PMC9137080; doi:10.1186/s12916-022-02384-3)
Supplement: Supplementary file 3 — Additional file 3: Figure S6. Risk for mortality associated with individual diseases of interest in life-course. Figure S7. Disease trajectory in life-course among individuals who were diagnosed with two diseases before dying from any reasons. Figure S8. Disease trajectory in life-course among individuals who were diagnosed with three diseases before dying from any reasons. Figure S9. Disease trajectory in life-course among individuals who were diagnosed with four diseases before dying from any reasons. Figure S10. Disease trajectory in life-course among individuals who were diagnosed with five diseases before dying from any reasons. Figure S11. Disease trajectory in life-course among individuals who were diagnosed with six or more diseases before dying from any reasons. [file 12916_2022_2384_MOESM3_ESM.docx]

**Additional file 3**

**Figure S6. Risk for mortality associated with individual diseases of interest in life-course**

**Figure S7. Disease trajectory in life-course among individuals who were diagnosed with two diseases before dying from any reasons**

**Figure S8. Disease trajectory in life-course among individuals who were diagnosed with three diseases before dying from any reasons**

**Figure S9. Disease trajectory in life-course among individuals who were diagnosed with four diseases before dying from any reasons**

**Figure S10. Disease trajectory in life-course among individuals who were diagnosed with five diseases before dying from any reasons**

**Figure S11. Disease trajectory in life-course among individuals who were diagnosed with six or more diseases before dying from any reasons**


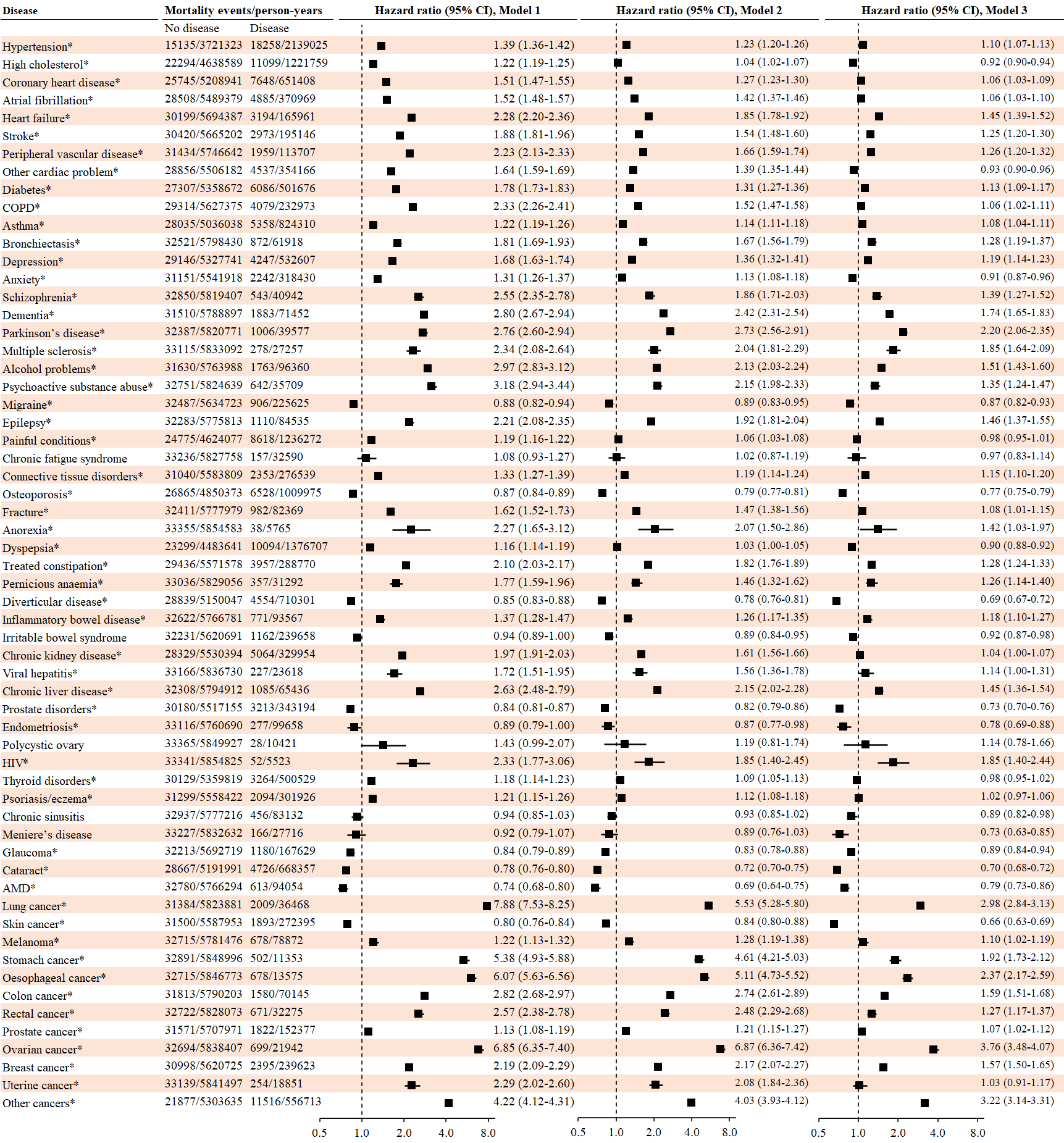


**Figure S6. Risk for mortality associated with individual diseases of interest in life-course**

Cox proportional hazard regression models were used to examine the association between each of the 60 major diseases in life-course (including those diagnosed during follow-up before mortality) and incident mortality. Model 1 was adjusted for age and gender; Model 2 was adjusted for Model 1 plus ethnicity, education, income, BMI, smoking, physical acidity, alcohol consumption, sleep duration, diet, blood pressure, HDL-C, triglycerides, and HbA1c. Model 3 was adjusted for Model 2 plus all other 59 chronic diseases. The analysis for breast cancer, ovarian cancer, endometriosis, and polycystic ovary was conducted among women only while the analysis for prostate cancer and prostate disorders was conducted among men only. Individuals with disease occurred in the last year before mortality were excluded from the analysis.

*Refers to significant associations after adjustment for false discovery rate at a 5% level using Benjamin-Hochberg's procedure.


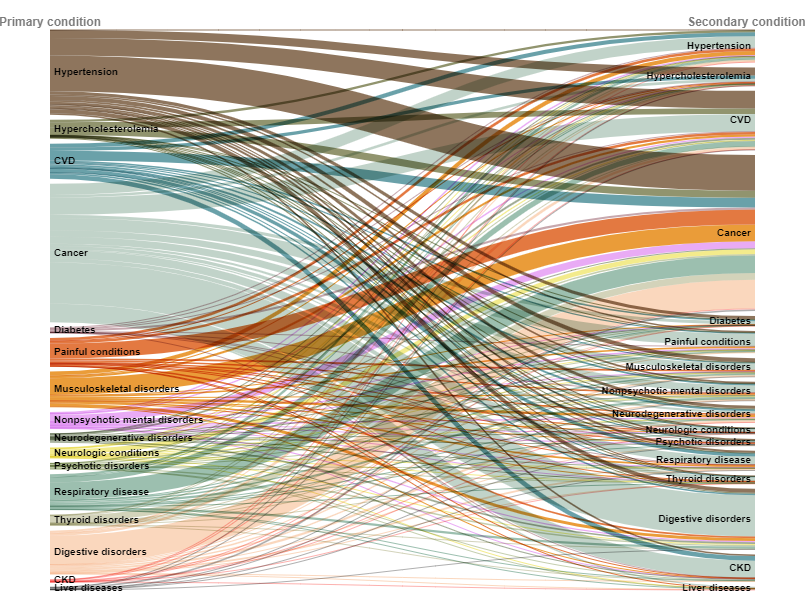


**Figure S7. Disease trajectory in life-course among individuals who were diagnosed with two diseases before dying from any reasons**

Disease trajectory was computed based on the permutation of 16 groups of diseases according to the age at diagnosis of the diseases. Primary disease is the first one of diseases of interest diagnosed in life-course.


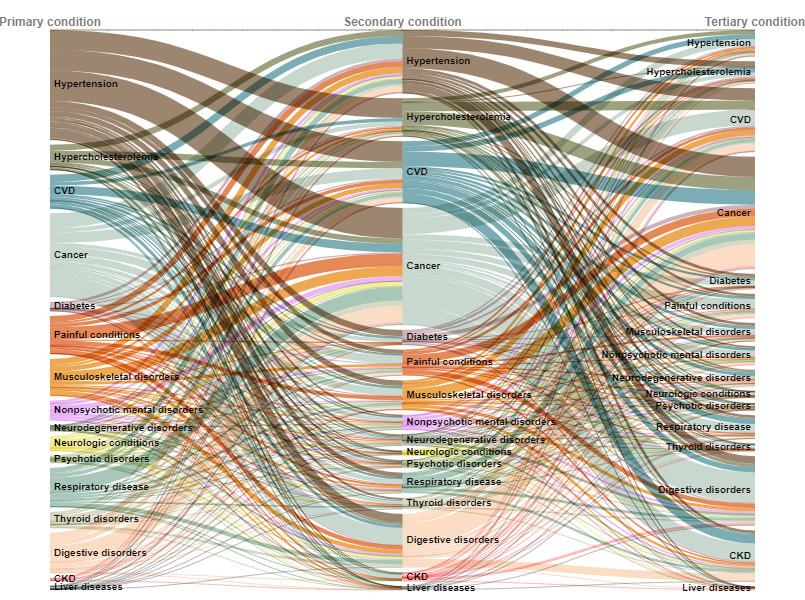


**Figure S8. Disease trajectory in life-course among individuals who were diagnosed with three diseases before dying from any reasons**

Disease trajectory was computed based on the permutation of 16 groups of diseases according to the age at diagnosis of the diseases. Primary disease is the first one of diseases of interest diagnosed in life-course.


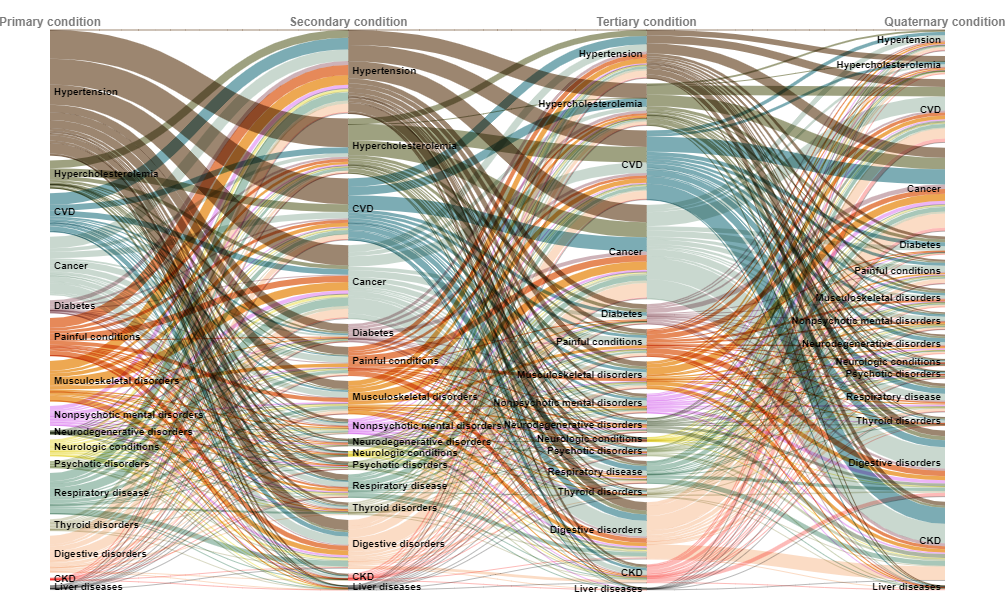


**Figure S9. Disease trajectory in life-course among individuals who were diagnosed with four diseases before dying from any reasons**

Disease trajectory was computed based on the permutation of 16 groups of diseases according to the age at diagnosis of the diseases. Primary disease is the first one of diseases of interest diagnosed in life-course.


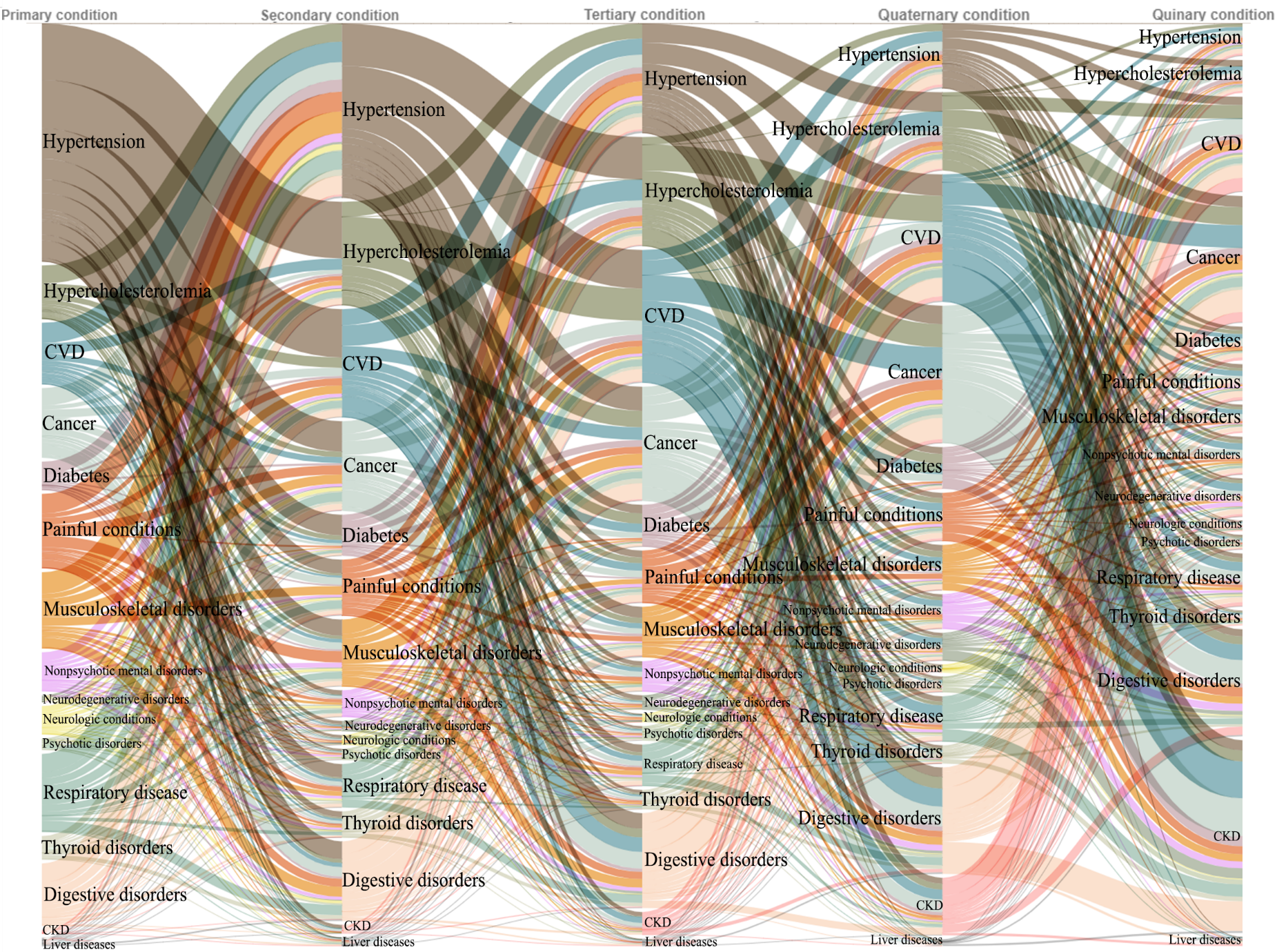


**Figure S10. Disease trajectory in life-course among individuals who were diagnosed with five diseases before dying from any reasons**

Disease trajectory was computed based on the permutation of 16 groups of diseases according to the age at diagnosis of the diseases. Primary disease is the first one of diseases of interest diagnosed in life-course.


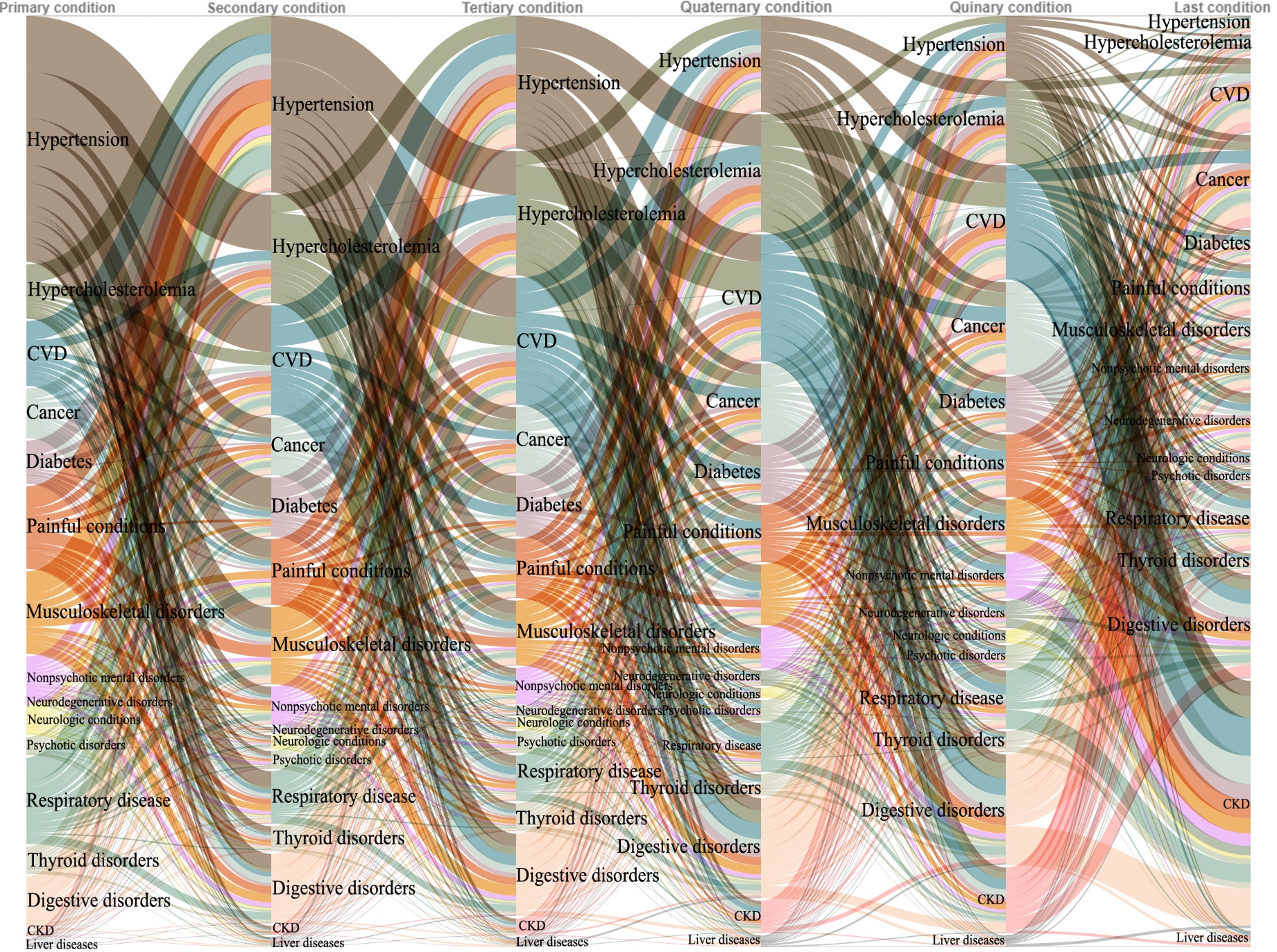


**Figure S11. Disease trajectory in life-course among individuals who were diagnosed with six or more diseases before dying from any reasons**

Disease trajectory was computed based on the permutation of 16 groups of diseases according to the age at diagnosis of the diseases. Primary disease is the first one of diseases of interest diagnosed in life-course and last disease is the last one diagnosed before mortality. Diseases diagnosed from seventh until the last one were not taken into consideration among those who were diagnosed with seven or more diseases.
