# Supplementary material for: Temporal trajectories of important diseases in the life course and premature mortality in the UK Biobank
Source: BMC Med. 2022 May 27;20:185. doi: 10.1186/s12916-022-02384-3 (PMC9137080; doi:10.1186/s12916-022-02384-3)
Supplement: Supplementary file 4 — Additional file 4: Figure S12. Disease trajectory in the whole life-course among individuals who were diagnosed with two diseases before dying from cancer. Figure S13. Disease trajectory in the whole life-course among individuals who were diagnosed with three diseases before dying from cancer. Figure S14. Disease trajectory in the whole life-course among individuals who were diagnosed with four diseases before dying from cancer. Figure S15. Disease trajectory in the whole life-course among individuals who were diagnosed with five diseases before dying from cancer. Figure S16. Disease trajectory in the whole life-course among individuals who were diagnosed with six or more diseases before dying from cancer. Figure S17. Disease trajectory in the whole life-course among individuals who were diagnosed with two diseases before dying from cardiovascular disease. Figure S18. Disease trajectory in the whole life-course among individuals who were diagnosed with three diseases before dying from cardiovascular disease. Figure S19. Disease trajectory in the whole life-course among individuals who were diagnosed with four diseases before dying from cardiovascular disease. Figure S20. Disease trajectory in the whole life-course among individuals who were diagnosed with five diseases before dying from cardiovascular disease. Figure S21. Disease trajectory in the whole life-course among individuals who were diagnosed with six or more diseases before dying from cardiovascular disease. [file 12916_2022_2384_MOESM4_ESM.docx]

**Additional file 4**

**Figure S12. Disease trajectory in the whole life-course among individuals who were diagnosed with two diseases before dying from cancer**

**Figure S13. Disease trajectory in the whole life-course among individuals who were diagnosed with three diseases before dying from cancer**

**Figure S14. Disease trajectory in the whole life-course among individuals who were diagnosed with four diseases before dying from cancer**

**Figure S15. Disease trajectory in the whole life-course among individuals who were diagnosed with five diseases before dying from cancer**

**Figure S16. Disease trajectory in the whole life-course among individuals who were diagnosed with six or more diseases before dying from cancer**

**Figure S17. Disease trajectory in the whole life-course among individuals who were diagnosed with two diseases before dying from cardiovascular disease**

**Figure S18. Disease trajectory in the whole life-course among individuals who were diagnosed with three diseases before dying from cardiovascular disease**

**Figure S19. Disease trajectory in the whole life-course among individuals who were diagnosed with four diseases before dying from cardiovascular disease**

**Figure S20. Disease trajectory in the whole life-course among individuals who were diagnosed with five diseases before dying from cardiovascular disease**

**Figure S21. Disease trajectory in the whole life-course among individuals who were diagnosed with six or more diseases before dying from cardiovascular disease**


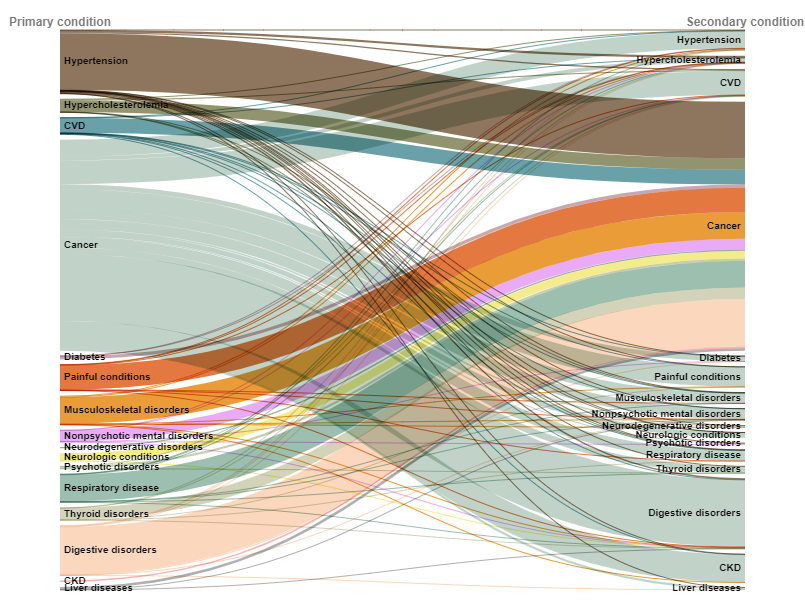


**Figure S12. Disease trajectory in the whole life-course among individuals who were diagnosed with two diseases before dying from cancer**

Disease trajectory was computed based on the permutation of 16 groups of diseases according to the age at diagnosis of the diseases. Primary disease is the first one of diseases of interest diagnosed in life-course.


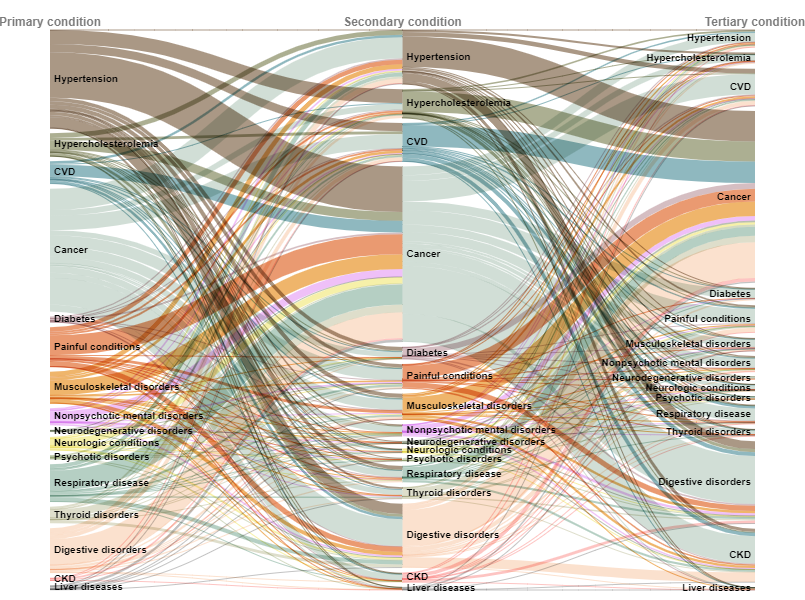


**Figure S13. Disease trajectory in the whole life-course among individuals who were diagnosed with three diseases before dying from cancer**

Disease trajectory was computed based on the permutation of 16 groups of diseases according to the age at diagnosis of the diseases. Primary disease is the first one of diseases of interest diagnosed in life-course.


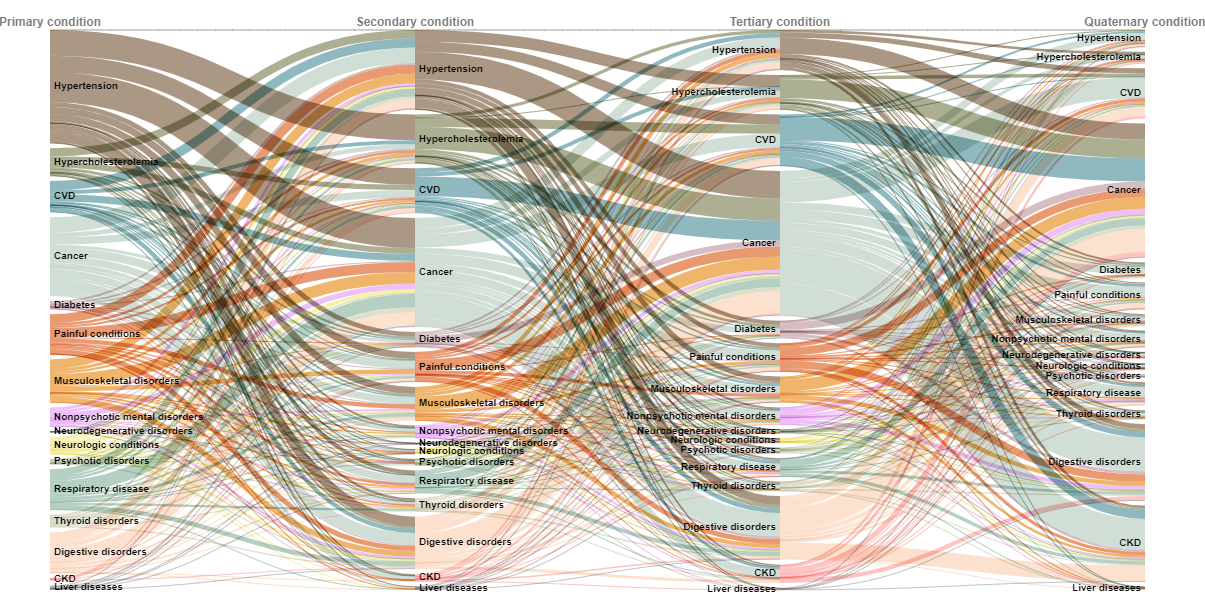


**Figure S14. Disease trajectory in the whole life-course among individuals who were diagnosed with four diseases before dying from cancer**

Disease trajectory was computed based on the permutation of 16 groups of diseases according to the age at diagnosis of the diseases. Primary disease is the first one of diseases of interest diagnosed in life-course.


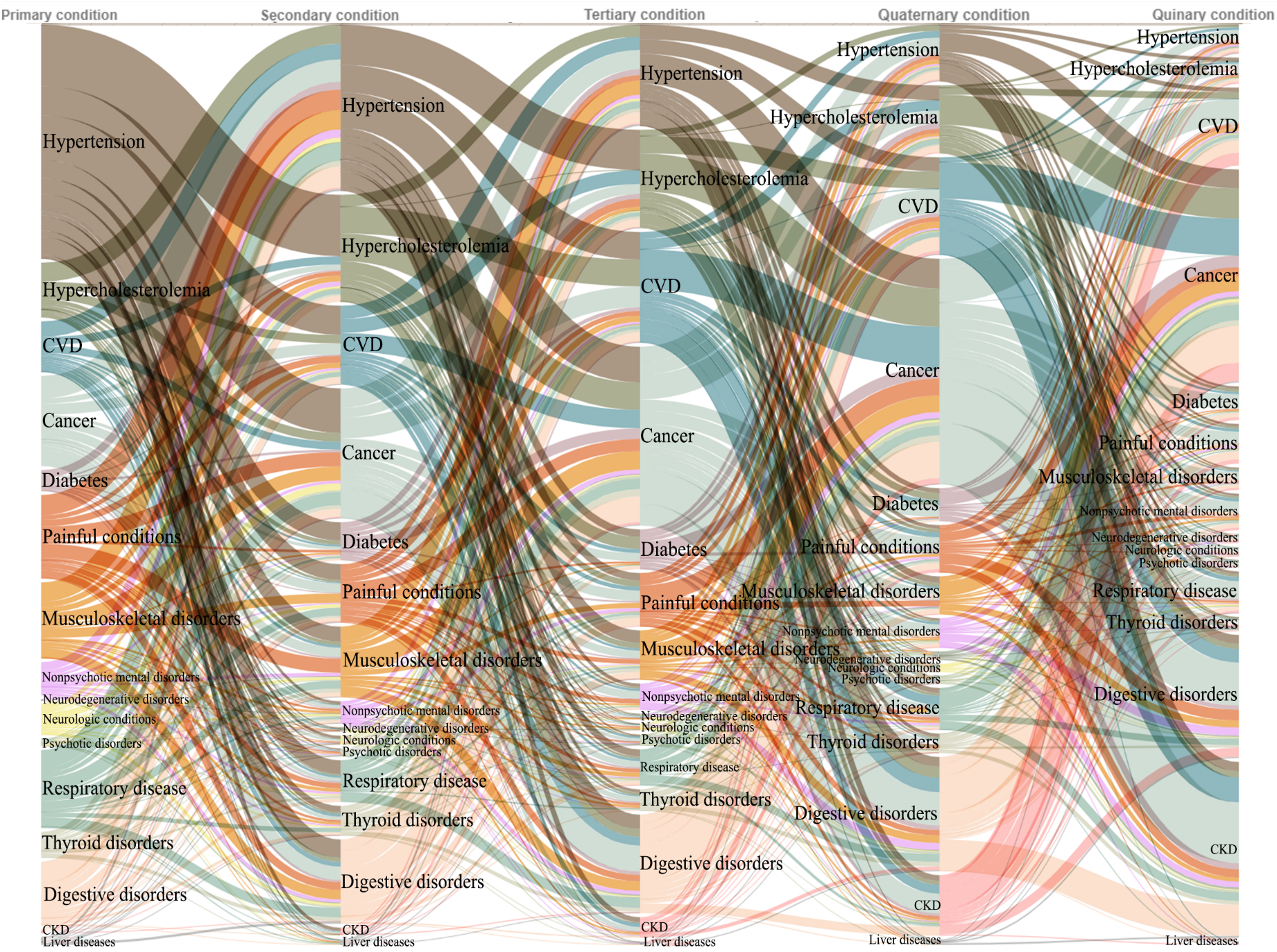


**Figure S15. Disease trajectory in the whole life-course among individuals who were diagnosed with five diseases before dying from cancer**

Disease trajectory was computed based on the permutation of 16 groups of diseases according to the age at diagnosis of the diseases. Primary disease is the first one of diseases of interest diagnosed in life-course.


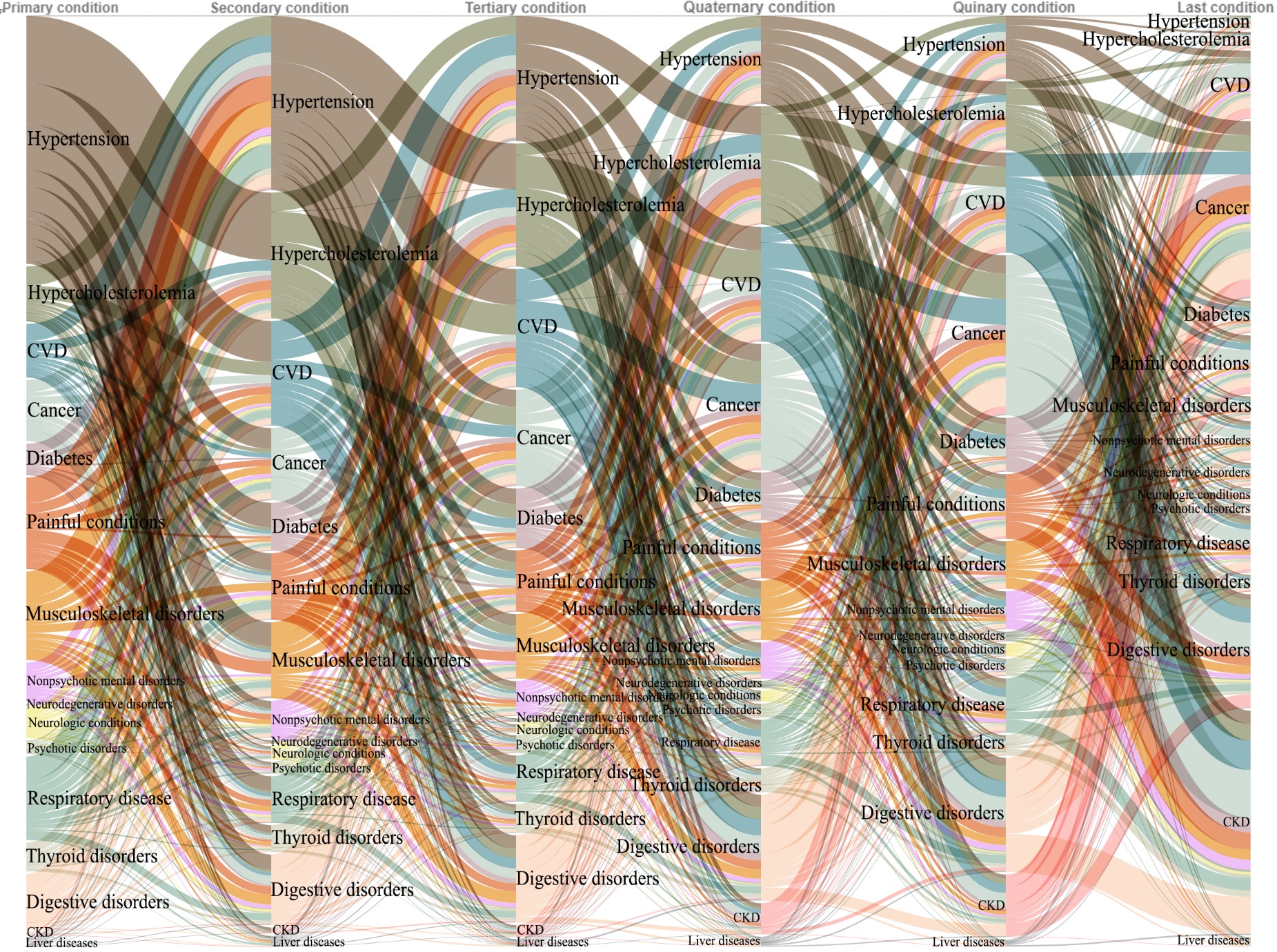


**Figure S16. Disease trajectory in the whole life-course among individuals who were diagnosed with six or more diseases before dying from cancer**

Disease trajectory was computed based on the permutation of 16 groups of diseases according to the age at diagnosis of the diseases. Primary disease is the first one of diseases of interest diagnosed in life-course and last disease is the last one diagnosed before mortality. Diseases diagnosed from seventh until the last one were not taken into consideration among those who were diagnosed with seven or more diseases.


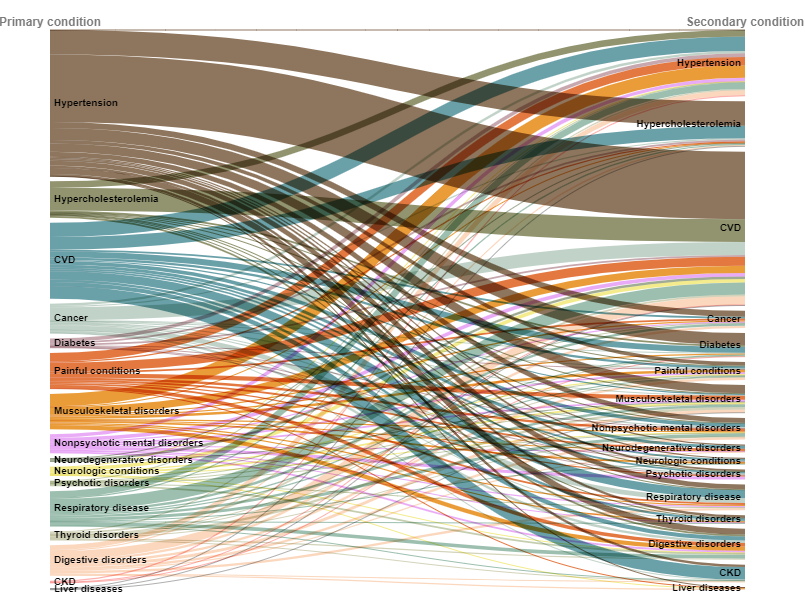


**Figure S17. Disease trajectory in the whole life-course among individuals who were diagnosed with two diseases before dying from cardiovascular disease**

Disease trajectory was computed based on the permutation of 16 groups of diseases according to the age at diagnosis of the diseases. Primary disease is the first one of diseases of interest diagnosed in life-course.


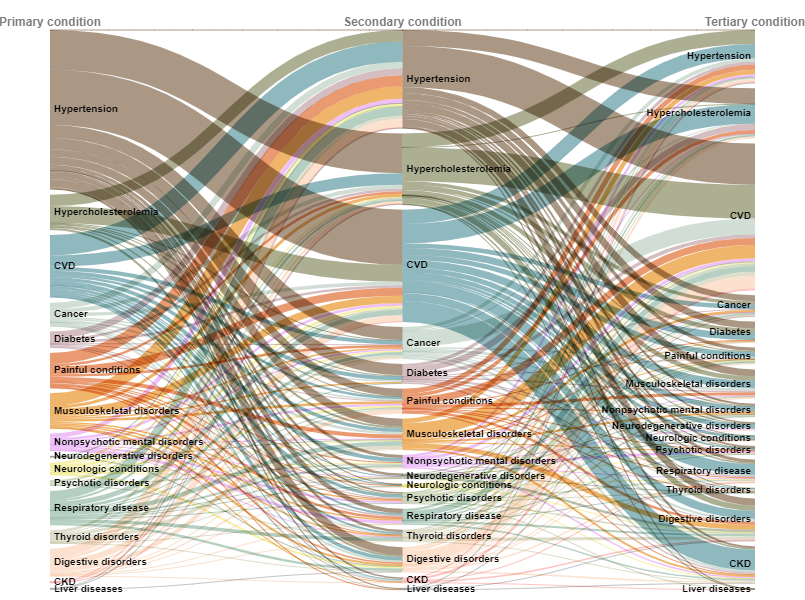


**Figure S18. Disease trajectory in the whole life-course among individuals who were diagnosed with three diseases before dying from cardiovascular disease**

Disease trajectory was computed based on the permutation of 16 groups of diseases according to the age at diagnosis of the diseases. Primary disease is the first one of diseases of interest diagnosed in life-course.


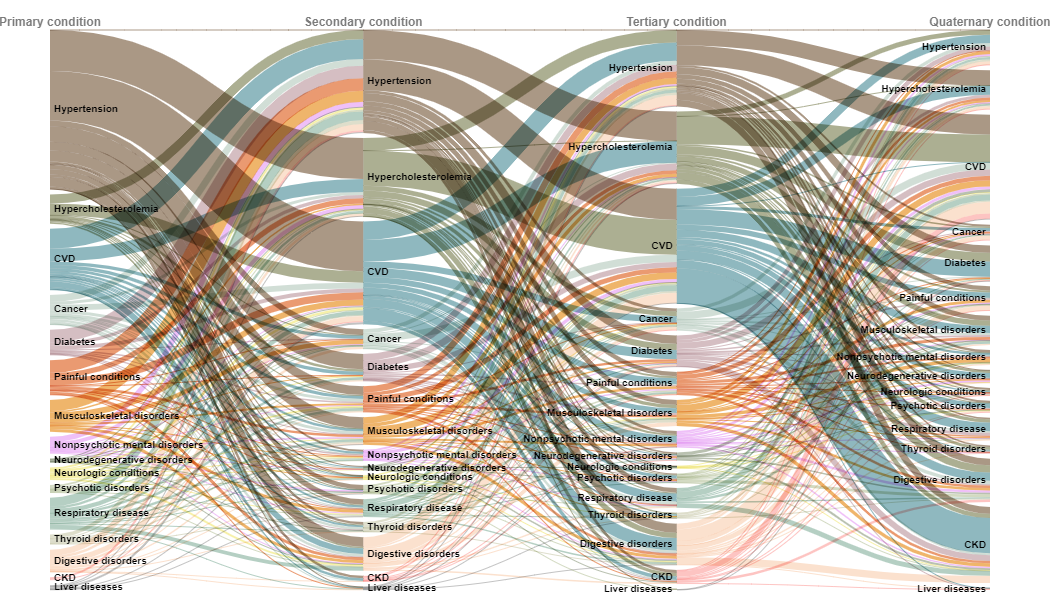


**Figure S19. Disease trajectory in the whole life-course among individuals who were diagnosed with four diseases before dying from cardiovascular disease**

Disease trajectory was computed based on the permutation of 16 groups of diseases according to the age at diagnosis of the diseases. Primary disease is the first one of diseases of interest diagnosed in life-course.


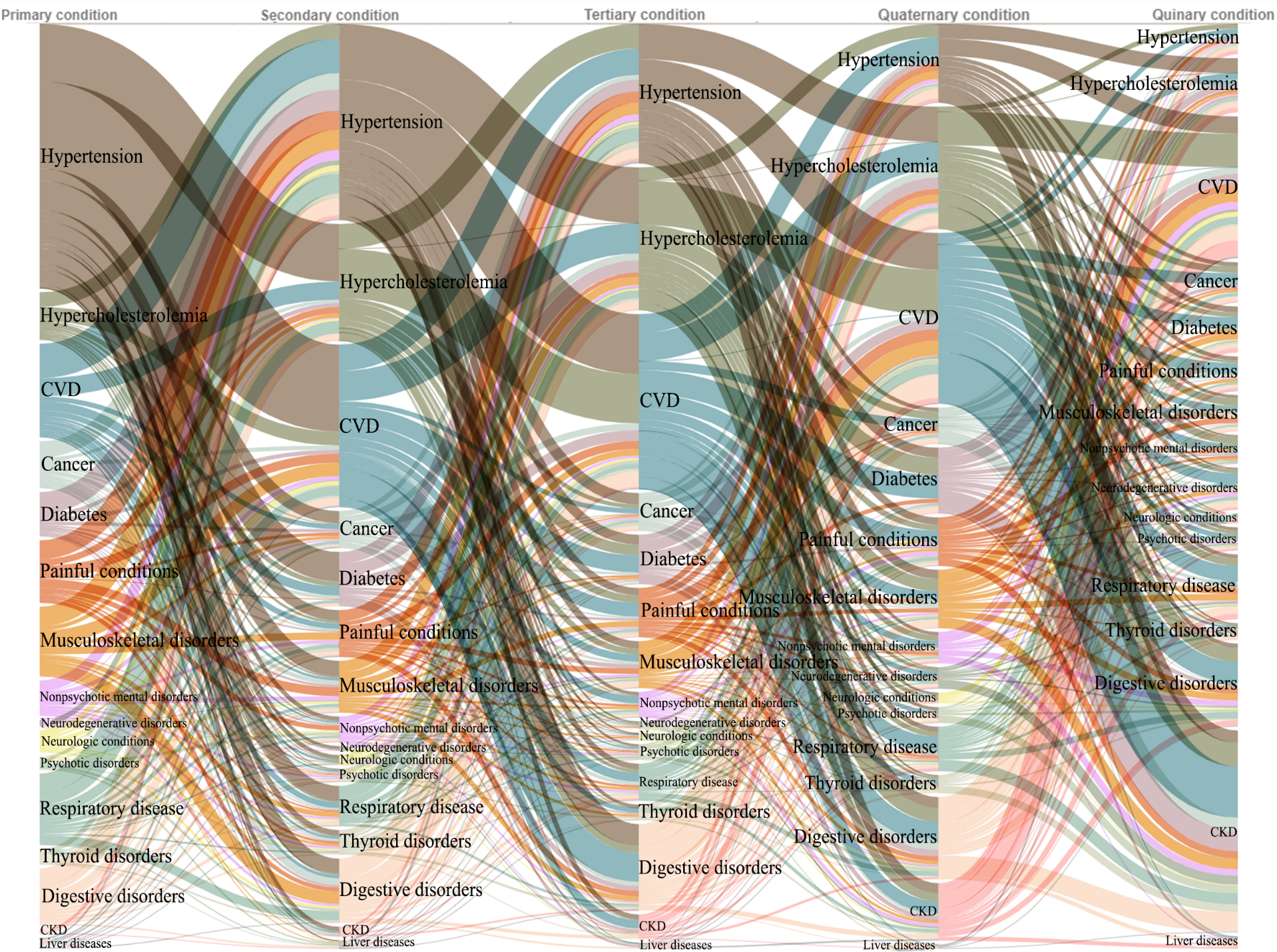


**Figure S20. Disease trajectory in the whole life-course among individuals who were diagnosed with five diseases before dying from cardiovascular disease**

Disease trajectory was computed based on the permutation of 16 groups of diseases according to the age at diagnosis of the diseases. Primary disease is the first one of diseases of interest diagnosed in life-course.


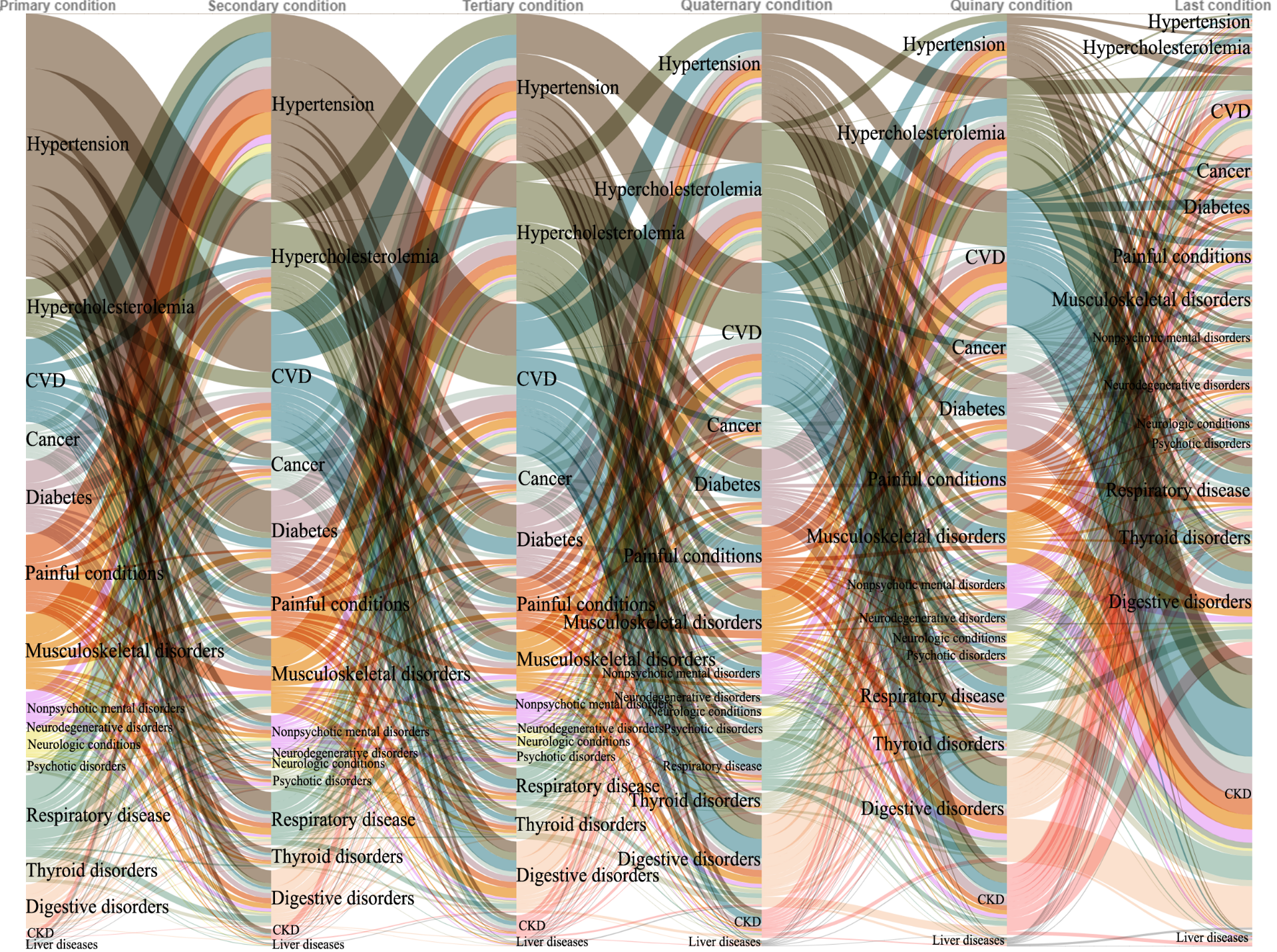


**Figure S21. Disease trajectory in the whole life-course among individuals who were diagnosed with six or more diseases before dying from cardiovascular disease**

Disease trajectory was computed based on the permutation of 16 groups of diseases according to the age at diagnosis of the diseases. Primary disease is the first one of diseases of interest diagnosed in life-course and last disease is the last one diagnosed before mortality. Diseases diagnosed from seventh until the last one was not taken into consideration among those who were diagnosed with seven or more diseases.
